# A genome-wide association study identifies multiple loci for variation in human ear morphology

**DOI:** 10.1038/ncomms8500

**Published:** 2015-06-24

**Authors:** Kaustubh Adhikari, Guillermo Reales, Andrew J. P. Smith, Esra Konka, Jutta Palmen, Mirsha Quinto-Sanchez, Victor Acuña-Alonzo, Claudia Jaramillo, William Arias, Macarena Fuentes, María Pizarro, Rodrigo Barquera Lozano, Gastón Macín Pérez, Jorge Gómez-Valdés, Hugo Villamil-Ramírez, Tábita Hunemeier, Virginia Ramallo, Caio C. Silva de Cerqueira, Malena Hurtado, Valeria Villegas, Vanessa Granja, Carla Gallo, Giovanni Poletti, Lavinia Schuler-Faccini, Francisco M. Salzano, Maria- Cátira Bortolini, Samuel Canizales-Quinteros, Francisco Rothhammer, Gabriel Bedoya, Rosario Calderón, Javier Rosique, Michael Cheeseman, Mahmood F. Bhutta, Steve E. Humphries, Rolando Gonzalez-José, Denis Headon, David Balding, Andrés Ruiz-Linares

**Affiliations:** 1 Department of Genetics, Evolution and Environment, UCL Genetics Institute, University College London, London WC1E 6BT, UK; 2 Centre for Cardiovascular Genetics, BHF Laboratories, Institute Cardiovascular Sciences, University College London, Rayne Building, London WC1E 6JF, UK; 3 Centro Nacional Patagónico, CONICET, Puerto Madryn U9129ACD, Argentina; 4 National Institute of Anthropology and History, Mexico City 4510, Mexico; 5 GENMOL (Genética Molecular), Universidad de Antioquia, Medellín 5001000, Colombia; 6 Instituto de Alta Investigación, Universidad de Tarapacá, Programa de Genética Humana ICBM Facultad de Medicina Universidad de Chile and Centro de Investigaciones del Hombre en el Desierto, Arica 1000000, Chile; 7 Unidad de Genómica de Poblaciones Aplicada a la Salud, Facultad de Química, UNAM-Instituto Nacional de Medicina Genómica, Mexico City 4510, Mexico; 8 Facultad de Medicina, UNAM, Mexico City 4510, Mexico; 9 Departamento de Genética, Universidade Federal do Rio Grande do Sul, Porto Alegre 91501-970, Brazil; 10 Laboratorios de Investigación y Desarrollo, Facultad de Ciencias y Filosofía, Universidad Peruana Cayetano Heredia, Lima 31, Peru; 11 Departamento de Zoología y Antropología Física, Universidad Complutense de Madrid, Madrid 28040, Spain; 12 Departamento de Antropología, Facultad de Ciencias Sociales y Humanas, Universidad de Antioquia, Medellín 5001000, Colombia; 13 The Roslin Institute and Royal (Dick) School of Veterinary Studies, University of Edinburgh, Midlothian EH25 9RG, UK; 14 UCL Ear Institute, University College London, London WC1X 8EE, UK; 15 Royal National Throat Nose and Ear Hospital, London WC1X 8EE, UK; 16 Schools of BioSciences and Mathematics & Statistics, University of Melbourne, Melbourne, Victoria 3010, Australia

## Abstract

Here we report a genome-wide association study for non-pathological pinna morphology in over 5,000 Latin Americans. We find genome-wide significant association at seven genomic regions affecting: lobe size and attachment, folding of antihelix, helix rolling, ear protrusion and antitragus size (linear regression *P* values 2 × 10^−8^ to 3 × 10^−14^). Four traits are associated with a functional variant in the Ectodysplasin A receptor (*EDAR*) gene, a key regulator of embryonic skin appendage development. We confirm expression of *Edar* in the developing mouse ear and that *Edar*-deficient mice have an abnormally shaped pinna. Two traits are associated with SNPs in a region overlapping the T-Box Protein 15 (*TBX15*) gene, a major determinant of mouse skeletal development. Strongest association in this region is observed for SNP rs17023457 located in an evolutionarily conserved binding site for the transcription factor Cartilage paired-class homeoprotein 1 (*CART1*), and we confirm that rs17023457 alters *in vitro* binding of *CART1*.

## Abstract (web summary)

The shape of the pinna varies widely in the general human population but the genetic basis of this variation is unknown. Here Adhikari *et al.* conduct a genome-wide association study in Latin Americans and discover seven gene regions influencing pinna morphology, including *EDAR* and *TBX15*.

## Graphical abstract

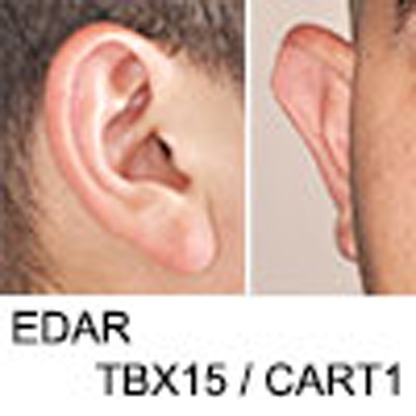

The human pinna is made up of a piece of cartilage covered with skin and attached to the skull by ligaments, muscles and fibrous tissue. This cartilage does not extend into the ear lobe, which consists mostly of areolar and adipose tissue. A range of disorders affecting human pinna development have been described, occurring in isolation or as part of complex syndromes with multiple affected organs^1^,^2^. There is also great non-pathological variation between humans in pinna shape and size, and this variation has been reported to be influenced by age, sex and ethnicity^3^,^4^,^5^. The study of rare, familial microtia cases (a disorder characterized by a small, abnormally shaped pinna) has established that mutations in the *HOXA2* gene can severely impact pinna development^6^. However, no genetic variants influencing normal pinna morphology have yet been reported. Here we aimed at identifying such variants by performing a genome-wide association study (GWAS) in a large sample of Latin American individuals with no pinna abnormalities. We identified seven loci with genome-wide significant association to variation in various pinna features, including several strong candidate genes with known developmental effects. We provide follow-up experimental evidence supporting the pinna morphology associations for two gene regions (which include the Ectodysplasin A receptor (*EDAR*) and the T-Box Protein 15 (*TBX15*) genes).

## Results

**Figure 1:**
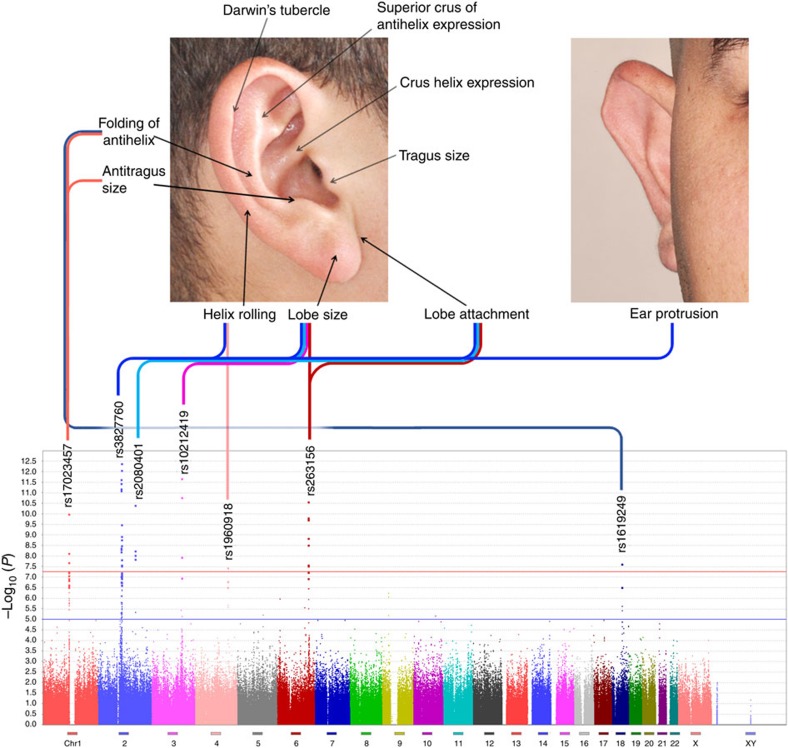
Genome-wide associations of pinna traits. Variation in 10 pinna traits was evaluated in 5,062 individuals. The photographs at the top indicate the location of the traits examined. At the bottom is shown a composite Manhattan plot for the seven traits showing genome-wide significant association with at least one genome region. The rs numbers for the most significantly associated (index) SNP in each region are provided (Table 1). Each of the seven regions on the Manhattan plot is connected with the associated trait on the photos via a line of different colour (composite panels in this and subsequent figures were made using Photoshop^49^).

Our study sample is part of the CANDELA (Consortium for the Analysis of the Diversity and Evolution of Latin America; http://www.ucl.ac.uk/silva/candela) cohort collected in five Latin American countries (Brazil, Colombia, Chile, Mexico and Peru) for the study of the genetics of physical appearance^7^. This sample consists of individuals of both sexes (median age 22 years), with a mixed African, European and Native American genetic background (Supplementary Table 1). Using facial photographs, we performed a qualitative assessment (on a three-point-ordered categorical scale; Fig. 1; Supplementary Figs 1 and 2) of 10 pinna traits in 5,062 individuals: ear protrusion, lobe size, lobe attachment, tragus size, antitragus size, helix rolling, folding of antihelix, crus helix expression, superior crus of antihelix expression and Darwin's tubercle.

### Variation in the pinna traits examined

The trait scores show a weak-to-moderate correlation between them, and with age, sex, body mass index (BMI) and genetic ancestry (Supplementary Table 2). Most noticeably, lobe attachment shows a moderate and significant (permutation *P* value <0.001) negative correlation with lobe size (*r*=−0.49). Significant but weaker positive correlations were observed for folding of antihelix with helix rolling (*r*=0.25) and with superior crus of antihelix expression (*r*=0.23), as well as between ear protrusion and helix rolling (*r*=0.16).

Individuals were genotyped on Illumina's Omni Express BeadChip. After quality control, 671,038 single-nucleotide polymorphisms (SNPs) and 4,919 individuals were retained for further analyses. Autosomal admixture proportions for the full sample were estimated as: 53% European, 43% Native American and 4% African (Supplementary Fig. 3). On the basis of a kinship matrix derived from the genome-wide SNP data^8^, we estimated narrow-sense heritability using GCTA^9^ and found moderate and significant values for all traits, with the highest heritability observed for ear protrusion (61%) and the lowest for tragus size (25%) (Supplementary Table 3). Similar heritabilities have been calculated for a range of facial traits using family data^10^,^11^.

### Primary association analyses

For the primary genome-wide association tests, we applied multivariate linear regression, as implemented in PLINK^12^, using an additive genetic model adjusting for: age, sex, height, BMI and the first five principal components (PCs) calculated from SNP data. The resulting statistics showed no evidence of residual population stratification for any of the traits (Supplementary Fig. 4). To account for the possibility of cryptic relatedness between individuals, we also performed genome-wide association tests using random-effects mixed linear regression models (using FastLMM^13^) and obtained similar results as in the PLINK analyses (Supplementary Table 4). Six of the traits examined showed genome-wide significant association (linear regression *P* values <5 × 10^−8^) with SNPs in at least one of the seven genomic regions (Fig. 1; Table 1). A global false discovery rate test across all traits and SNPs identified the same significantly associated regions (Supplementary Table 5). Lobe size is associated with SNPs in four regions (2q12.3, 2q31.1, 3q23 and 6q24.2); three traits show association with two regions: lobe attachment (2q12.3 and 2q31.1), helix rolling (2q12.3 and 4q31.3) and antihelix folding (1p12 and 18q21.2). The remaining two traits show association with a single region: ear protrusion (2q12.3) and antitragus size (1p12). Since the traits examined show some correlation between them, the associations detected are likely not fully independent. Most noticeably, the moderate negative correlation observed between lobe attachment and size is consistent with SNPs at the same two loci (2q12.3 and 2q31.1) showing significant association with both traits. Suggestive association with lobe attachment is also observed for 6q24.2, which is significantly associated with lobe size (Table 1).

### Secondary analyses

**Figure 2:**
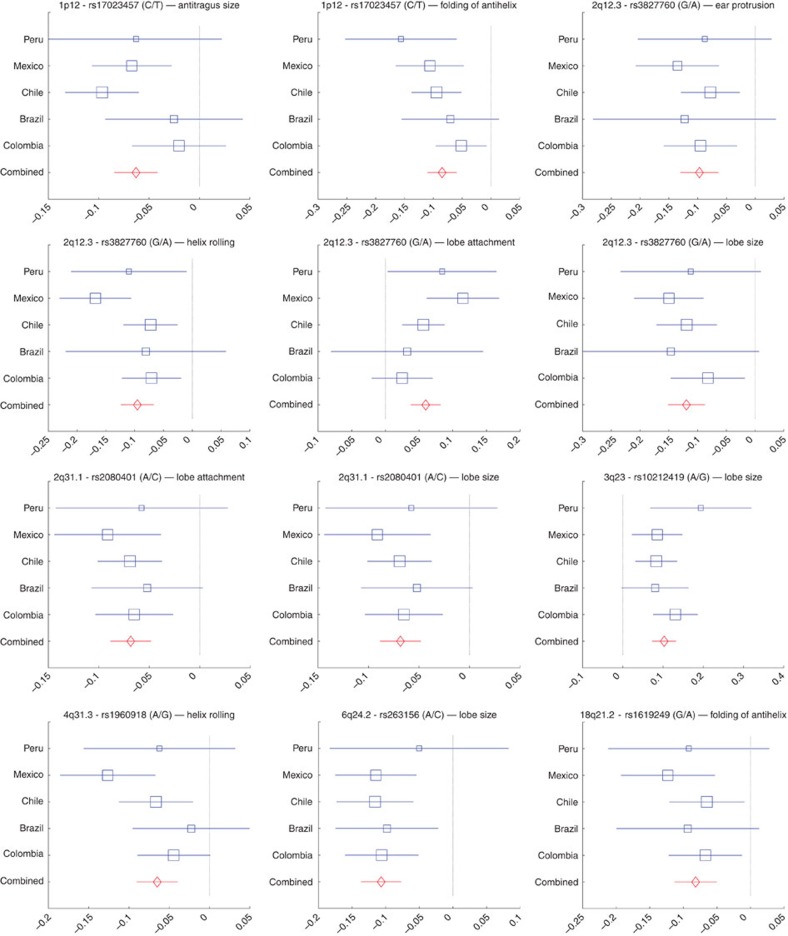
Meta-analysis of significant genome-wide associations. Effect sizes (in each country sample and in a combined meta-analysis) for the index SNPs and their associated traits (Table 1). Regression coefficients (*x* axis) estimated in each country are shown as blue boxes (box size indicating sample size). Red diamonds indicate effect sizes estimated in the meta-analysis. Horizontal bars indicate s.e. Results for all the SNPs and traits shown in Table 1 are provided in Supplementary Fig. 6A. The two alleles at each SNP are shown in brackets with effect size referring to the allele in the numerator.

Since correlations between traits could have a shared underlying genetic basis, we performed a multivariate analysis combining all phenotypes in a single regression model (using MULTIPHEN^14^). This analysis identified the same set of regions as in the independent regression tests, but no additional associated regions (Supplementary Fig. 5). We also examined the association signals for all index SNPs (Table 1) in each country separately and combined results as a meta-analysis using METAL^15^. For each association, the effects were in the same direction in all countries, the variability of effect size across countries reflecting sample size (Fig. 2; Supplementary Table 6; Supplementary Fig. 6a). There was no significant evidence of effect size heterogeneity across countries for any of the associations. A full genome-wide meta-analysis did not reveal additional regions showing significant association with pinna morphology (Supplementary Fig. 6b). The seven index SNPs of Table 1 provide modest phenotypic prediction accuracy (Supplementary Table 7). The fraction of the phenotypic variance explained by these SNPs is small relative to the heritability estimates (Supplementary Tables 3 and 7), suggesting a polygenic architecture for these traits beyond that captured by the genome-wide significance threshold used.

### Features of associated regions

The genomic regions showing genome-wide significant association have features with independent evidence suggestive of an involvement in pinna development. This evidence is particularly strong for the regions in 2q12.3 and 1p12, and we followed-up these two regions with additional experiments.

### 2q12.3

**Figure 3:**
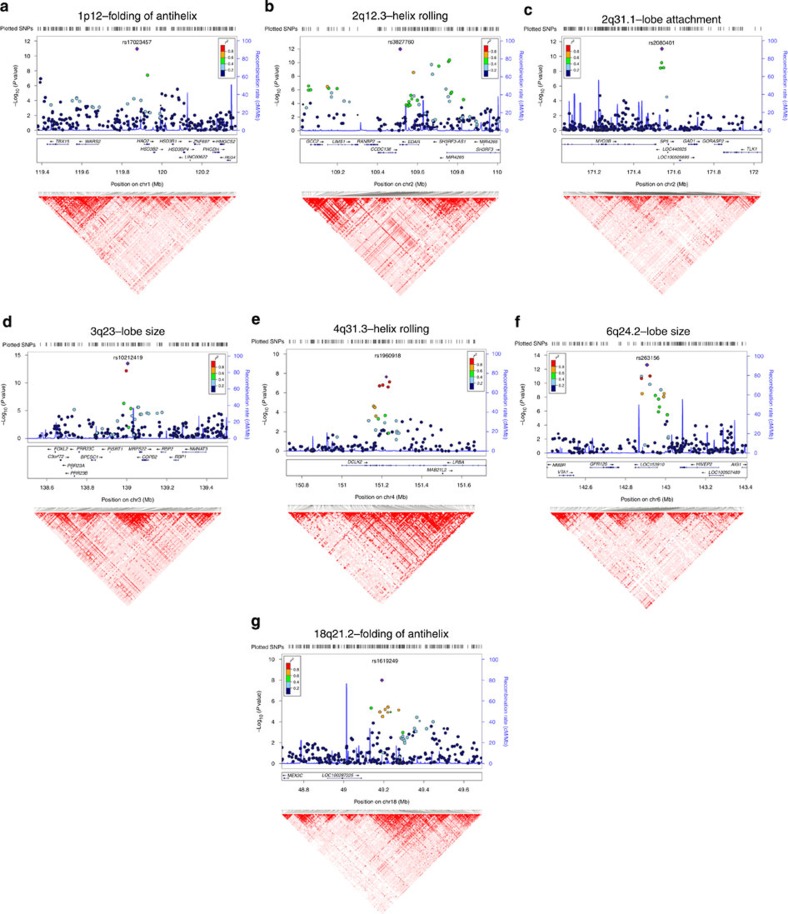
LocusZoom and linkage disequilibrium plots of significantly associated genetic regions. Plots of the seven genomic regions showing genome-wide significant association to pinna traits (Table 1). For regions showing association with several pinna traits, we present here only results for the trait with strongest association (plots for the other associated traits are presented in Supplementary Fig. 7). Association results from a multivariate linear regression model (on a −log_10_ *P* scale; left *y* axis) are shown for SNPs ∼500 kb on either side of the index SNP (that is, the SNP with the smallest *P* value, purple diamond; Table 1) with the marker (dot) colour indicating the strength of LD (*r*^2^) between the index SNP and that SNP in the 1000 Genomes AMR data set. Local recombination rate in the AMR data is shown as a continuous blue line (scale on the right *y* axis). Genes in each region, their intron–exon structure, direction of transcription and genomic coordinates (in Mb, using the NCBI human genome sequence, Build 37, as reference) are shown at the bottom. Plots were produced with LocusZoom^56^. Below each region we also show an LD heatmap (using D′, ranging from red indicating D′=1 to white indicating D′=0) produced using Haploview^57^. Note that the location of SNPs on the LD heatmap can be shifted relative to the regional display on top of it.

Includes SNPs associated with four traits (lobe size, lobe attachment, helix rolling and ear protrusion; Table 1). These SNPs extend over ∼500 kb and show substantial linkage disequilibrium (LD; Fig. 3b). Strongest association with all four traits was found for SNP rs3827760 (Table 1), and conditioning on this SNP abolishes the signal of association at other SNPs in the region (Supplementary Fig. 8a). Marker rs3827760 leads to a functional p.Val370Ala substitution in the intracellular death domain of EDAR. This residue affects the interaction with the *EDAR*-binding death domain adapter protein *EDARADD*^16^; the derived *EDAR* allele encodes a protein with higher activity than the ancestral one^17^,^18^. *EDAR* signalling acts during prenatal development to specify the location, size and shape of ectodermal appendages, such as hair follicles, teeth and glands^19^. The p.Val370Ala variant has been associated with characteristic tooth morphologies, hair type and sweat gland density in East Asians^20^,^21^,^22^,^23^,^24^,^25^, where this allele is present at high frequency while being nearly absent in European or African populations (Supplementary Table 8). Consistent with these effects in humans, mice expressing *EDAR370A*, or with increased *EDAR* function, show thickened and straightened hair fibres^17^,^20^,^24^,^25^.

**Figure 4:**
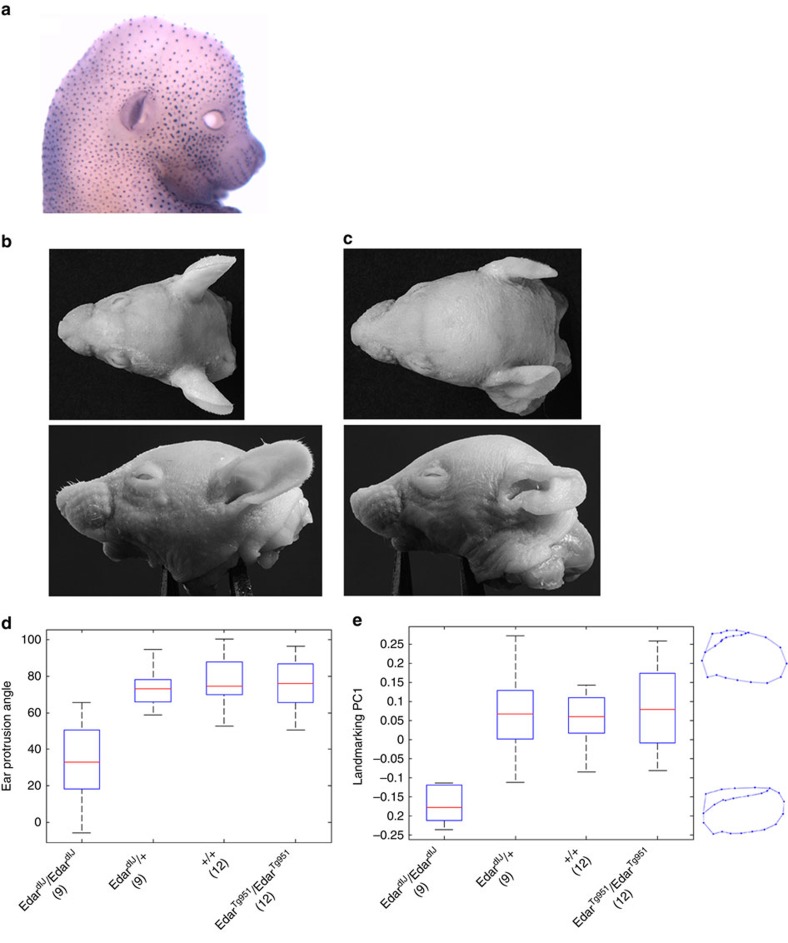
Effect of *Edar* genetic variation on mouse pinna shape. (**a**) Whole-mount *in situ* hybridization detecting *Edar* expression in the developing mouse embryo at 15 days' gestation. (**b**–**e**) Impact of *Edar* genotype on mutant mouse ear shape. (**b**,**c**) Photographs of wild-type (**b**) and homozygous *Edar*^*dlJ*^ (**c**) mutant mice from top and side views (respectively, on the upper and lower panels). (**d**,**e**) Boxplots, respectively, of ear protrusion angle and of landmark coordinate PC1 (*y* axis) for different mouse genotypes (*x* axis) (Supplementary Note 1; Supplementary Fig. 10 shows additional analyses for ear protrusion). Boxplot whiskers extend to data points within 1.5 times the interquartile range on both sides. In **d**,**e** numbers in parenthesis below genotypic categories refer to the number of mice examined for each. On the right of **e** are shown average PC1 wireframes for *Edar*^*dlJ*^ homozygous mice (bottom) or for mice with other genotypes (top).

We examined *Edar* expression in the developing mouse embryo (Fig. 4a). The structure of the pinna is defined prenatally in both mouse and human, in mouse primarily between gestation days 13 and 16 (ref. 2). Using whole-mount *in situ* hybridization, we confirmed *Edar* expression along the distal margin of the developing pinna (Fig. 4a), in addition to the well-characterized expression in the developing hair follicles^16^. *Edar* expression at the distal margin of the embryonic pinna may aid in determining its growth and expansion, thus influencing the ultimate form adopted by the ear. To assess the functional significance of this *Edar* expression during pinna development, we examined postnatal pinna morphology in *Edar* mouse mutants. The *Edar*^*dlJ*^ (ref. 26) and *Edar*^*Tg951*^ (ref. 16) mouse lines have a loss and a gain of *Edar* function, respectively (see Methods). At 2 weeks of age, the pinna of homozygous *Edar*^*dlJ*^ mice have a characteristic shape, including a marked dorsal/anterior folding (Fig. 4c). Quantitative assessment of mouse pinna protrusion (assessed by measuring protrusion angle) and shape (assessed by PC analysis of two-dimensional landmark coordinates) revealed significant differences (linear regression *P* value <0.0007; Supplementary Note 1; Supplementary Figs 9 and 10) between the homozygous *Edar*^*dlJ/dlJ*^ mutant and heterozygous *Edar*^*dlJ/+*^ littermates, wild-type mice and *Edar*^*Tg951*^ mice. Landmark coordinate PC1, capturing 69% of the variation in shape, reflects mainly a change in the extent of helix rolling of the mouse pinna (Fig. 4e; Supplementary Fig. 10d), consistent with one of the effects we observed of *EDAR* variants on human pinna shape. The *Edar* high copy-number transgenic (*EdarTg951*) does not display a detectable change in helix rolling likely due to the fact that the wild-type mouse pinna does not have a prominent helix roll, thus hampering the detection of any further pinna flattening that might be caused by increased *Edar* function.

### 1p12

SNPs in this region show genome-wide association with antihelix folding and antitragus size. This region extends over ∼800 kb and overlaps the gene encoding transcription factor *TBX15* (Fig. 3a), a key regulator of cartilaginous and skeletal development in the mouse^27^. A spontaneous *Tbx15* mouse mutant (called ‘droopy ears'), is characterized by altered positioning, projection and shape of the pinnae^28^,^29^. In humans, mutations of *TBX15* result in Cousin syndrome, a disorder characterized by craniofacial dysmorphism, including a dysplastic pinna^30^. Strongest association in this region was observed for intergenic SNP rs17023457 (*P* value 2 × 10^−8^ for antitragus size and 1 × 10^−11^ for antihelix folding Table 1) and conditioning on this SNP abolishes the signal of association at other markers (Supplementary Fig. 8b). Interestingly, rs17023457 is located in a highly conserved binding site for transcription factor *CART1* (cartilage paired-class homeoprotein 1) (Supplementary Fig. 11a), mutations of which have been shown to result in a range of craniofacial and cartilage abnormalities in the mouse^31^. The location of rs17023457 in a *CART1*-binding site strongly suggests that this SNP could directly influence the expression of neighbouring genes involved in cartilaginous development, such as *TBX15.*

To assess the potential for rs17023457 to alter DNA–protein interactions involving the *CART1*-binding site, electrophoretic mobility shift assays were performed using nuclear extracts from a *CART1*-expressing cell line (Huh7). Double-stranded oligonucleotides containing the rs17023457 T allele demonstrated binding of a nuclear protein, this binding being undetectable for oligonucleotides carrying the derived C allele (Supplementary Fig. 11b). Binding was eliminated upon prior incubation with excess unlabelled *CART1* consensus sequence DNA, confirming the specificity of the assay and supporting the ability of the sequence containing the T allele to bind *CART1 in vitro*. Reporter constructs containing either the rs17023457 T or C alleles driving the expression of the luciferase gene, showed a decreased expression of 36 and 22% in constructs with the C allele when the genomic sequence was positioned in the forward and reverse orientation, respectively (Supplementary Fig. 11b). These data indicate that *CART1*, or other nuclear DNA-binding proteins with the same sequence specificity, is able to bind to an enhancer that includes rs17023457, with variation at this SNP determining whether binding and full transactivation occurs or not. The implications of these observations for the *in vivo* regulation of *TBX15* expression remain to be established.

### Other regions

For the other regions showing genome-wide association, there is currently less compelling information suggestive of a mechanism explaining their effect on pinna morphology, and we did not attempt their experimental follow-up. SNPs in 3q23 are associated with lobe size, with strongest association being seen for intergenic SNP rs10212419 (*P* value 3 × 10^−14^), in a region with substantial LD over ∼500 kb (Fig. 3d). Intriguingly, considering that the ear lobe is made up mainly of loose connective tissue, intergenic SNPs in this region have been strongly associated with keloid formation^32^, an exaggerated skin wound-healing reaction characterized by excessive deposition of extracellular matrix and collagen fibres. Some highly penetrant mutations across this genomic region have also been found to result in alterations of craniofacial development involving pinna morphology. The gene nearest to SNP rs10212419 (∼59 kb away) encodes mitochondrial ribosomal protein S22 (*MRPS22*), mutations of which can lead to combined oxidative phosphorylation deficiency 5 (*COXPD5*), a disorder whose phenotype can include low-set, posteriorly rotated ears (OMIM #611719). A report of a patient with a similar ear phenotype found a non-synonymous substitution at an evolutionary conserved site within *MRPS22* (ref. 33). Another interesting candidate gene in the vicinity is that encoding the forkhead box L2 transcription factor (*FOXL2*). Coding and regulatory mutations of this gene cause BPES (blepharophimosis, ptosis, epicanthus inversus syndrome)^34^,^35^, a disorder characterized by a range of craniofacial abnormalities, including alterations in ear lobe morphology^36^.

The 2q31.1 region shows considerable LD over ∼100 kb and is associated with lobe attachment and size, with strongest association seen for intergenic SNP rs2080401 (*P* values of 9 × 10^−12^ and 1 × 10^−10^ for these two phenotypes, respectively; Fig. 3c). This SNP is located ∼31 kb upstream of the gene encoding Specificity Protein 5 (SP5), a Sp1-related transcription factor that mediates responses to Wnt/beta-catenin signalling. Of potential interest, there is evidence of an involvement of this pathway in musculo-skeletal development^37^,^38^. The region in 6q24.2, associated with lobe size, shows substantial LD over ∼500 kb overlapping the hypothetical protein-coding gene *LOC153910*. Strongest association was seen for intronic rs263156 (*P* value 2 × 10^−13^; Fig. 3f). Potentially of relevance to ear development, about 100 kb from rs263156 is *GPR126* (G protein-coupled receptor 126), a gene strongly associated with human height^39^,^40^,^41^. Borderline genome-wide significance was found for rs1960918 in 4q31.3 (*P* value 2 × 10^−8^ for helix rolling; Fig. 3e) and rs1619249 in 18q21.2 (*P* value 1 × 10^−8^ for antihelix folding; Fig. 3g). Intronic marker rs1960918 is in an LD region of ∼400 kb overlapping the *LRBA* gene, whose product (LPS-responsive vesicle trafficking, beach and anchor containing) is known to be involved in coupling signal transduction, vesicle trafficking and immunodeficiency, with no obvious functional connection to pinna development. Intergenic SNP rs1619249 is in an LD region of about 300 kb, the closest candidate being LOC100287225 (about 100 kb from rs1619249), a hypothetical gene of unknown function.

## Discussion

In conclusion, we have identified the first genetic variants influencing normal variation in human pinna morphology. It will be important to evaluate further the role of these regions in ear development and its disorders. Since pinna morphology in mammals shows extensive evidence of evolutionary adaptation, particularly in relation to thermoregulation, acoustic perception and sound localization^42^,^43^,^44^,^45^, it will be interesting to examine whether variation in these genomic regions relates to adaptive changes in the pinna across species. Finally, there is increasing interest in the use of pinna variation in forensics^45^,^46^, and development of such applications may benefit from a refined knowledge of the genetic determinants of pinna morphology.

## Methods

### Study subjects

In total, 5,062 volunteers from 5 Latin American countries (Brazil, Chile, Colombia, Mexico and Peru), part of the CANDELA consortium sample (http://www.ucl.ac.uk/silva/candela)^7^, were included in this study. Ethics approval was obtained from: Universidad Nacional Autónoma de México (Mexico), Universidad de Antioquia (Colombia), Universidad Perúana Cayetano Heredia (Peru), Universidad de Tarapacá (Chile), Universidade Federal do Rio Grande do Sul (Brazil) and University College London (UK). All participants provided written informed consent. Blood samples were collected by a certified phlebotomist and DNA extracted following standard laboratory procedures. Five digital photographs of the face: left side (−90°), left angle (−45°), frontal (0°), right angle (45°) and right side (90°) were taken from ∼1.5 m at eye level using a Nikon D90 camera fitted with a Nikkor 50 mm fixed focal length lens. Other phenotypes including height, weight, BMI, age and sex were also recorded for each participant^7^.

### Pinna phenotyping

Right side, right angle and frontal photographs were used to score 10 pinna traits. These were (Fig. 1): ear protrusion, lobe size, lobe attachment, tragus size, antitragus size, helix rolling, folding of antihelix, crus helix expression, superior crus of antihelix expression and Darwin's tubercle. Each trait was scored as an ordered categorical variable, with 0 being the lowest level of expression of the trait and 2 the highest (Supplementary Fig. 1). Software to assist scoring of photographs was developed in MATLAB^47^. Intraclass correlation coefficients^48^ calculated by double-scoring photographs of 100 subjects indicate a moderate-to-high intra- and inter-rater reliability of the trait scores (Supplementary Table 9). Photographs for all the volunteers were scored by the same rater (G.R.).

### DNA genotyping and quality control

DNA samples from participants were genotyped on the Illumina HumanOmniExpress chip including 730,525 SNPs. PLINK^12^ was used to exclude SNPs and individuals with >5% missing data, markers with minor allele frequency <1% and related individuals. After applying these filters, 680,634 SNPs were retained for further analysis. Owing to the admixed nature of the study sample (Supplementary Fig. 3), there is an inflation in Hardy–Weinberg *P* values. We therefore did not exclude markers based on the Hardy–Weinberg deviation.

### Statistical analyses

*P* values for Pearson correlation coefficients were obtained by permutation. All regressions were performed using an additive multivariate linear or logistic model, providing *P* values that are obtained from the standard *t*-statistic derived from their regression coefficients. Narrow-sense heritability (defined as the phenotypic variance explained by a genetic relatedness matrix, GRM, computed from the SNP data) for the pinna traits examined was estimated using GCTA^9^ by fitting an additive linear model with a random-effect term whose variance is given by the GRM, with age, sex, height and BMI as covariates. The GRM was obtained using the LDAK approach^8^, which accounts for LD between SNPs. An LD-pruned set of 93,328 autosomal SNPs was used to estimate continental ancestry using ADMIXTURE^49^ (Supplementary Fig. 3). PLINK^12^ was used to perform the primary genome-wide association tests for each phenotype using multiple linear regression with an additive genetic model incorporating age, sex, height, BMI and 5 (or 10) genetic PCs as covariates. These genetic PCs were obtained (using MATLAB^47^) from an LD-pruned set of 93,328 SNPs. Inspection of the scree plot (Supplementary Fig. 4), and the PC scatterplots (not shown), indicates that only the first 5 PCs show evidence of substructure. Consistent with this, PLINK results with 5 or 10 PCs were nearly identical. The *Q*–*Q* plots for all PLINK analyses showed no sign of inflation, the genomic control factor lambda being <1.03 in all cases. To account for multiple testing, we also applied (using MATLAB^47^) a global FDR^50^ test across all traits and SNPs (Supplementary Table 5). To account for the possibility that cryptic relatedness among the individuals studied could affect the linear regression results, we also performed a GWAS using mixed-effects regression models, as implemented in FastLMM^13^. In this approach, the GRM again specifies the variance of a random effect (other aspects of the regression being as for the simpler model used by PLINK). Results from these random-effect regressions were similar to those obtained in the primary PLINK analyses (Supplementary Table 4). To consider the correlation between pinna traits, we performed a GWAS combining all traits in a single analysis using a multivariate regression model as implemented in MULTIPHEN^14^ on an LD-pruned data set (Supplementary Fig. 5). In this approach, a SNP genotype is taken as the dependent variable and all phenotypes are jointly taken as covariates. Due to this increased complexity the runtime per SNP is considerably longer, so an LD-pruned subset of 189,707 SNPs was used for this analysis (ensuring that all genome-wide or suggestive SNPs from the primary analyses are included). A meta-analysis (Supplementary Table 6; Supplementary Fig. 6) was carried out for each of the traits by performing a GWAS separately in each country sample (using PLINK as above) and combining the results in METAL^15^. Forest plots were produced with MATLAB combining all regression coefficients and s.e. Cochran's *Q*-statistic was computed for each trait to test for effect size heterogeneity across country samples. Prediction of trait phenotypes from genotypes at the seven index SNPs identified in the primary GWAS analysis (Table 1) was performed in MATLAB^47^ using linear regression and nonlinear neural network models^51^ (Supplementary Table 7).

### Whole-mount *in situ* hybridization of mouse embryos

Embryos were fixed in 4% paraformaldehyde in PBS at 4 °C overnight, dehydrated to methanol, rehydrated, treated with proteinase K, refixed and hybridized to a digoxigenin-labelled cRNA covering the entire *Edar* transcript (AF160502). Hybridization was performed in 50% formamide, 750 mM NaCl, 75 mM Na_3_C_6_H_5_O_7_, 1% SDS containing 50 μg ml^−1^ yeast RNA and 50 μg ml^−1^ heparin at 65 °C overnight. After washing and blocking of embryos in 10% sheep serum, the signal was detected using 1/2,000 sheep anti-digoxigenin conjugated to alkaline phosphatase (sheep anti-digoxigenin alkaline phosphatase antibody: 11093274910, Roche Applied Science, Mannheim, Germany) followed by NBT (nitro-blue tetrazolium chloride) and BCIP (5-bromo-4-chloro-3′-indolyphosphate p-toluidine) staining in 100 mM NaCl, 100 mM Tris pH 9.5, 50 mM MgCl_2_ and 0.1% Tween 20.

### Animals and sample collection

Animal studies were reviewed and approved by The Roslin Institute Animal Welfare and Ethical Review Body. The humane care and use of mice (*Mus musculus*) in this study was carried out under the authority of the appropriate UK Home Office Project License. Forty-two mouse head samples, 18 male and 24 female, were included in the analysis aggregating over four genotype categories. All mice were on the FVB/N strain background. The loss-of-function *Edar*^*dlJ*^ line carries the downless^Jackson^ allele, encoding *Edar* p. E379K. The gain-of-function *Edar*^*Tg951*^ line carries ∼16 copies of the entire *Edar* locus on a 200-kb yeast artificial chromosome (thus, homozygous transgenic animals carry about 34 copies of the gene in total)^17^. Homozygous transgenic animals were used in this study. Fourteen- and 15-day-old animals were weighed and culled by intraperitoneal administration of pentobarbitone. After measurement of body length, heads were depilated using Veet cream (Reckitt Benckiser, Slough, UK), rinsed with water and stored in 2% formaldehyde in PBS.

The variation in age and sex helped to collect animals of more diverse weights and sizes, so that we could control more readily for effects of size and weight on pinna shape. Photographs of superior and lateral views of the head were taken and were blinded prior to landmarking for analysis. A scale was included in the photographs for calibration. Landmark homology across specimens was achieved by controlling the head orientation in all photos (for example, by placing the sagittal plane of the head orthogonal to the anterior–posterior axis of the camera lens). This fixed orientation avoids rolling, heading and pitching rotations and guarantees the coplanarity of landmarks placed on the pinnae (see below).

### Mouse pinna phenotyping

Overall, pinna shape variation was examined using geometric morphometric techniques^52^. Twenty-seven landmarks and semilandmarks (depicted in Supplementary Fig. 8) were digitized, scaled and processed using TPSDig and TPSUtil (http://life.bio.sunysb.edu/morph/). Semilandmarks were placed along the contour of the pinna and other contours of interest, and the TPSRelW (http://life.bio.sunysb.edu/morph/) routine was used to allow semilandmarks to slide so as to minimize bending energy. Reliability of pinna landmarking was evaluated by scoring the same landmarks by a second rater and examining the variation (Supplementary Table 10). Generalized procrustes analysis was used to remove the effects of translation, rotation and scaling^52^. After superimposition of the generalized procrustes analysis-adjusted landmark coordinates, only the shape component remained in the aligned specimens. The superimposed coordinates were exported to the software MorphoJ^53^ to obtain PCs of shape. Ear protrusion was defined in two ways: angle between the two protruding pinnae, and distance between the two pinnae tips as a proportion of head width (Supplementary Fig. 9). The effect of *Edar* genotype on pinna shape and protrusion was tested using multivariate regression analysis in R^54^, adjusting for age, sex, body length, body weight, head length and head width. Two regression models were tested using two different genotype encodings (Supplementary Note 1): one with four distinct genotype categories and one considering genotypes as a binary variable, based on the notion that the *Edar* loss-of-function homozygous genotype (*Edar*^*dlJ*^/*Edar*^*dlJ*^) is recessive.

### Human tissue culture for the CART1-binding assay

The human hepatoma Huh7 (ECACC, UK) cell line was grown in high-glucose DMEM (PAA) supplemented with 2 mM L-glutamine and 10% fetal bovine serum and maintained in 5% CO_2_ at 37 °C. Nuclear extracts were obtained from Huh7 cells using the NE-PER Nuclear and Cytoplasmic Extraction Reagents Kit (Pierce, USA) as described in the manual, with the addition of Complete Protease Inhibitor (Roche, UK) to buffers CER I and NER I.

### Electrophoretic mobility shift assay

Probe sequences:

rs17023457 T>C

rs17023457 T forward 5′-ACTAACTAATCAACATTCCTTTGCGAATACA-3′

rs17023457 T reverse 5′-TGTATTCGCAAAGGAATGTTGATTAGTTAGT-3′

rs17023457 C forward 5′-ACTAACTAATCAACACTCCTTTGCGAATACA-3′

rs17023457 C reverse 5′-TGTATTCGCAAAGGAGTGTTGATTAGTTAGT-3′

*CART1* consensus F 5′-CCACATAATTACATTATCTTG-3′

*CART1* consensus R 5′-CAAGATAATGTAATTATGTGG-3′.

Probes were labelled using the Biotin 3′-End DNA Labelling Kit (Pierce) as described in the manufacturer's manual. Binding reactions consisted of 2 μl 10 × binding buffer (100 mM Tris, 500 mM KCl; pH 7.5), 1 μg p[dI-dC], 200 fmol biotin-labelled DNA, made to a total of 20 μl with H_2_O and incubated at 25 °C for 30 min, followed by the addition of 5 × loading buffer. Competition reactions were performed with 30-min incubation on ice, prior to addition of labelled probes, using 100 × CART1 cold-competitor oligonucleotides. Samples were run on 6% polyacrylamide gel, electrophoresed for 150 min at 120 V.

### Luciferase reporter assay

Luciferase reporter vectors were based on the pGL3 Promoter vector (Promega), with a 872-bp genomic sequence surrounding rs17023457 (Supplementary Fig. 11c) inserted upstream of the SV40 promoter in both orientations using the following primers designed for the InFusion PCR Cloning kit (Clontech):

Forward

Ear1FF: 5′-TCTATCGATAGGTACCCATCTACGGGTCTGGAGGAG-3′

Ear1FR: 5′-GATCGCAGATCTCGAGAGCTCATCCAAGGTCCCAAA-3′

Reverse

Ear2RF: 5′-GATCGCAGATCTCGAGCATCTACGGGTCTGGAGGAG-3′

Ear2RR: 5′-TCTATCGATAGGTACCAGCTCATCCAAGGTCCCAAA-3′.

Site-directed mutagenesis was performed using the QuickChange Lightning Site-Directed Mutagenesis Kit (Agilent Technologies) as detailed in the manual, using the following oligonucleotide sequences (designed for reverse strands):

Primer name; primer sequence (5′–3′)

SDMFFC 5′-GTATTCGCAAAGGAGTGTTGATTAGTTAGTGGTCGGTATCAT-3′

SDMFRC 5′-ATGATACCGACCACTAACTAATCAACACTCCTTTGCGAATAC-3′

Primer name; primer sequence (5′–3′)

SDMRFG 5′-GACCACTAACTAATCAACACTCCTTTGCGAATACAGCTTCAG-3′

SDMRRG 5′-CTGAAGCTGTATTCGCAAAGGAGTGTTGATTAGTTAGTGGTC-3′

All constructs were verified by direct sequencing.

Huh7 cells were seeded at a density of 2 × 10^4^ per well, in a 96-well plate and grown to confluence overnight in the appropriate media (described above). Cells were transfected with 250 ng pGL3 reporter construct, with 10 ng pRLTK as a transfection control. Transfection was carried out in Opti-Mem serum-free media (Sigma, UK) using Lipofecamine 3000 (Invitrogen) as described in the manual. Media was replaced 24 h following transfection, with serum-containing media described above, and the cells left for 2 days before harvesting. Cells were lysed using Passive Lysis Buffer (Promega) and luciferase expression was determined using the Dual Luciferase Reporter Assay System (Promega), and measured in the Tropix TR717 Microplate Luminometer (PE Applied Biosystems, UK). For each construct used, the transfection assay was performed using 12 wells of a 96-well plate and the mean luciferase reading reported. The experiment was repeated in triplicate using fresh vector preparations. Analysis of variance was performed to compare the effect of the T and C alleles on the luciferase readings, taking into account the variability across replicates and wells.

## Additional information

**How to cite this article:** Adhikari, K. *et al.* A genome-wide association study identifies multiple loci for variation in human ear morphology. *Nat. Commun.* 6:7500 doi: 10.1038/ncomms8500 (2015).

## Supplementary Material

Supplementary Information Supplementary Figures 1-11, Supplementary Tables 1-10, Supplementary Note 1 and Supplementary References

## Figures and Tables

**Table 1:** Chromosomal location and −log_10_ (*P*) for index SNPs showing strongest genome-wide association to pinna traits.

SNP, single-nucleotide polymorphism. Trait acronyms (Fig. 1): AS, antitragus size; CHE, crus helix expression; DT, Darwin's tubercle; EP, ear protrusion; FoA, folding of antihelix; HR, helix rolling; LA, lobe attachment; LS, lobe size; SCoAE, superior crus of antihelix expression; TS, tragus size.

Genome-wide significant values (–log_10_ (*P*)>7.3) are highlighted in the darkest shade of red. Below this significance threshold, intensity of colour background is proportional to −log10 (*P* value). Effect sizes for associated alleles are shown in Fig. 2 and Supplementary Table 5. Intragenic SNPs are shown in bold. In 1p12, SNP rs17023457 is in an evolutionary conserved binding site for the CART1 transcription factor. GWAS *P* values were obtained using an additive multivariate regression model as described in Methods.

## References

[b1] Gleeson M. J. & Clarke R. C. Scott-Brown's Otorhinolaryngology: Head and Neck Surgery CRC Press (2008).

[b2] Cox T. C., Camci E. D., Vora S., Luquetti D. V. & Turner E. E. The genetics of auricular development and malformation: new findings in model systems driving future directions for microtia research. Eur. J. Med. Genet. 57, 394–401 (2014). 24880027 10.1016/j.ejmg.2014.05.003 PMC4143470

[b3] Azaria R., Adler N., Silfen R., Regev D. & Hauben D. J. Morphometry of the adult human earlobe: a study of 547 subjects and clinical application. Plast. Reconstr. Surg. 111, 2398–2402 discussion 2403–2404 (2003). 12794488 10.1097/01.PRS.0000060995.99380.DE

[b4] Sforza C. *et al.* Age- and sex-related changes in the normal human ear. Forensic Sci. Int. 187, 110 e1–110 e7 (2009). 19356871 10.1016/j.forsciint.2009.02.019

[b5] Alexander K. S., Stott D. J., Sivakumar B. & Kang N. A morphometric study of the human ear. J. Plast. Reconstr. Aesthet. Surg. 64, 41–47 (2011). 20447883 10.1016/j.bjps.2010.04.005

[b6] Minoux M. *et al.* Mouse Hoxa2 mutations provide a model for microtia and auricle duplication. Development 140, 4386–4397 (2013). 24067355 10.1242/dev.098046

[b7] Ruiz-Linares A. *et al.* Admixture in latin america: geographic structure, phenotypic diversity and self-perception of ancestry based on 7,342 individuals. PLoS Genet. 10, e1004572 (2014). 25254375 10.1371/journal.pgen.1004572 PMC4177621

[b8] Speed D., Hemani G., Johnson M. R. & Balding D. J. Improved heritability estimation from genome-wide SNPs. Am. J. Hum. Genet. 91, 1011–1021 (2012). 23217325 10.1016/j.ajhg.2012.10.010 PMC3516604

[b9] Yang J., Lee S. H., Goddard M. E. & Visscher P. M. GCTA: a tool for genome-wide complex trait analysis. Am. J. Hum. Genet. 88, 76–82 (2011). 21167468 10.1016/j.ajhg.2010.11.011 PMC3014363

[b10] Carson E. A. Maximum likelihood estimation of human craniometric heritabilities. Am. J. Phys. Anthropol. 131, 169–180 (2006). 16552732 10.1002/ajpa.20424

[b11] Martinez-Abadias N. *et al.* Heritability of human cranial dimensions: comparing the evolvability of different cranial regions. J. Anat. 214, 19–35 (2009). 19166470 10.1111/j.1469-7580.2008.01015.x PMC2667914

[b12] Purcell S. *et al.* PLINK: a tool set for whole-genome association and population-based linkage analyses. Am. J. Hum. Genet. 81, 559–575 (2007). 17701901 10.1086/519795 PMC1950838

[b13] Lippert C. *et al.* FaST linear mixed models for genome-wide association studies. Nat. Methods 8, 833–835 (2011). 21892150 10.1038/nmeth.1681

[b14] O'Reilly P. F. *et al.* MultiPhen: joint model of multiple phenotypes can increase discovery in GWAS. PLoS ONE 7, e34861 (2012). 22567092 10.1371/journal.pone.0034861 PMC3342314

[b15] Willer C. J., Li Y. & Abecasis G. R. METAL: fast and efficient meta-analysis of genomewide association scans. Bioinformatics 26, 2190–2191 (2010). 20616382 10.1093/bioinformatics/btq340 PMC2922887

[b16] Headon D. J. *et al.* Gene defect in ectodermal dysplasia implicates a death domain adapter in development. Nature 414, 913–916 (2001). 11780064 10.1038/414913a

[b17] Mou C. *et al.* Enhanced ectodysplasin-A receptor (EDAR) signaling alters multiple fiber characteristics to produce the East Asian hair form. Hum. Mutat. 29, 1405–1411 (2008). 18561327 10.1002/humu.20795

[b18] Bryk J. *et al.* Positive selection in East Asians for an EDAR allele that enhances NF-kappaB activation. PloS ONE. 3, e2209 (2008). 18493316 10.1371/journal.pone.0002209 PMC2374902

[b19] Mikkola M. L. Molecular aspects of hypohidrotic ectodermal dysplasia. Am. J. Med. Genet. A 149 A, 2031–2036 (2009). 19681132 10.1002/ajmg.a.32855

[b20] Fujimoto A. *et al.* A scan for genetic determinants of human hair morphology: EDAR is associated with Asian hair thickness. Hum. Mol. Genet. 17, 835–843 (2008). 18065779 10.1093/hmg/ddm355

[b21] Fujimoto A. *et al.* A replication study confirmed the EDAR gene to be a major contributor to population differentiation regarding head hair thickness in Asia. Hum. Genet. 124, 179–185 (2008). 18704500 10.1007/s00439-008-0537-1

[b22] Kimura R. *et al.* A common variation in EDAR is a genetic determinant of shovel-shaped incisors. Am. J. Hum. Genet. 85, 528–535 (2009). 19804850 10.1016/j.ajhg.2009.09.006 PMC2756549

[b23] Park J. H. *et al.* Effects of an Asian-specific nonsynonymous EDAR variant on multiple dental traits. J. Hum. Genet. 57, 508–514 (2012). 22648185 10.1038/jhg.2012.60

[b24] Tan J. *et al.* The adaptive variant EDARV370A is associated with straight hair in East Asians. Hum. Genet. 132, 1187–1191 (2013). 23793515 10.1007/s00439-013-1324-1

[b25] Kamberov Y. G. *et al.* Modeling recent human evolution in mice by expression of a selected EDAR variant. Cell 152, 691–702 (2013). 23415220 10.1016/j.cell.2013.01.016 PMC3575602

[b26] Monreal A. W. *et al.* Mutations in the human homologue of mouse dl cause autosomal recessive and dominant hypohidrotic ectodermal dysplasia. Nat. Genet. 22, 366–369 (1999). 10431241 10.1038/11937

[b27] Singh M. K. *et al.* The T-box transcription factor Tbx15 is required for skeletal development. Mech. Dev. 122, 131–144 (2005). 15652702 10.1016/j.mod.2004.10.011

[b28] Curry G. A. Genetical and development studies on droopy-eared mice. J. Embryol. Exp. Morphol. 7, 39–65 (1959). 13654621

[b29] Candille S. I. *et al.* Dorsoventral patterning of the mouse coat by Tbx15. PLoS Biol. 2, E3 (2004). 14737183 10.1371/journal.pbio.0020003 PMC314463

[b30] Lausch E. *et al.* TBX15 mutations cause craniofacial dysmorphism, hypoplasia of scapula and pelvis, and short stature in Cousin syndrome. Am J Hum Genet. 83, 649–655 (2008). 19068278 10.1016/j.ajhg.2008.10.011 PMC2668032

[b31] Qu S., Tucker S. C., Zhao Q., deCrombrugghe B. & Wisdom R. Physical and genetic interactions between Alx4 and Cart1. Development 126, 359–369 (1999). 9847249 10.1242/dev.126.2.359

[b32] Nakashima M. *et al.* A genome-wide association study identifies four susceptibility loci for keloid in the Japanese population. Nat. Genet. 42, 768–771 (2010). 20711176 10.1038/ng.645

[b33] Smits P. *et al.* Mutation in mitochondrial ribosomal protein MRPS22 leads to Cornelia de Lange-like phenotype, brain abnormalities and hypertrophic cardiomyopathy. Eur. J. Hum. Genet. 19, 394–399 (2011). 21189481 10.1038/ejhg.2010.214 PMC3060326

[b34] Beysen D. *et al.* Deletions involving long-range conserved nongenic sequences upstream and downstream of FOXL2 as a novel disease-causing mechanism in blepharophimosis syndrome. Am. J. Hum. Genet. 77, 205–218 (2005). 15962237 10.1086/432083 PMC1224524

[b35] D'Haene B. *et al.* Disease-causing 7.4 kb cis-regulatory deletion disrupting conserved non-coding sequences and their interaction with the FOXL2 promotor: implications for mutation screening. PLoS Genet. 5, e1000522 (2009). 19543368 10.1371/journal.pgen.1000522 PMC2689649

[b36] Rimoin D. L. & Emery A. E. H. Emery and Rimoin's Principles and Practice of Medical Genetics 5th edn Churchill Livingstone (2007).

[b37] Weidinger G., Thorpe C. J., Wuennenberg-Stapleton K., Ngai J. & Moon R. T. The Sp1-related transcription factors sp5 and sp5-like act downstream of Wnt/beta-catenin signaling in mesoderm and neuroectoderm patterning. Curr. Biol. 15, 489–500 (2005). 15797017 10.1016/j.cub.2005.01.041

[b38] Dunty W. C. Jr., Kennedy M. W., Chalamalasetty R. B., Campbell K. & Yamaguchi T. P. Transcriptional profiling of Wnt3a mutants identifies Sp transcription factors as essential effectors of the Wnt/beta-catenin pathway in neuromesodermal stem cells. PLoS ONE 9, e87018 (2014). 24475213 10.1371/journal.pone.0087018 PMC3901714

[b39] Lettre G. *et al.* Identification of ten loci associated with height highlights new biological pathways in human growth. Nat. Genet. 40, 584–591 (2008). 18391950 10.1038/ng.125 PMC2687076

[b40] Gudbjartsson D. F. *et al.* Many sequence variants affecting diversity of adult human height. Nat. Genet. 40, 609–615 (2008). 18391951 10.1038/ng.122

[b41] Soranzo N. *et al.* Meta-analysis of genome-wide scans for human adult stature identifies novel Loci and associations with measures of skeletal frame size. PLoS Genet. 5, e1000445 (2009). 19343178 10.1371/journal.pgen.1000445 PMC2661236

[b42] Coleman M. N. & Ross C. F. Primate auditory diversity and its influence on hearing performance. Anat. Rec. A Discov. Mol. Cell. Evol. Biol 281, 1123–1137 (2004). 15470672 10.1002/ar.a.20118

[b43] Coleman M. N. & Colbert M. W. Correlations between auditory structures and hearing sensitivity in non-human primates. J. Morphol. 271, 511–532 (2010). 20025067 10.1002/jmor.10814

[b44] Lieberman D. The Evolution of the Human Head Belknap Press of Harvard Univ. Press (2011).

[b45] Abaza A., Ross A., Herbert C., Harrison M. A. F. & Nixon M. S. A survey on ear biometrics. ACM Comput. Surv. 45, 1–35 (2013).

[b46] Junod S., Pasquier J. & Champod C. The development of an automatic recognition system for earmark and earprint comparisons. Forensic Sci. Int. 222, 170–178 (2012). 22840281 10.1016/j.forsciint.2012.05.021

[b47] The MathWorks,. Inc. MATLAB and Statistics Toolbox Release 2013b The MathWorks, Inc. (2013).

[b48] Shrout P. E. & Fleiss J. L. Intraclass correlations: uses in assessing rater reliability. Psychol. Bull. 86, 420–428 (1979). 18839484 10.1037//0033-2909.86.2.420

[b49] Alexander D. H., Novembre J. & Lange K. Fast model-based estimation of ancestry in unrelated individuals. Genome Res. 19, 1655–1664 (2009). 19648217 10.1101/gr.094052.109 PMC2752134

[b50] Dudoit S. & Laan M. J. V.D. Multiple Testing Procedures with Applications to Genomics Springer (2008).

[b51] Hastie T., Tibshirani R. & Friedman J. H. The Elements of Statistical Learning: Data Mining, Inference, and Prediction 2nd edn Springer (2009).

[b52] Zelditch M. L., Swiderski D. L., Sheets H. D. & Fink W. L. Geometric Morphometric for Biologists Elsevier (2004).

[b53] Klingenberg C. P. MorphoJ: an integrated software package for geometric morphometrics. Mol. Ecol. Resour. 11, 353–357 (2011). 21429143 10.1111/j.1755-0998.2010.02924.x

[b54] R. A Language and Environment for Statistical Computing R Foundation for Statistical Computing (2010).

[b55] Adobe Photoshop CS6 Adobe Systems Incorporated (2012).

[b56] Pruim R. J. *et al.* LocusZoom: regional visualization of genome-wide association scan results. Bioinformatics 26, 2336–2337 (2010). 20634204 10.1093/bioinformatics/btq419 PMC2935401

[b57] Barrett J. C., Fry B., Maller J. & Daly M. J. Haploview: analysis and visualization of LD and haplotype maps. Bioinformatics 21, 263–265 (2005). 15297300 10.1093/bioinformatics/bth457

