# Supplementary material for: A genome-wide association study identifies multiple loci for variation in human ear morphology
Source: Nat Commun. 2015 Jun 24;6:7500. doi: 10.1038/ncomms8500 (PMC4491814; doi:10.1038/ncomms8500)
Supplement: Supplementary Information — Supplementary Figures 1-11, Supplementary Tables 1-10, Supplementary Note 1 and Supplementary References [file ncomms8500-s1.pdf]

SUPPLEMENTARY FIGURES

Supplementary Figure 1: Categorical phenotyping protocol for the pinna traits examined

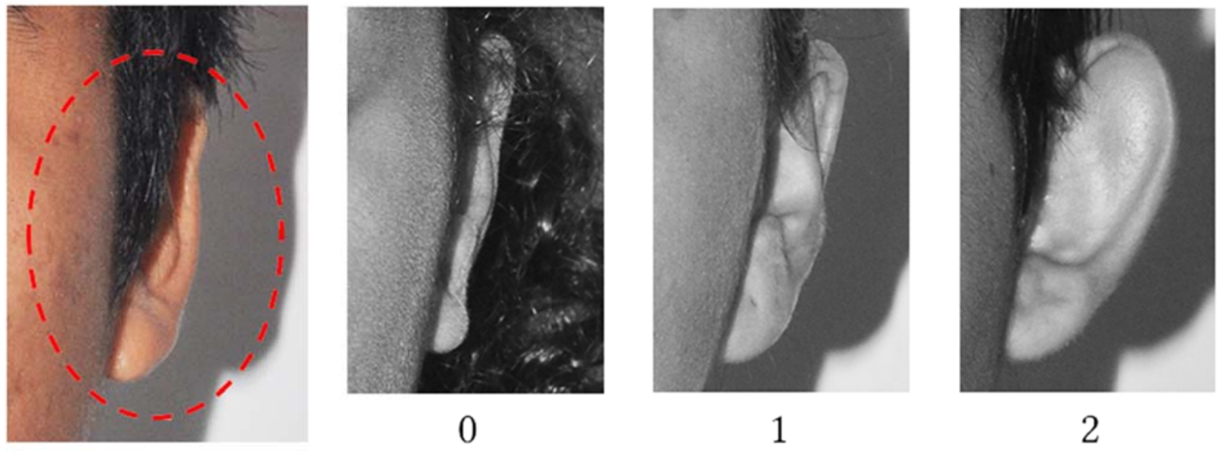

1. Ear Protrusion

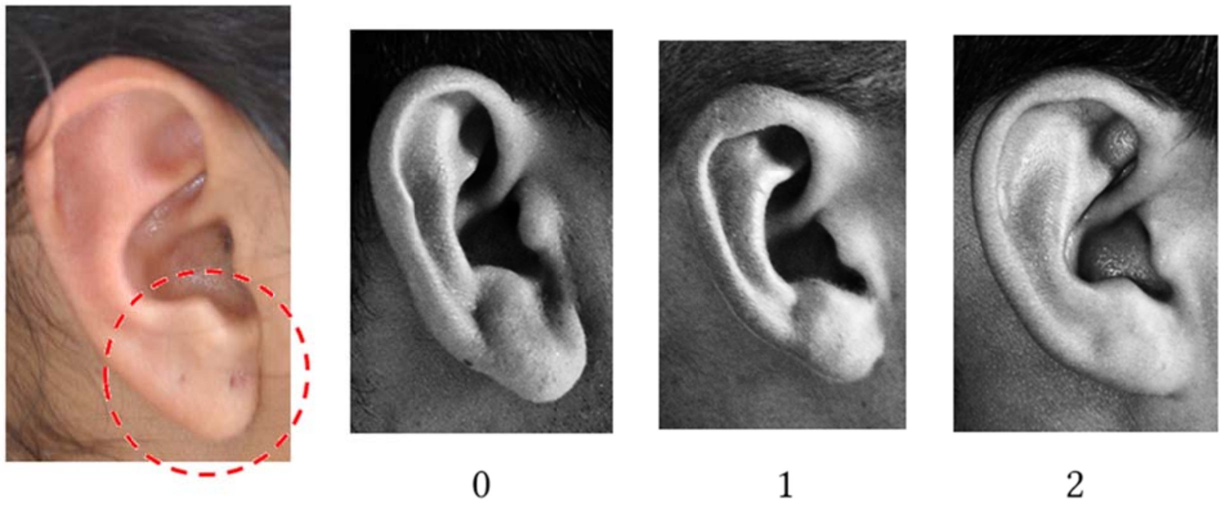

2. Lobe Attachment

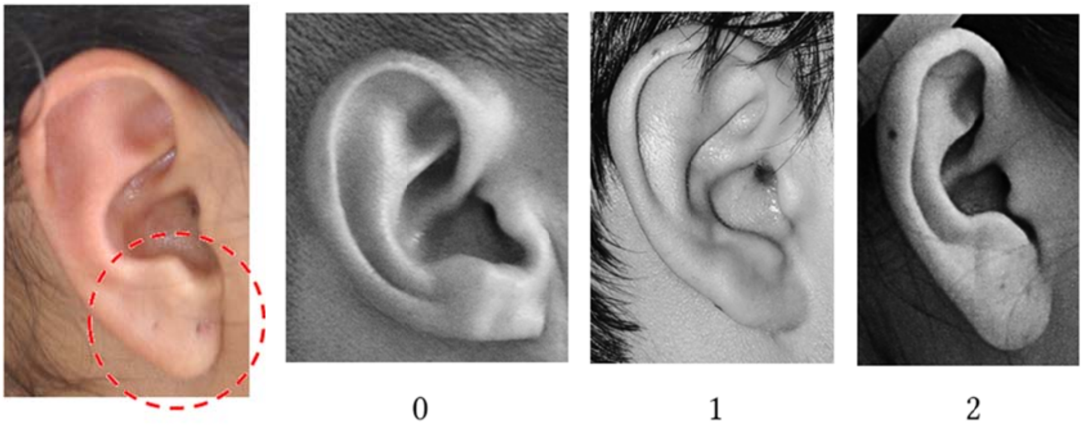

0 1 2

3. Lobe Size

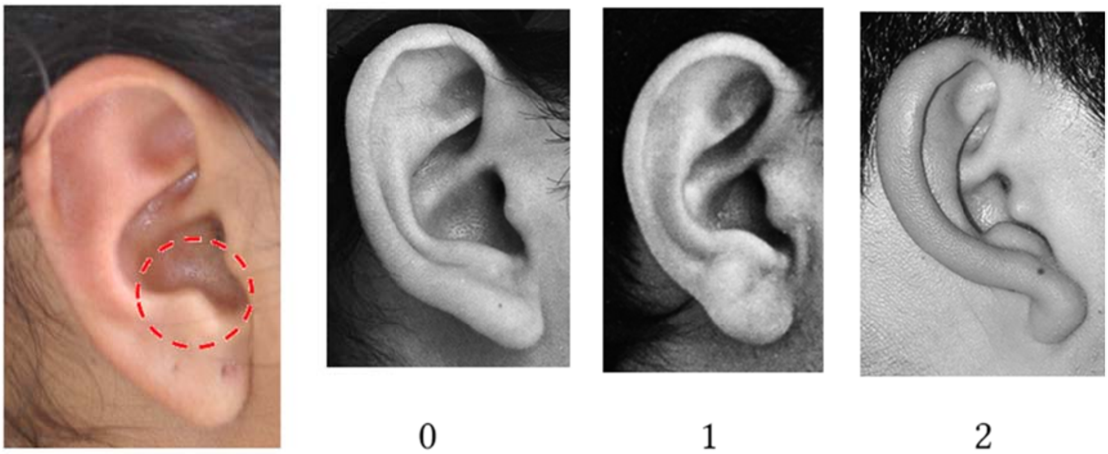

0 1 2

4. Antitragus Size

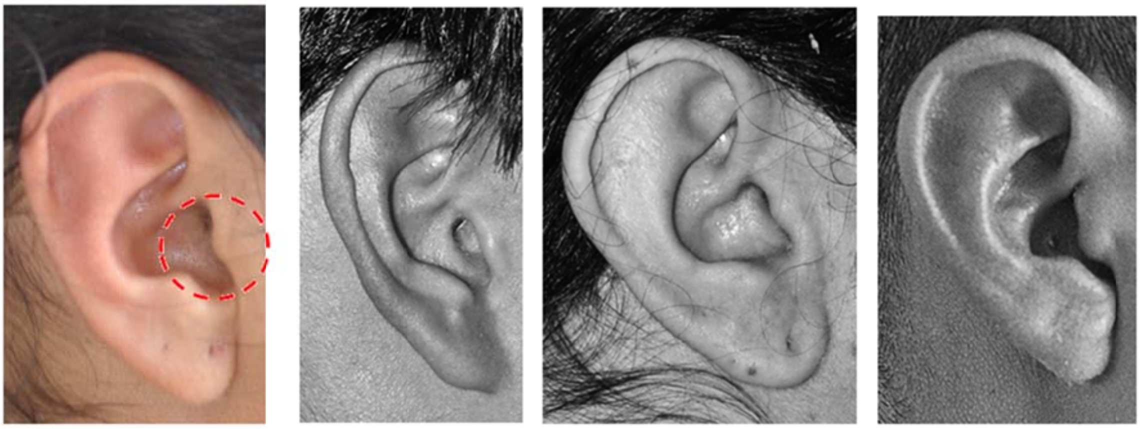

0 1 2

5. Tragus Size

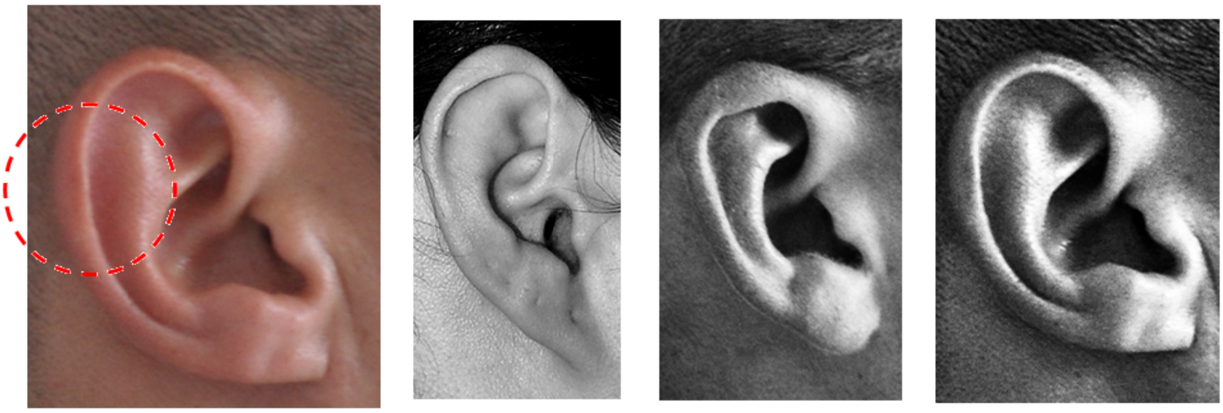

0 1 2

6. Helix Rolling

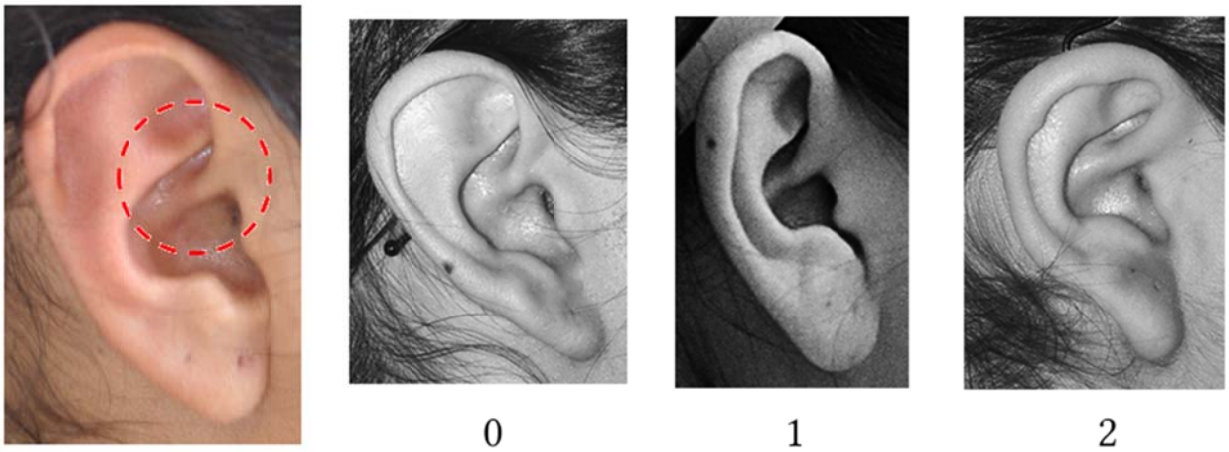

0 1 2

7. Crus Helix Expression

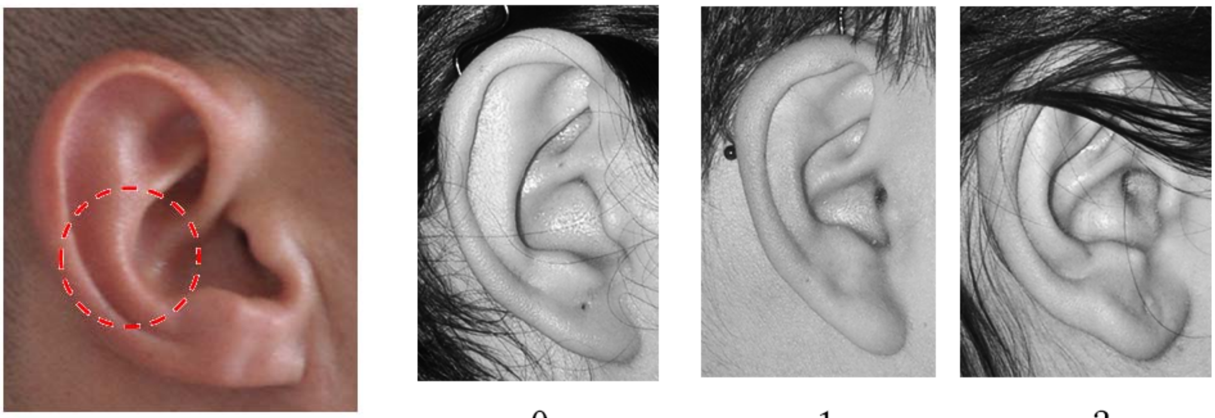

0 1 2

8. Folding of Antihelix

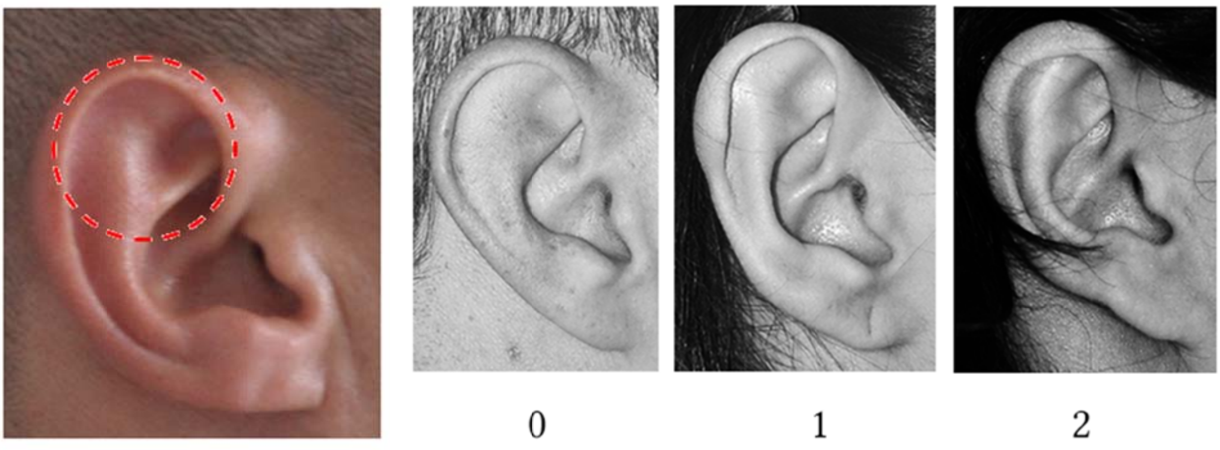

9. Superior Crus of Antihelix Expression

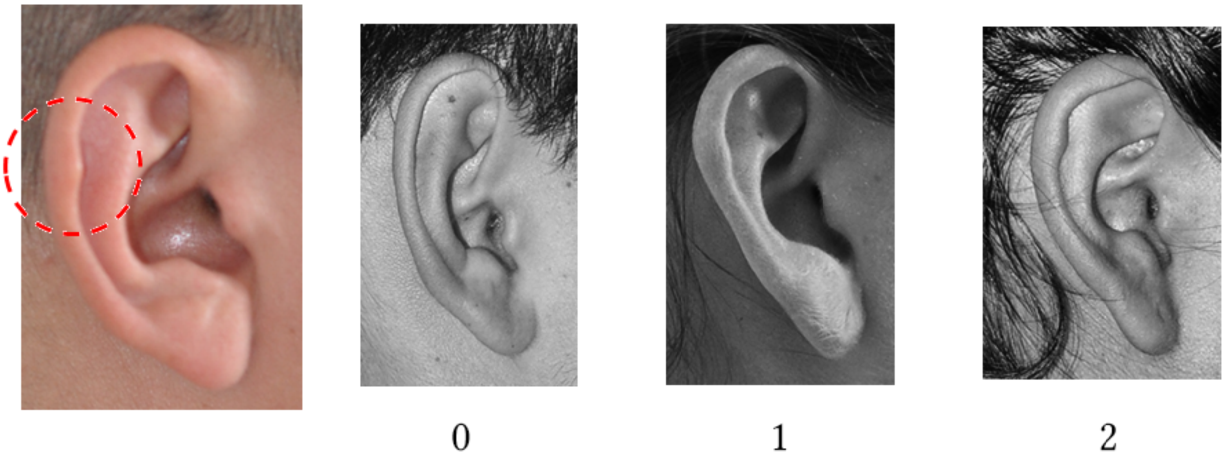

10. Darwin's Tubercle

Supplementary Figure 2: Frequency distribution of 10 pinna traits in the CANDELA sample

Ten pinna traits (Figure 1) were examined in 5,062 individuals and scored on a three point scale reflecting the degree of expression of the trait. The bar chart below shows the frequency (%) of the 3 categories for each trait examined in the CANDELA sample.

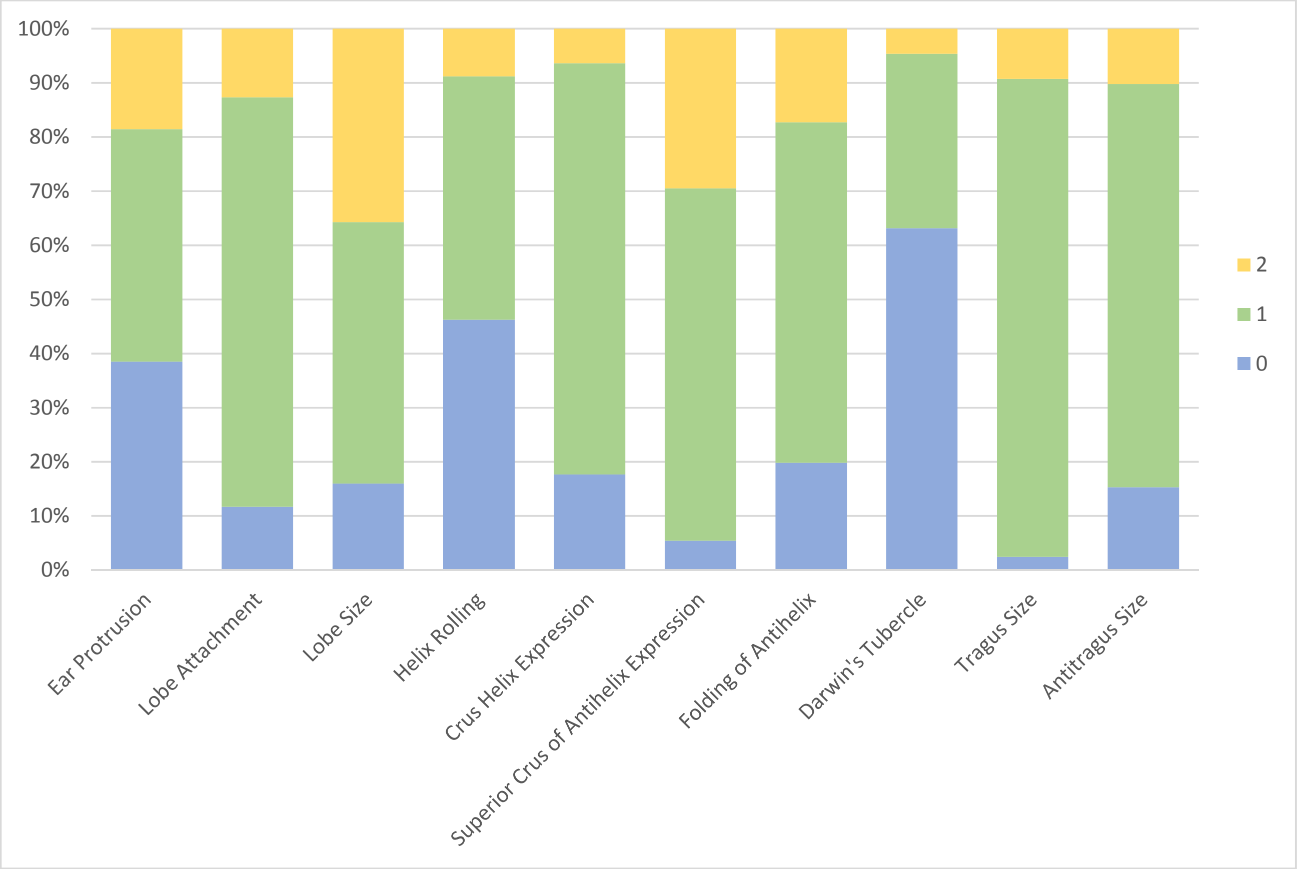

Supplementary Figure 3: Continental ancestry in the CANDELA sample

African, European and Native American ancestry values were estimated from a set of 93,328 autosomal SNPs (LD-pruned from the full chip data) via supervised runs of the Admixture software (Alexander et al. 2009). Reference populations for African and European groups were chosen from HAPMAP and from selected Native American populations as described in Ruiz-Linares et al (2014).

Individual ancestry barplots for each country are shown below. Individuals within each country are sorted by their American ancestry proportion.

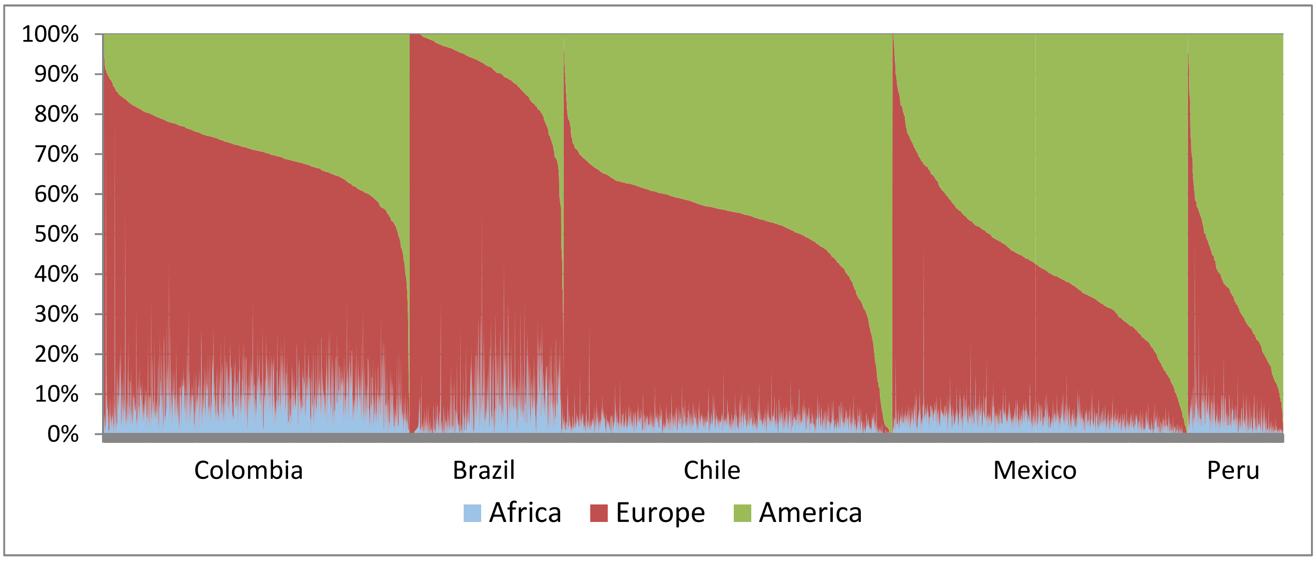

Mean ancestry estimates for each country and overall are:

	Africa	Europe	America
Colombia	7.8%	62.7%	29.5%
Brazil	6.1%	84.5%	9.4%
Chile	1.8%	49.5%	48.7%
Mexico	2.9%	38.2%	58.9%
Peru	3.0%	29.6%	67.4%
Overall	4.3%	53.0%	42.7%

Ref: Supplementary Reference 1 & 2.

Supplementary Figure 4: Selection of genetic Principal Components for inclusion in the GWAS analyses

A) Scree Plot:

Principal components were extracted from an LD-pruned SNP dataset (see methods). The proportion of the variance explained by each PC is shown below.

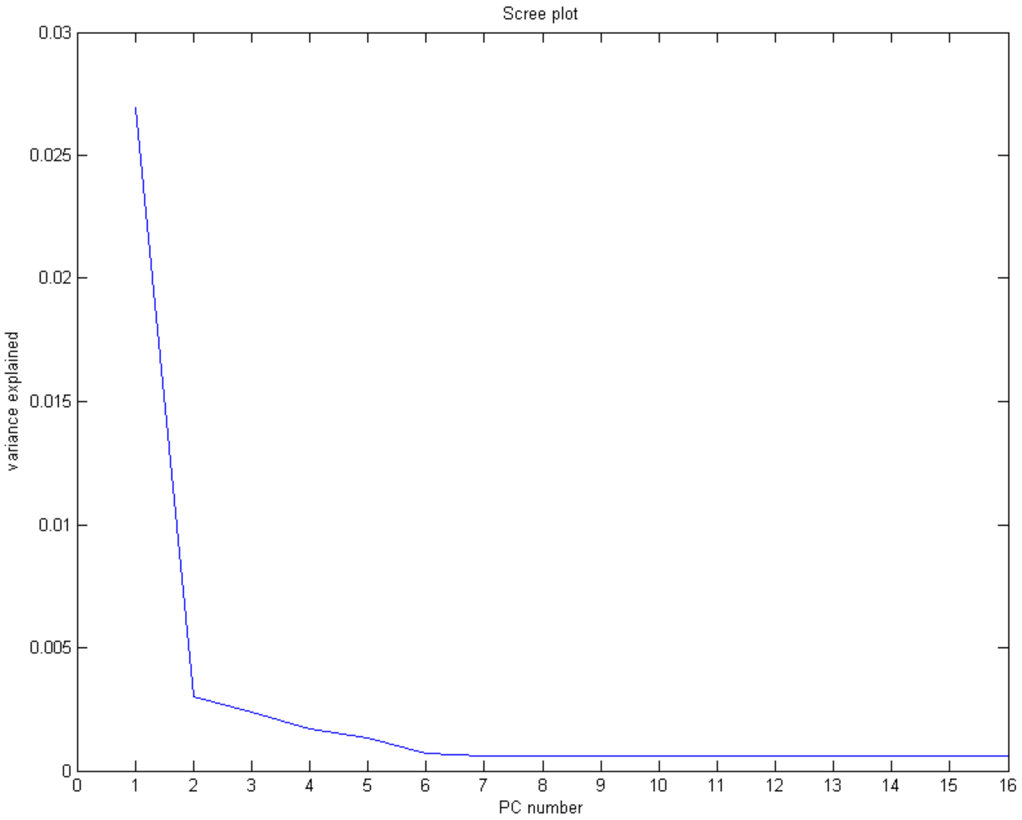

B) GWAS Q-Q plot without PC adjustment:

Q-Q plots were produced from the GWAS test statistics, without correction for population substructure through adjustment with principal components, to examine inflation of test statistics. There was indeed inflation for all traits, as the genomic control inflation factor lambda was > 2 in all cases. A sample Q-Q plot for Tragus Size is shown below.

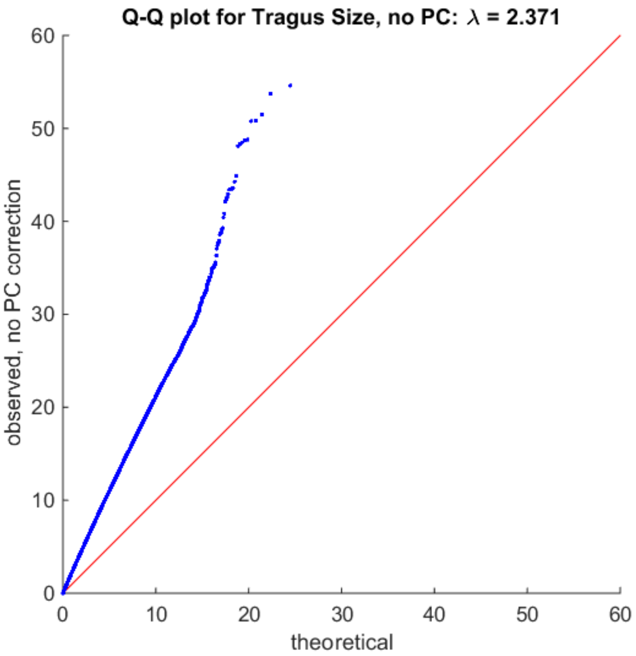

C) Manhattan plot for Tragus Size without PC adjustment:

GWAS Manhattan plot for Tragus Size is shown below. As expected from the strong inflation seen in Q-Q plot above, many SNPs are above significance level.

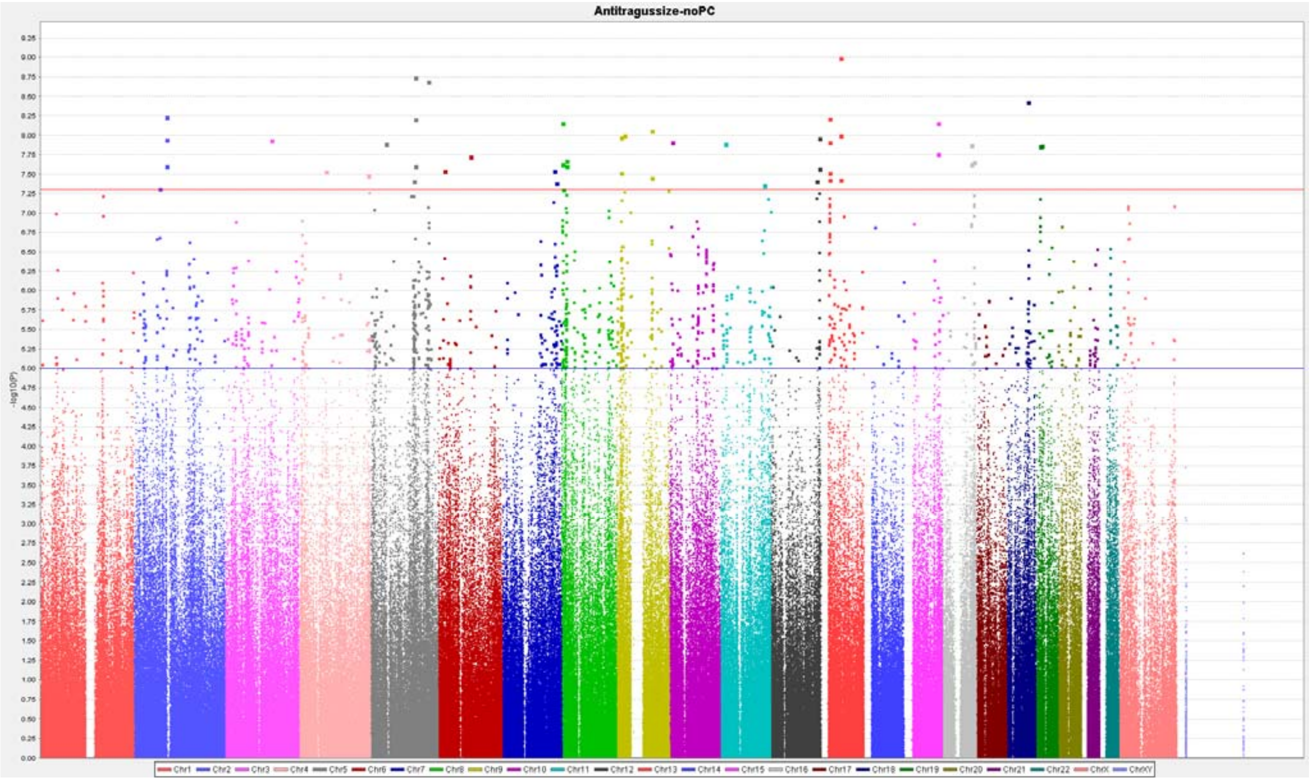

D) Manhattan plot for Tragus Size with genomic control adjustment:

GWAS test statistics obtained above were normalized by the genomic control adjustment method by diving with the inflation factor. Now all SNPs fell below significance level, as expected, since the adjustment factor was very high. This should be compared with the Manhattan plot for Tragus Size obtained after correction with 5 PCs, shown subsequently. It retains the ‘real’ association signal in chromosome 1, which is lost when a blanket correction on all SNPs is performed by genomic control. This demonstrates the superiority of the PC correction approach.

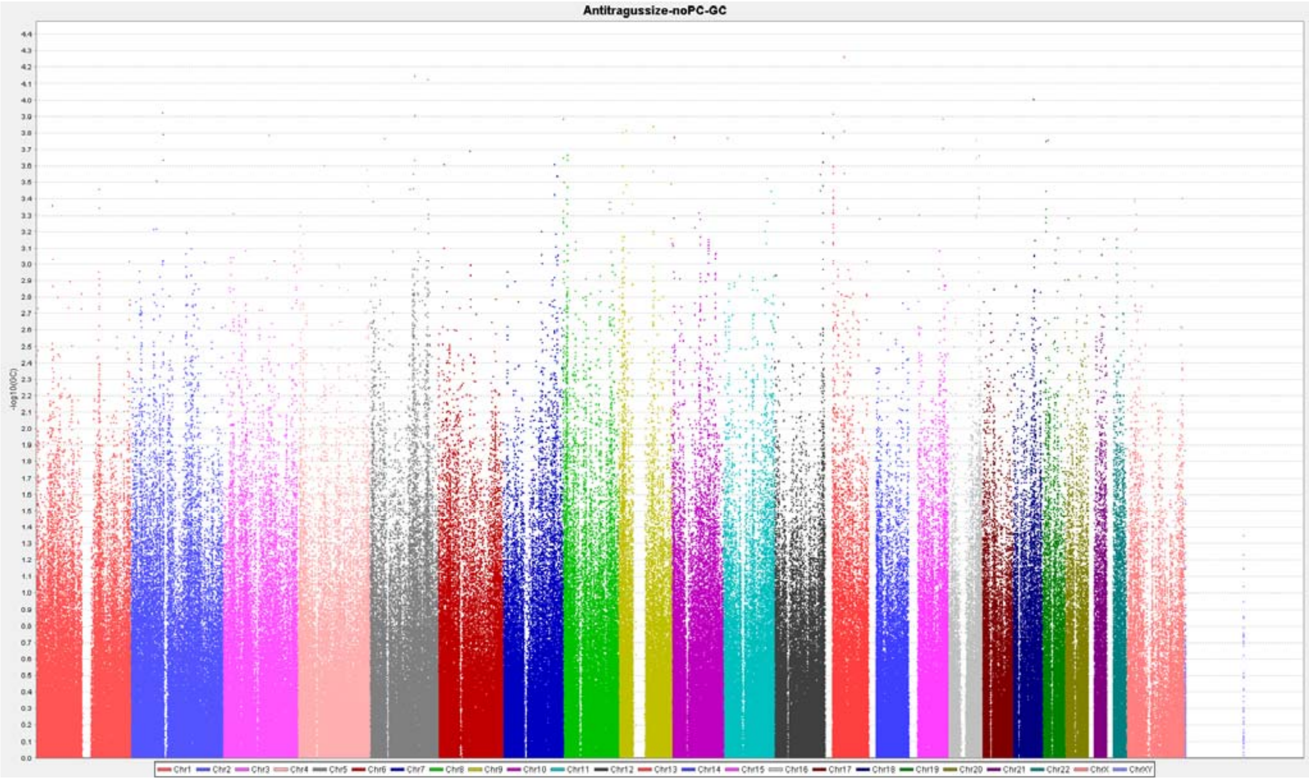

E) GWAS Q-Q plot with 5 PCs:

Q-Q plots were produced from the GWAS test statistics, to check if population substructure correction with 5 PCs is sufficient. Plots showed no noticeable deviation from expectation (a sample plot, for tragus size, is shown below). The genomic control inflation factor lambda was calculated in each case. All lambda values were < 1.03, indicating that correction with 5 PCs is sufficient.

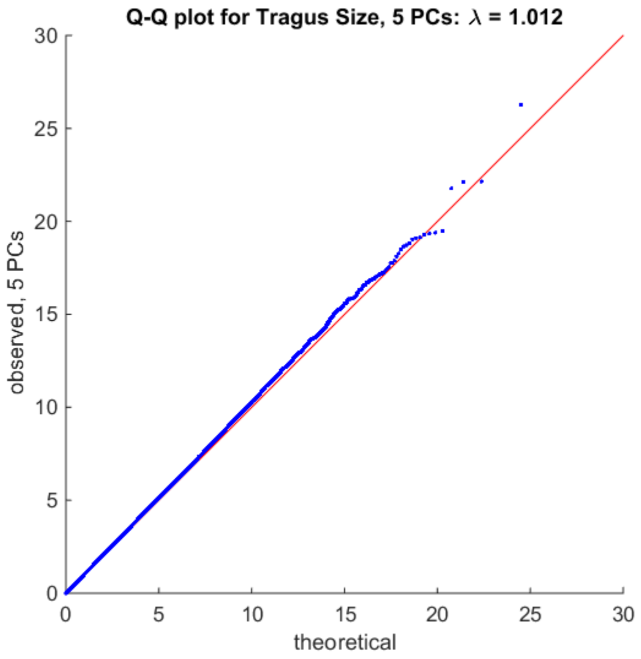

F) Manhattan plot for Tragus Size with genomic control adjustment:

This GWAS Manhattan plot, as discussed earlier, retains the strong association signal in chromosome 1, while removing the other spurious associations arising from population substructure.

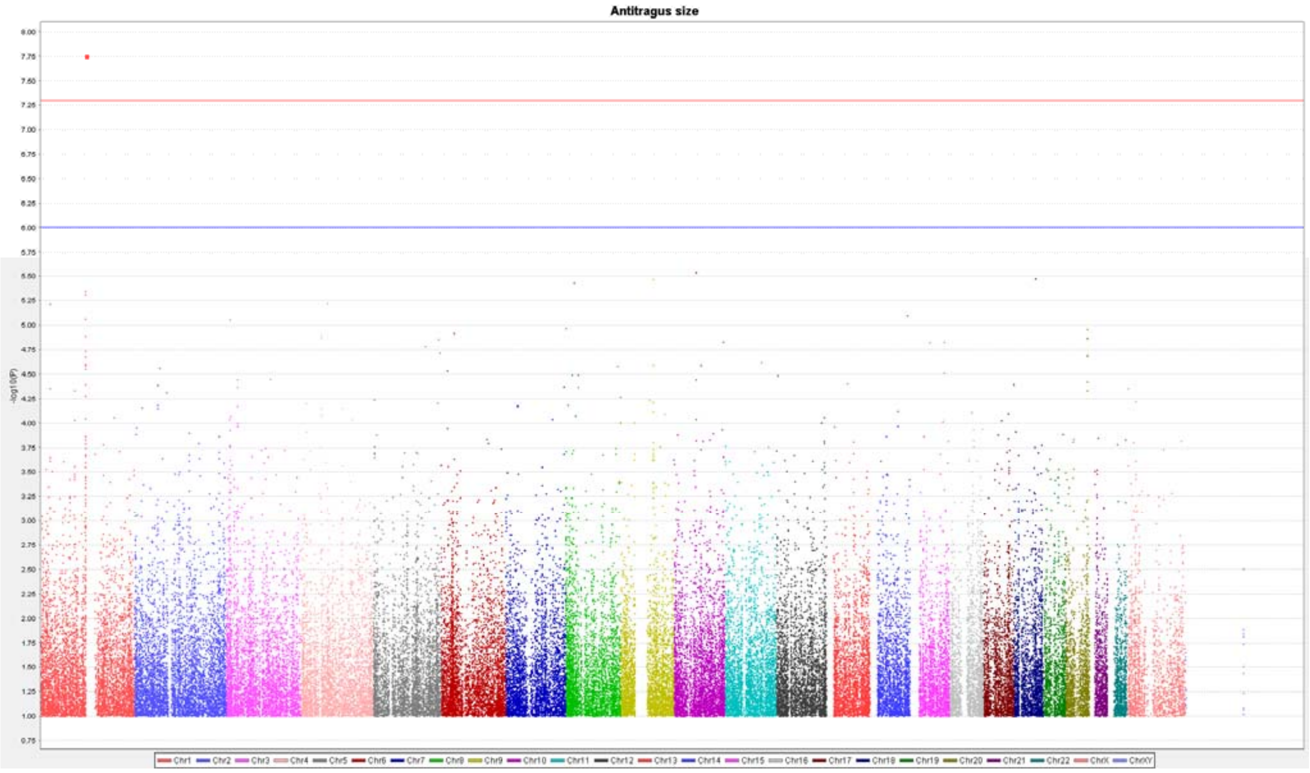

G) GWAS Q-Q plot with 10 PCs:

To ensure that inclusion of higher PCs does not have any noticeable effect, the GWASs were re-run with 10 PCs. There were no difference between the runs with 5 or 10 PCs; the ratios of lambda from both runs were very close to 1 for all traits. Below a sample Q-Q plot for tragus size is provided, comparing the test statistic values for runs with 5 and 10 PCs:

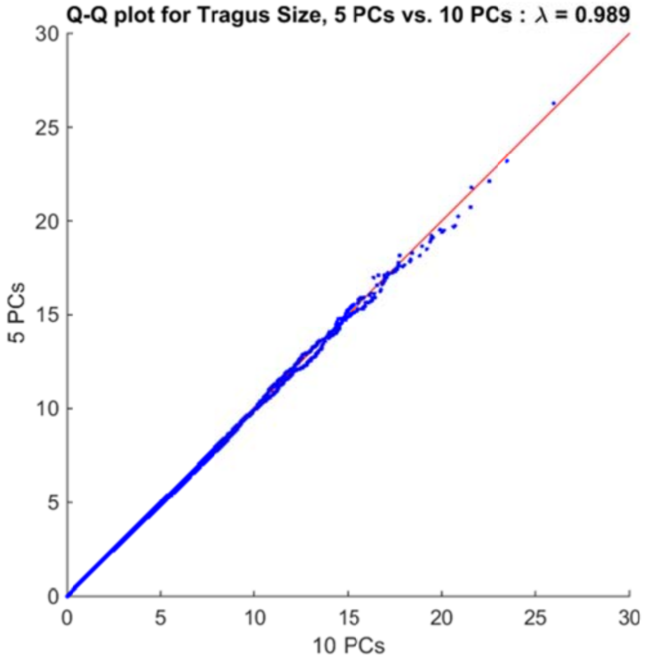

Supplementary Figure 5: GWAS combining the ten pinna traits in MultiPhen

Multivariate regression tests combining all pinna traits examined into a single model were carried out using MultiPhen (see methods). The Manhattan plot below summarizes the results obtained. The same set of regions showed genome-wide significant association as for the tests carried out for each phenotype independently.

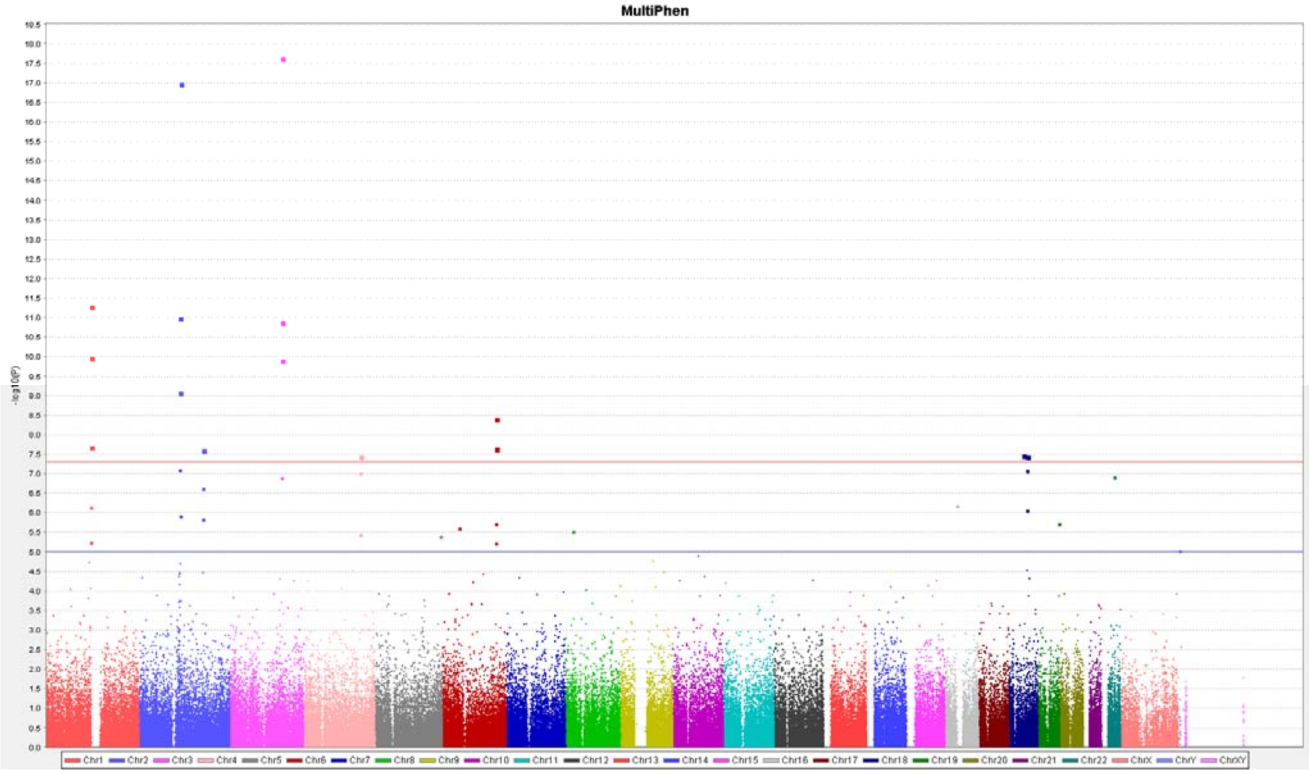

P-values obtained from MultiPhen (via a multivariate logistic regression) for index SNP in each associated region:

Region	SNP	P-value
1p12	rs17023457	5.01E-12
2q12.3	rs3827760	1.26E-11
2q31.1	rs2080401	2.51E-08
3q23	rs10212419	1.78E-11
4q31.3	rs1960918	4.17E-08
6q24.2	rs263156	4.90E-09
18q21.2	rs1619249	3.80E-08

Supplementary Figure 6: Meta-analysis Plots

A) Forest Plots for Country-stratified Meta-analysis

Forest plots are shown below for all SNPs and traits in Table 1. Meta-analysis p-values are presented in Supplementary Table 6.The coefficient value in each country is shown by a blue box marker (marker size being proportional to sample size for that country). The red markers indicate meta-analysis estimated effect size. Horizontal bars on each side of the markers indicate standard errors. Traits with genome-wide significant meta-analysis p-values are highlighted with a green box around the sub-figure.

rs17023457:

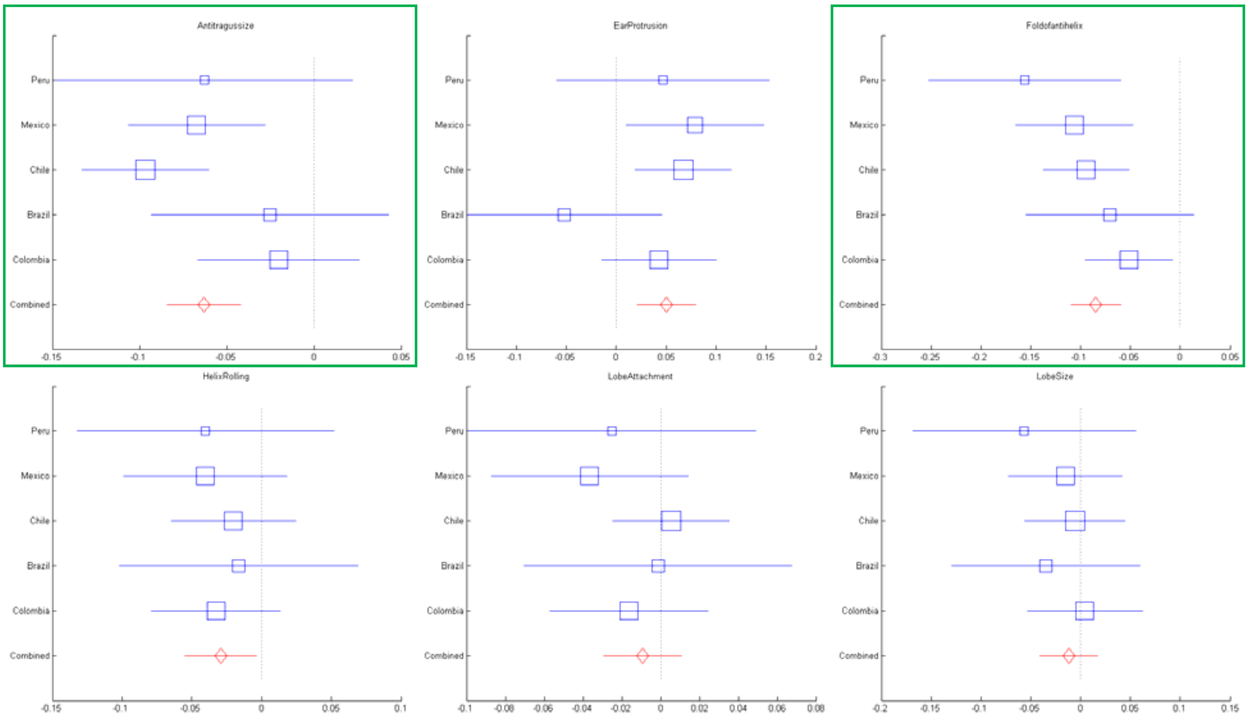

rs3827760:

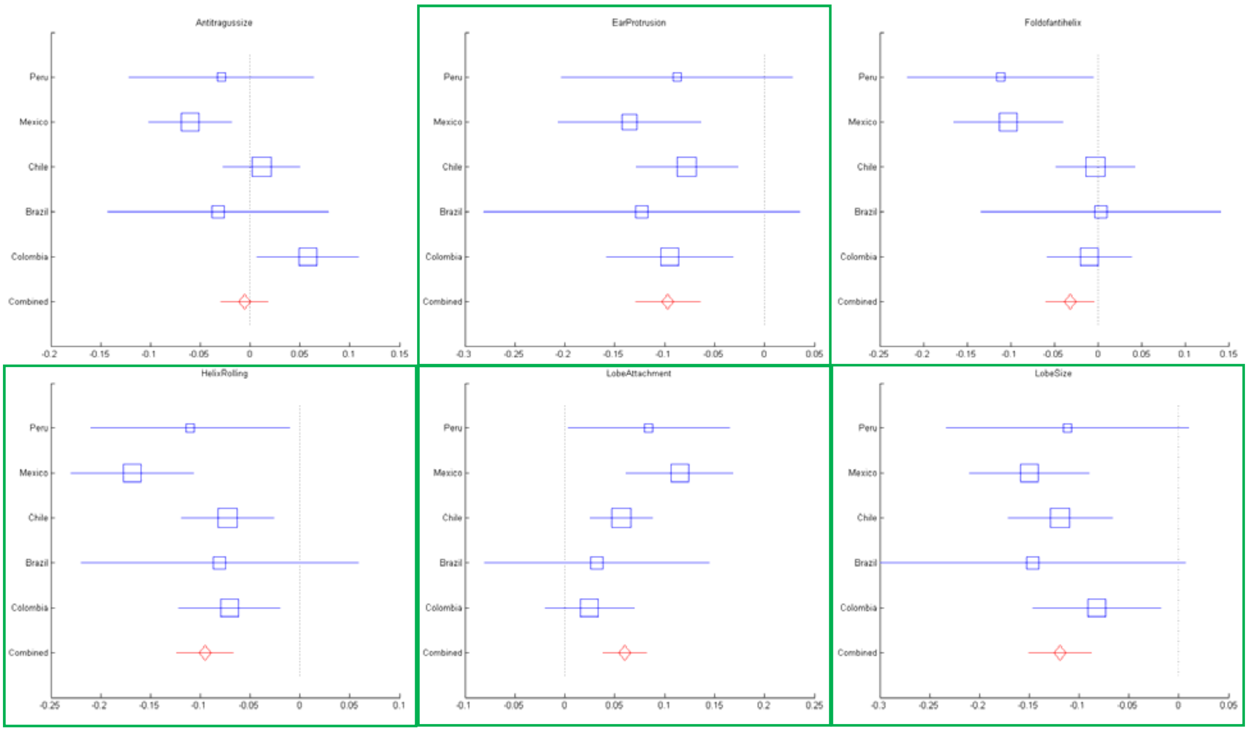

rs2080401:

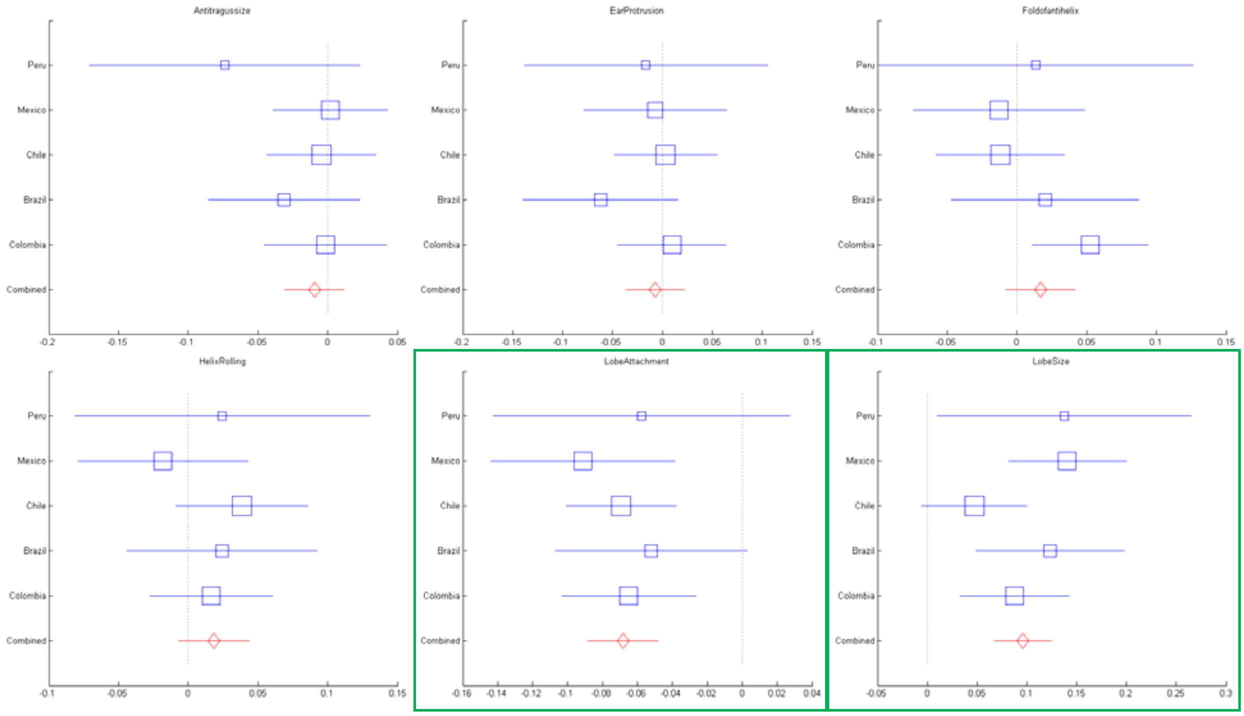

rs10212419:

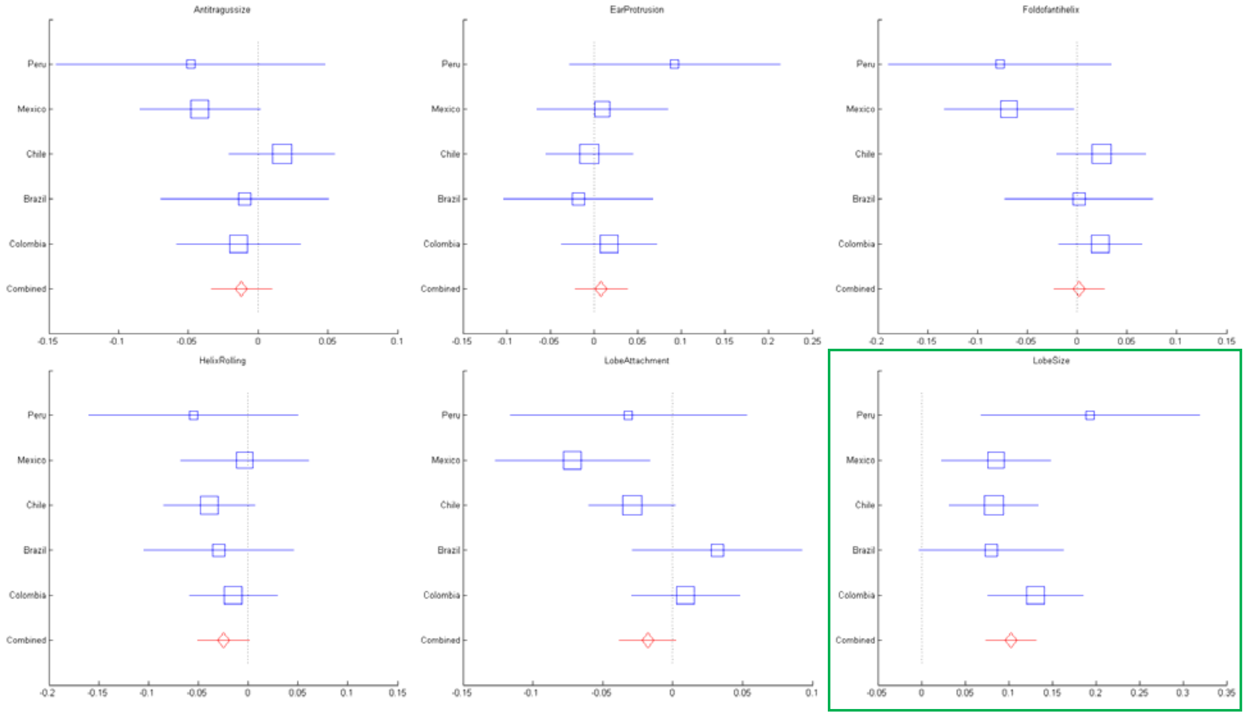

rs1960918:

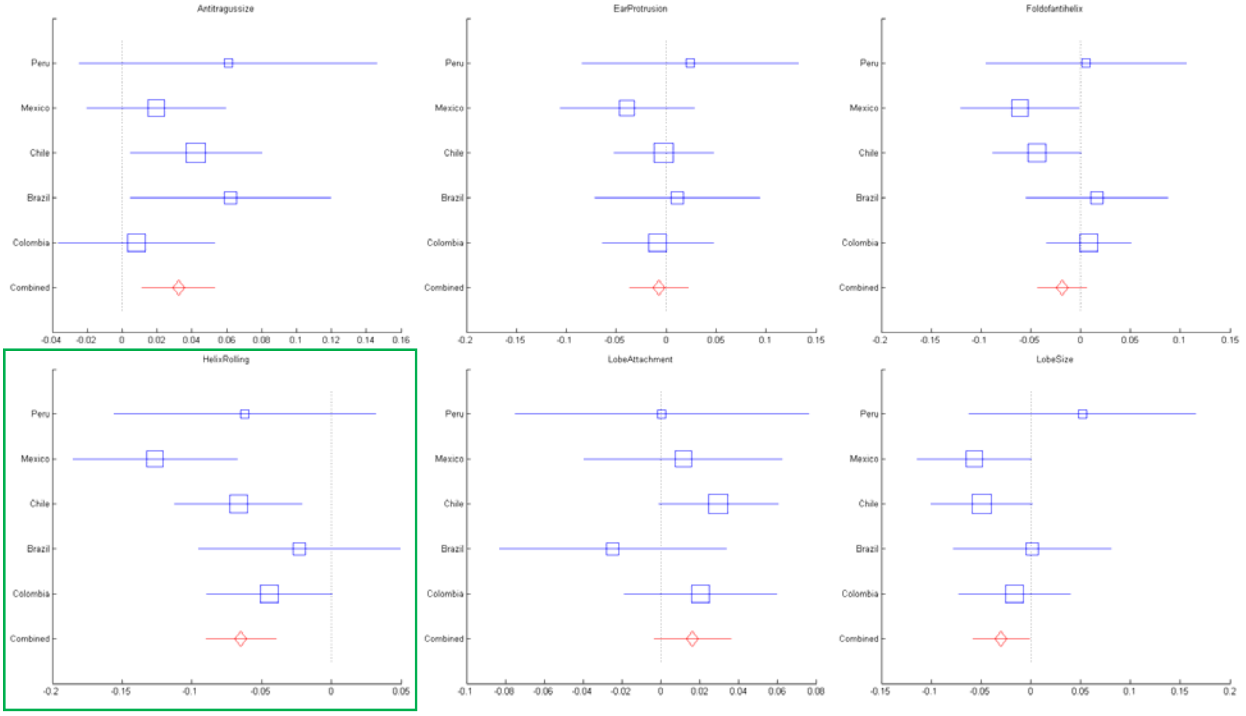

rs263156:

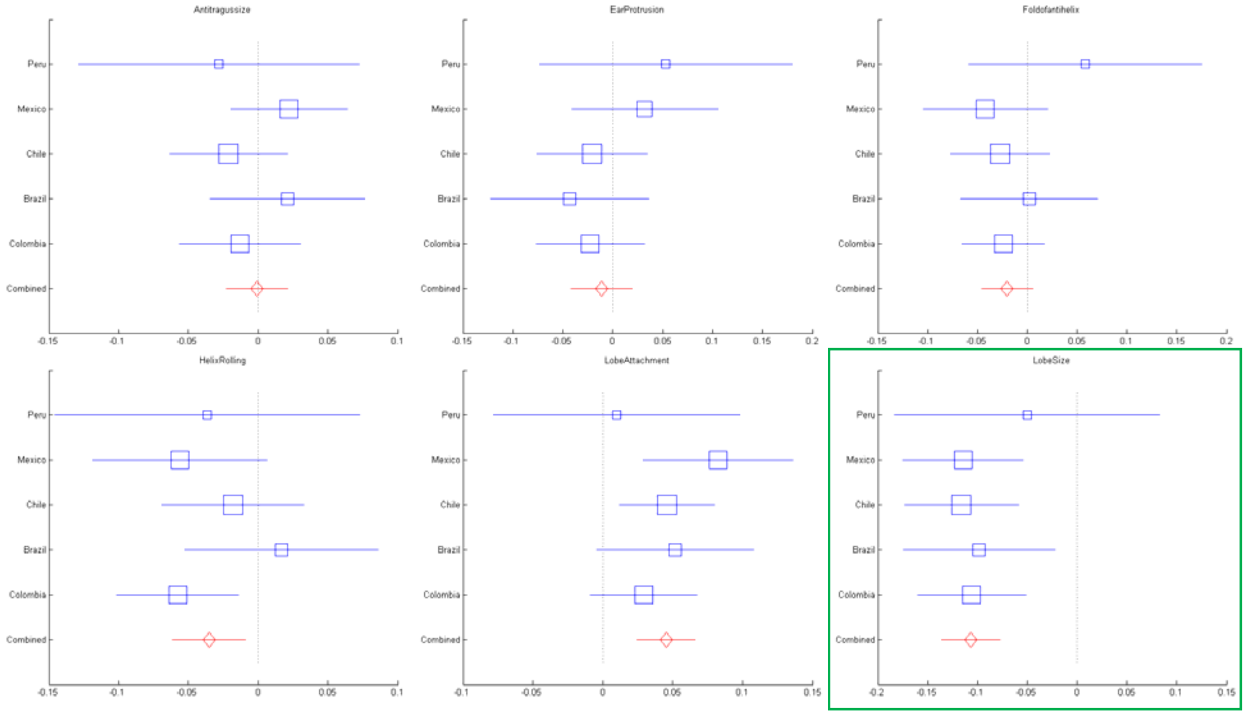

rs1619249:

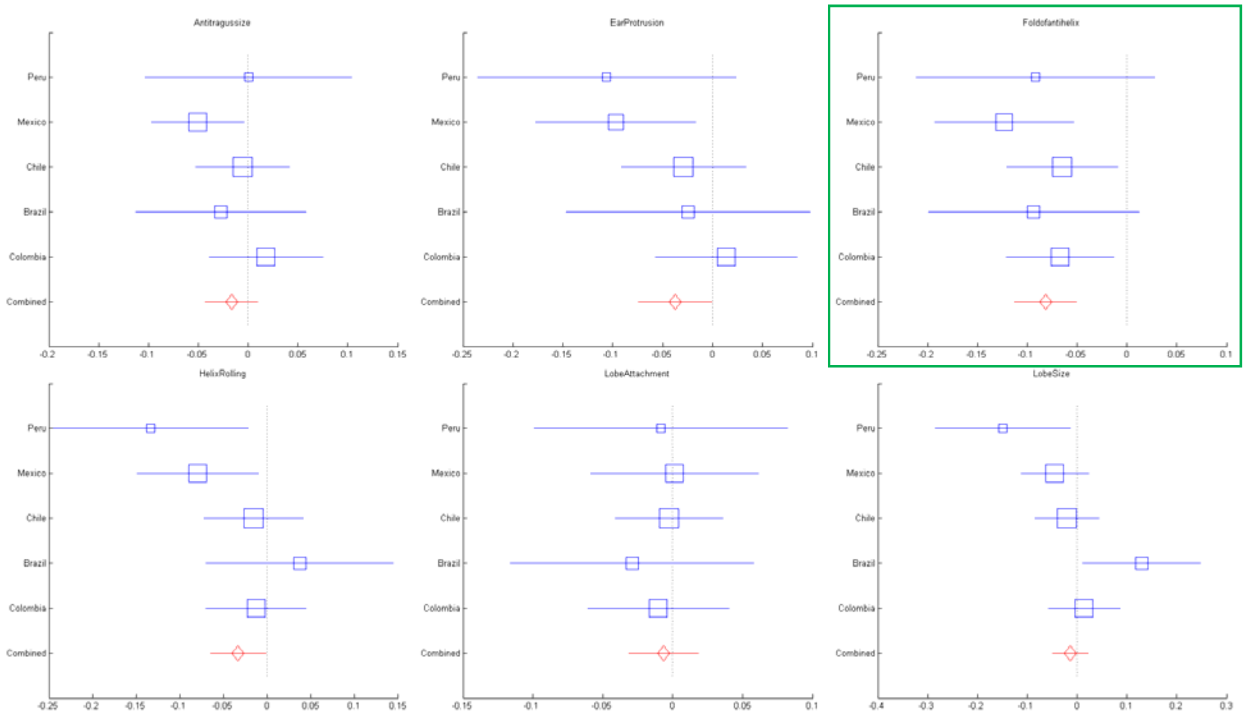

B) Manhattan Plots for Country- stratified Meta-analysis

Manhattan plots are shown below for all SNPs and traits in Table 1.

Ear Protrusion:

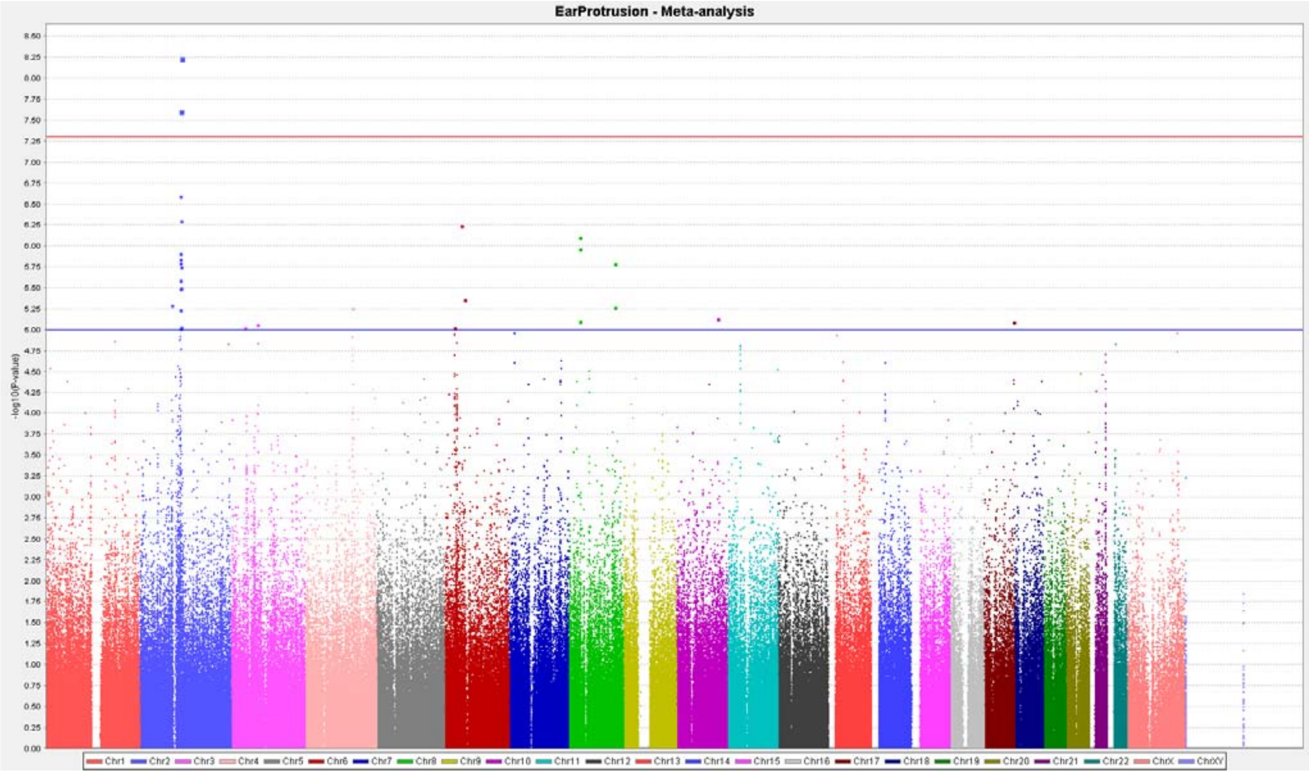

Antitragus Size:

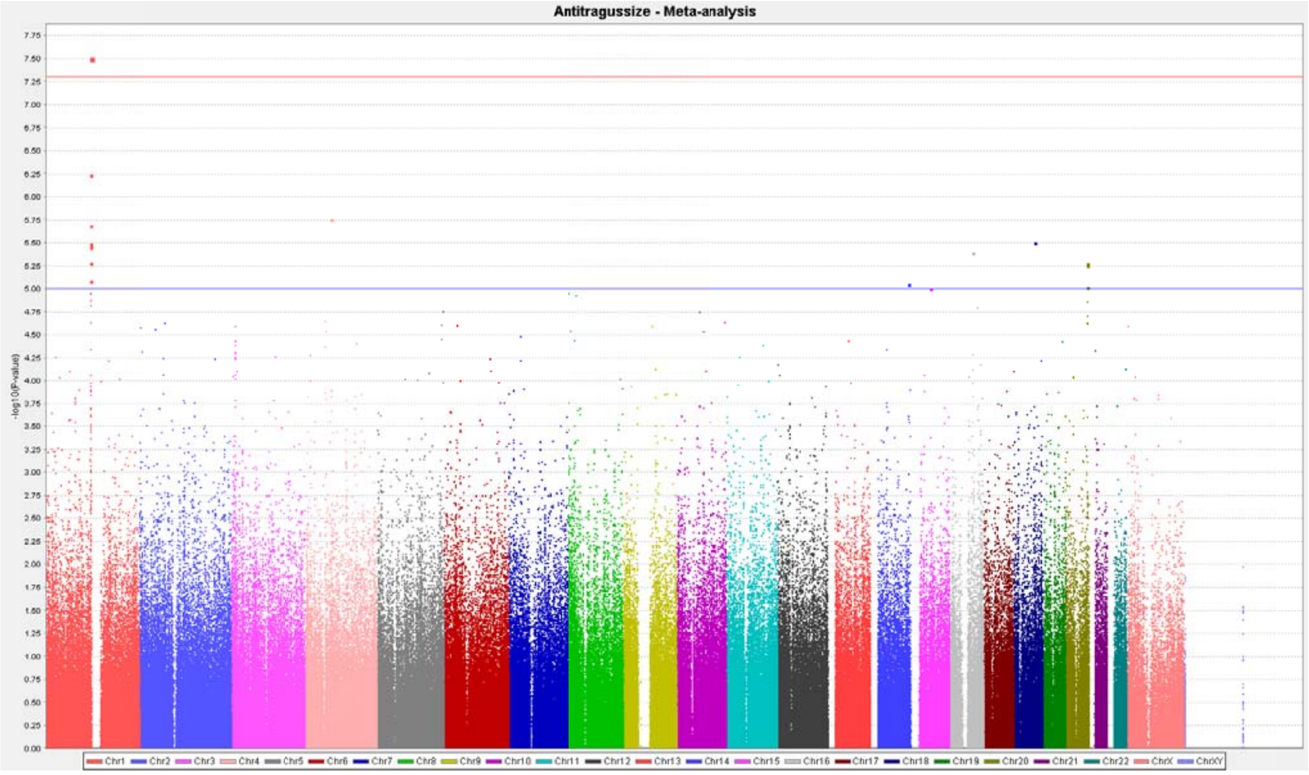

Folding of Antihelix:

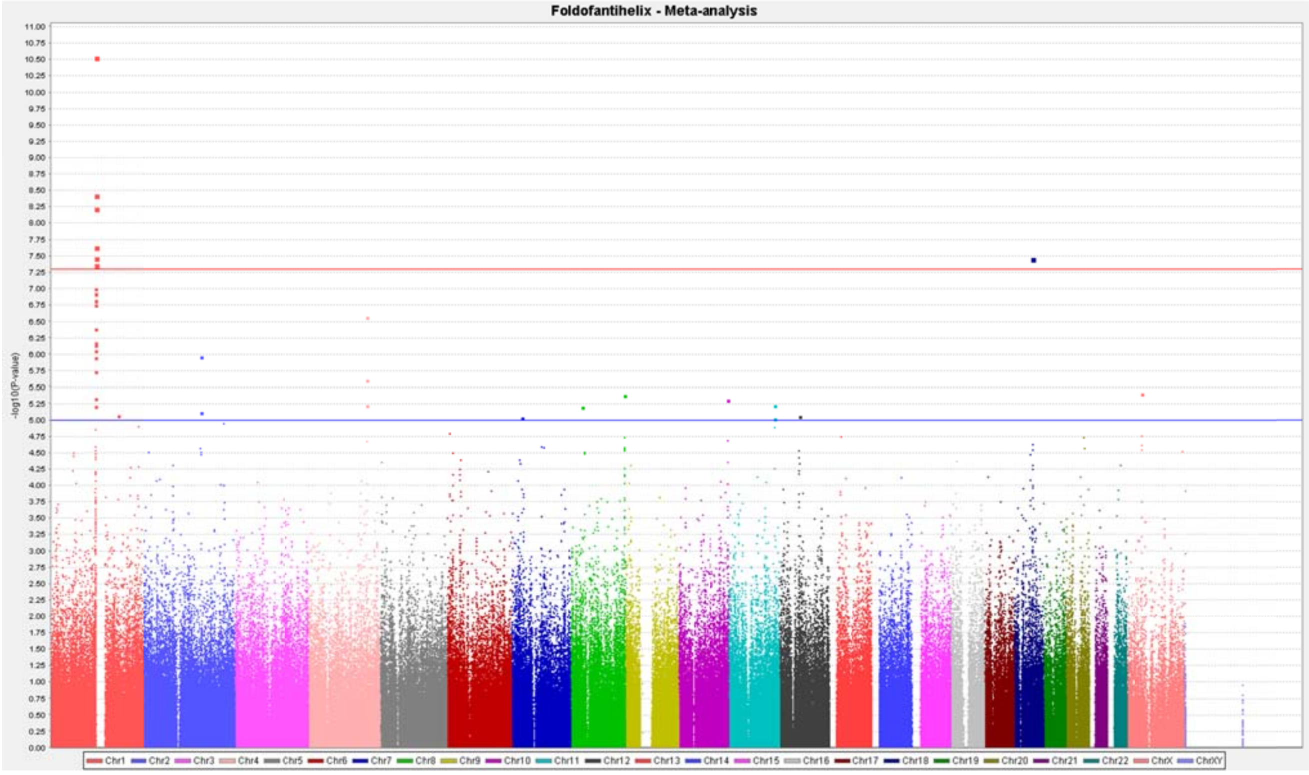

Helix Rolling:

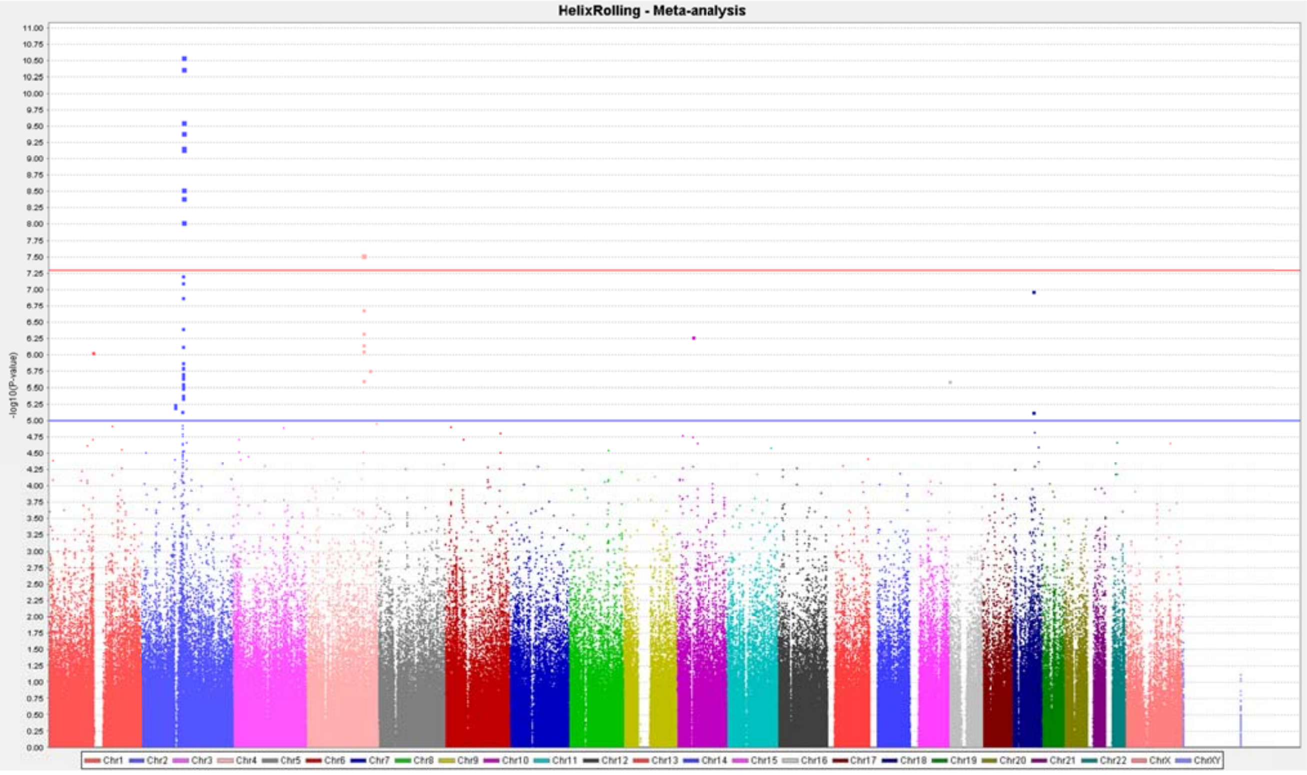

Lobe Attachment:

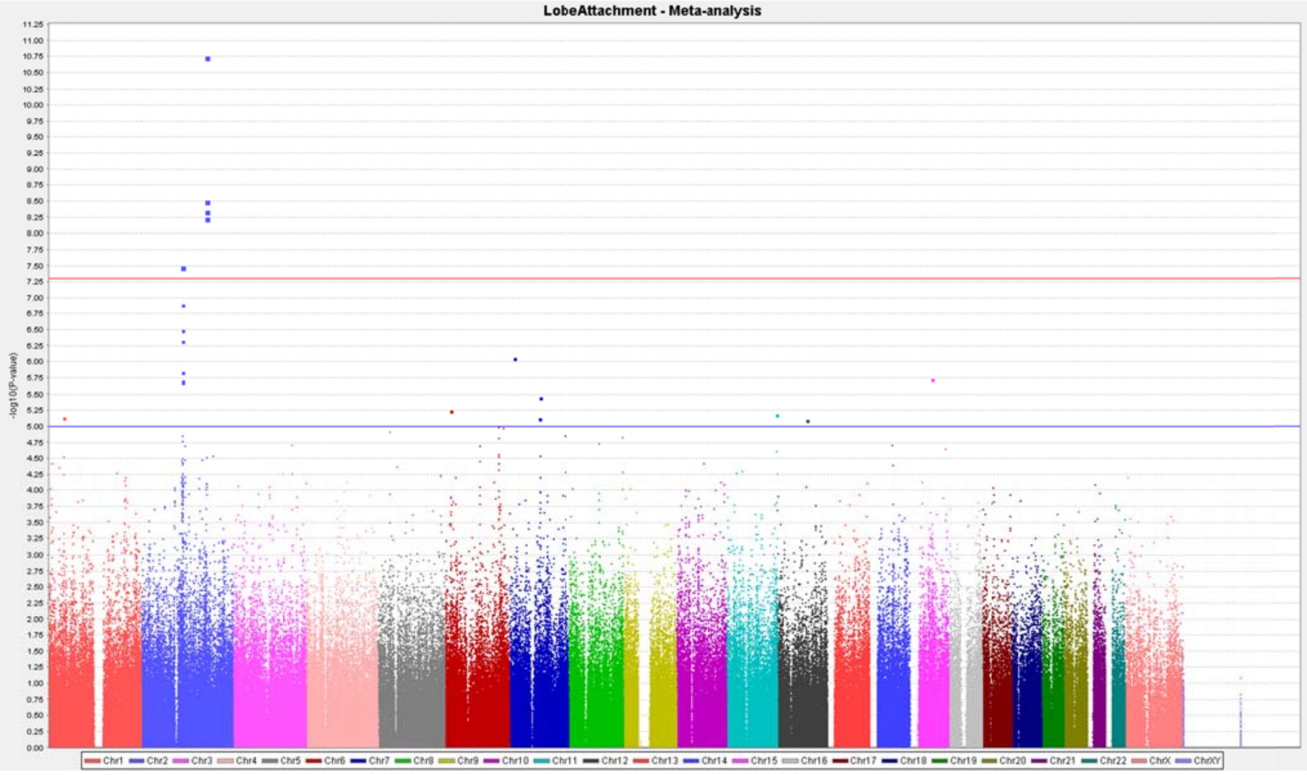

Lobe Size:

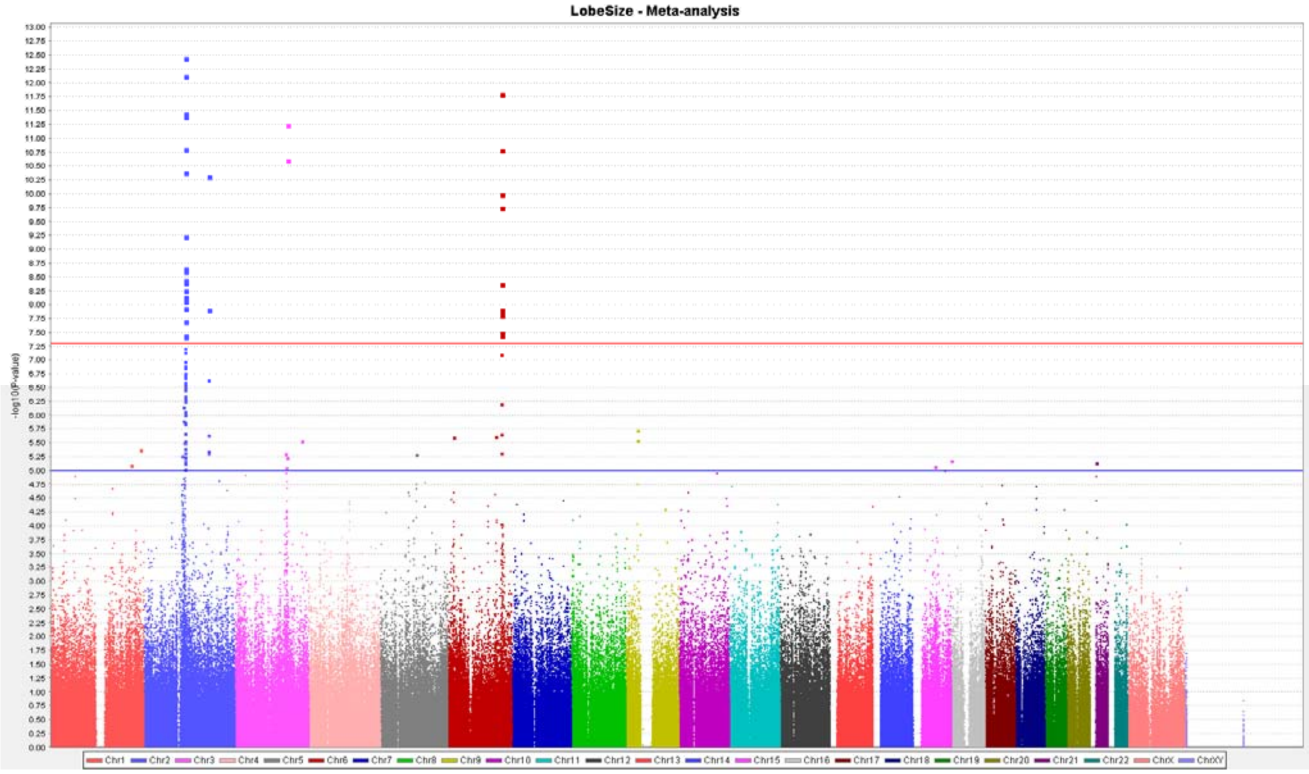
LobeSize - Meta-analysis
-log10(P-value)
Chr1 Chr2 Chr3 Chr4 Chr5 Chr6 Chr7 Chr8 Chr9 Chr10 Chr11 Chr12 Chr13 Chr14 Chr15 Chr16 Chr17 Chr18 Chr19 Chr20 Chr21 Chr22 ChrX ChrXY

C) Manhattan Plots for Sex- stratified Meta-analysis

Manhattan plots are shown below for all SNPs and traits in Table 1.

Ear Protrusion:

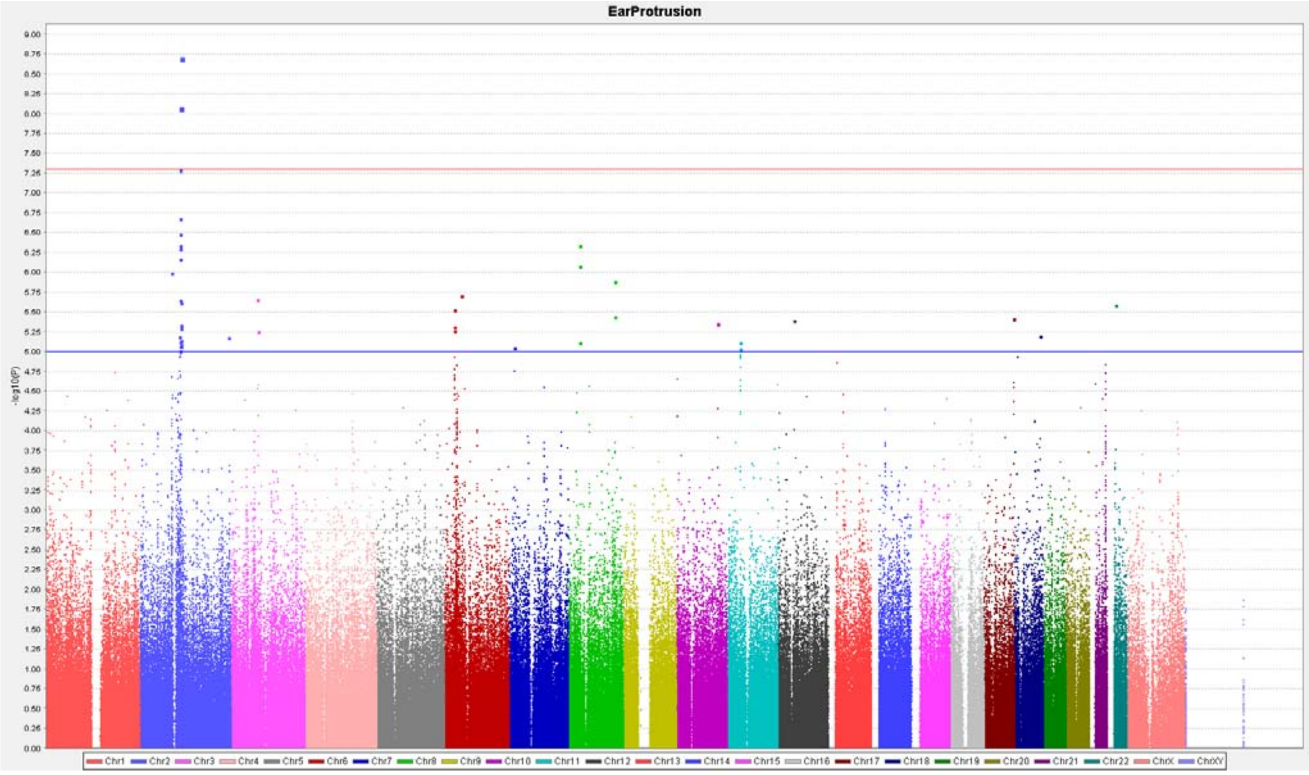

Antitragus Size:

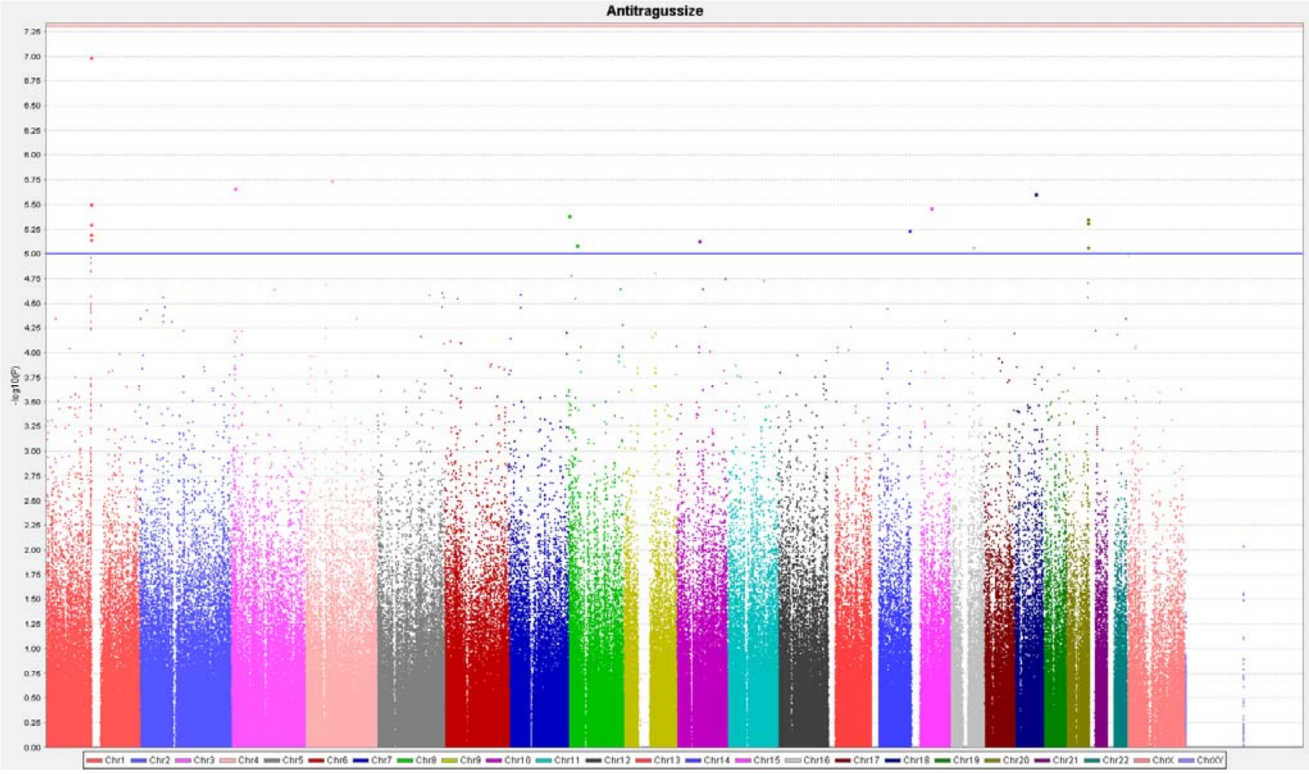

Folding of Antihelix:

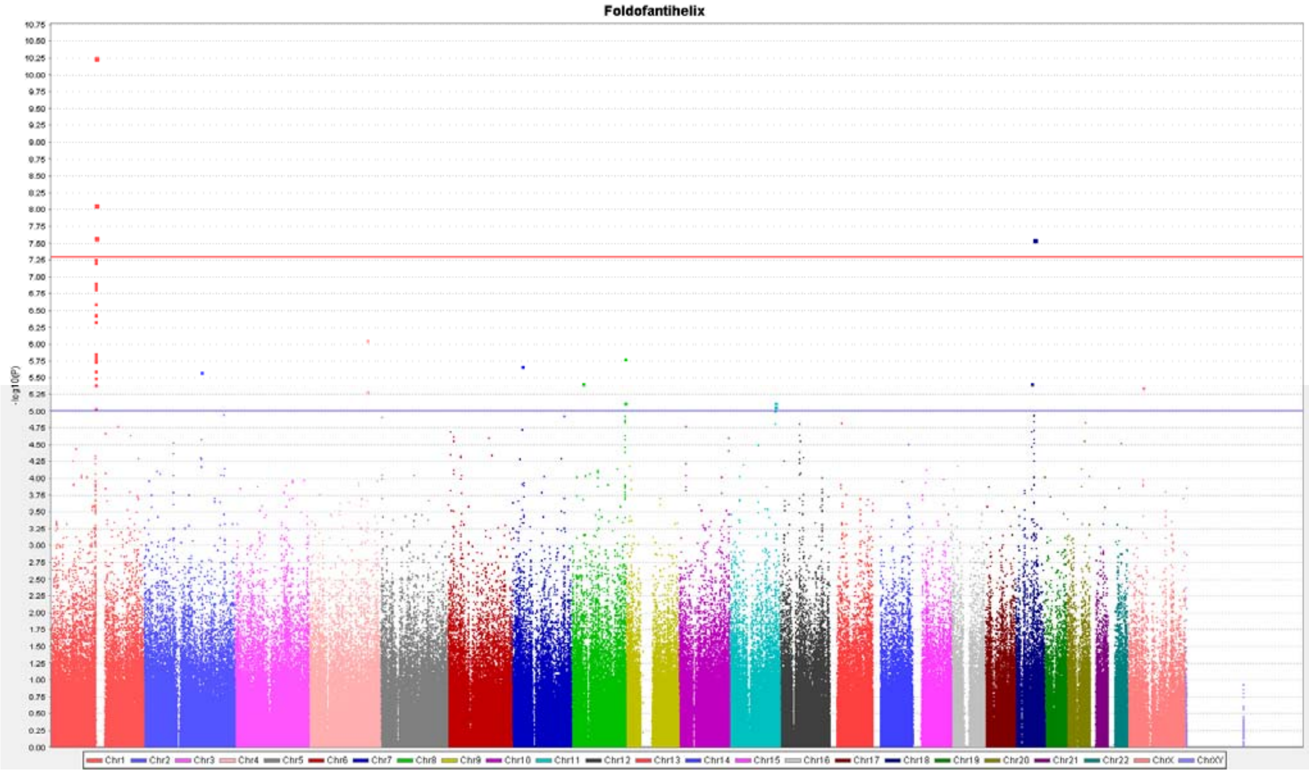

Helix Rolling:

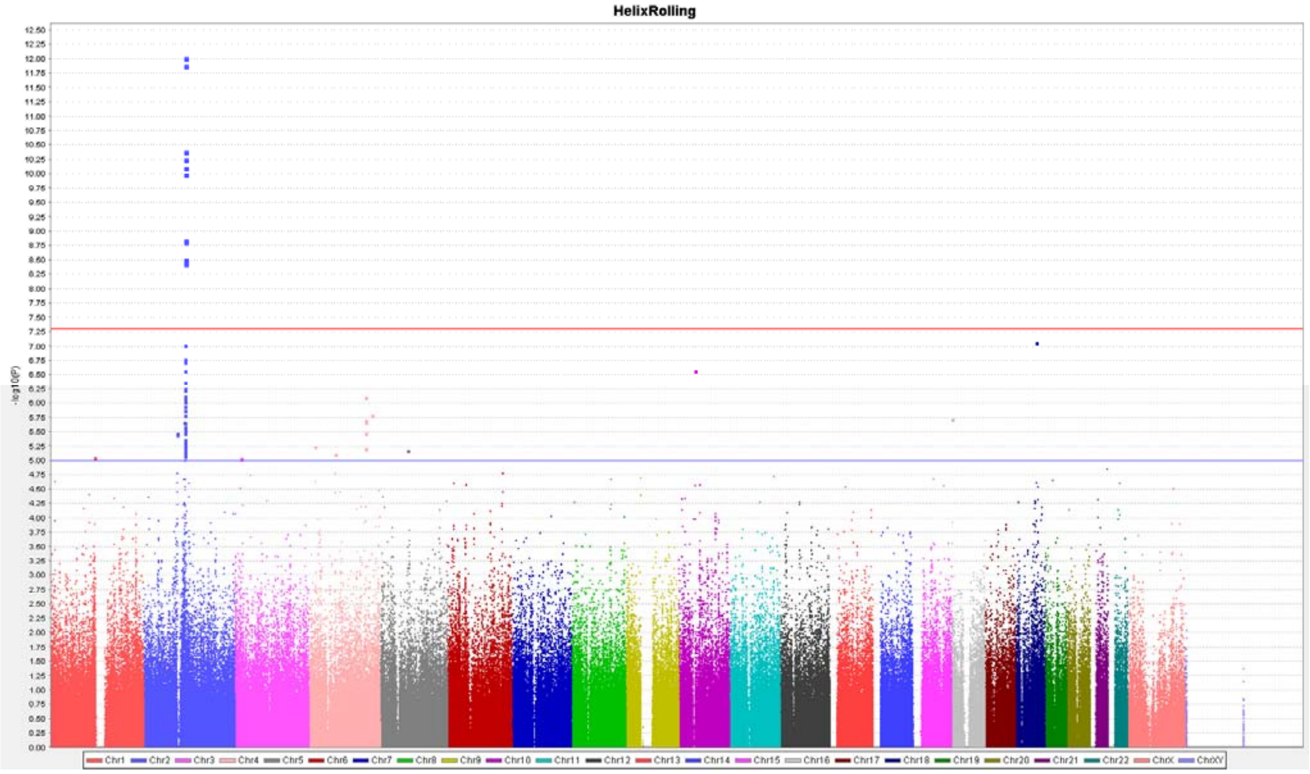

Lobe Attachment:

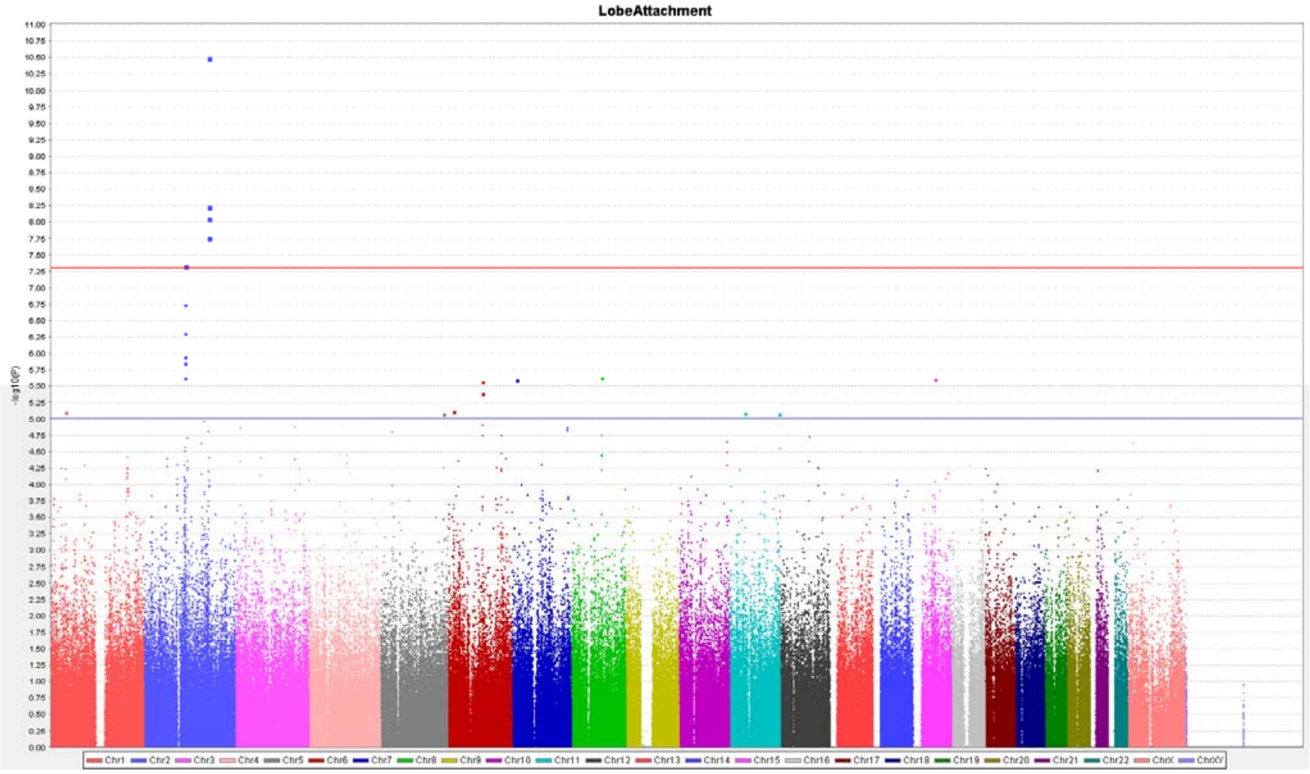

Lobe Size:

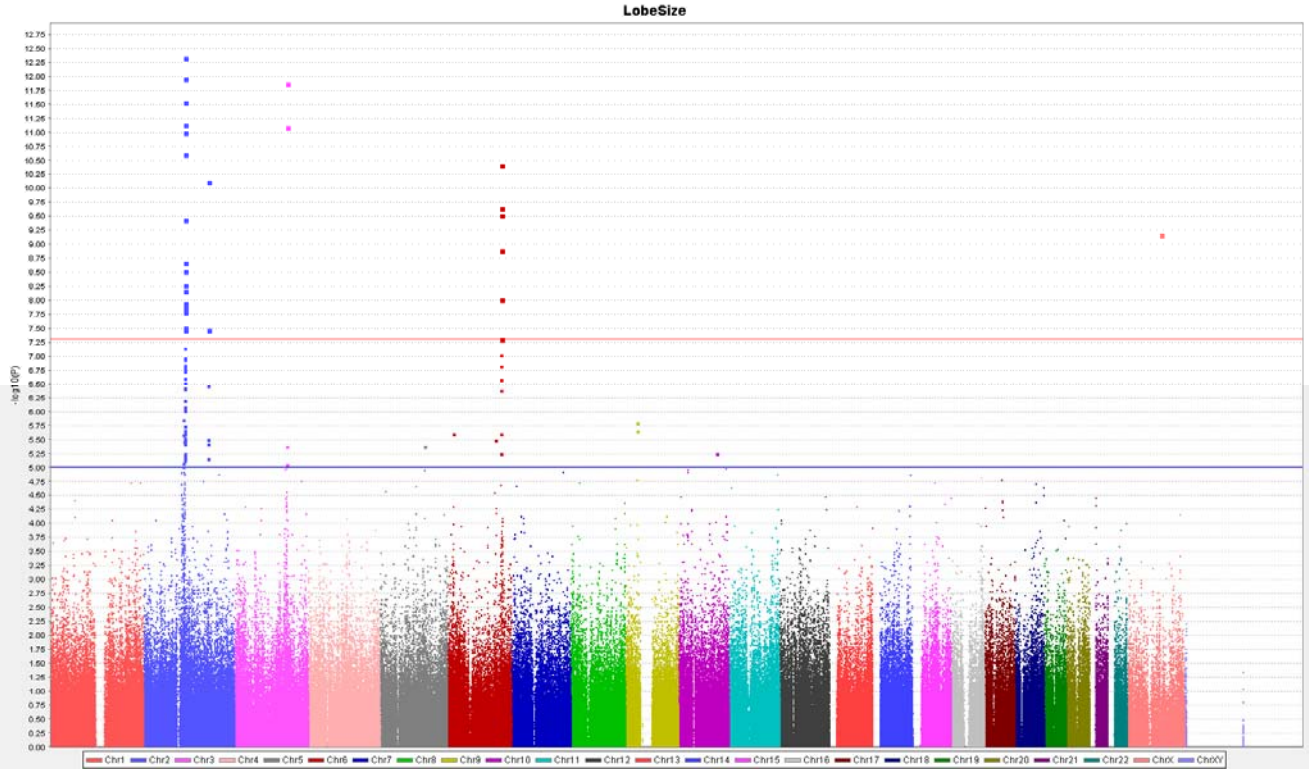
LobeSize
-log10(P)
Chr1 Chr2 Chr3 Chr4 Chr5 Chr6 Chr7 Chr8 Chr9 Chr10 Chr11 Chr12 Chr13 Chr14 Chr15 Chr16 Chr17 Chr18 Chr19 Chr20 Chr21 Chr22 ChrX ChrXY

Supplementary Figure 7: Regional association plots for the seven genomic regions showing genome-wide significant association to pinna traits.

Results are shown for all traits showing genome-wide association (Table 1). Association results (on a $-\log_{10}$ P scale; left y-axis) are shown for markers ~500kb on either side of the index SNP (i.e. the marker with smallest p-value, purple diamond; Table 1) with the marker (dot) colour indicating the level of LD (r^2) between the index SNP and that marker in the 1000genomes AMR dataset. Local recombination rate in AMR is shown as a continuous blue line, with the scale on the right y-axis. Genes in each region, their intron-exon structure, direction of transcription and genomic coordinates (in Mb, using the NCBI human genome sequence, Build 37, as reference) are shown at the bottom. Plots were produced with LocusZoom.

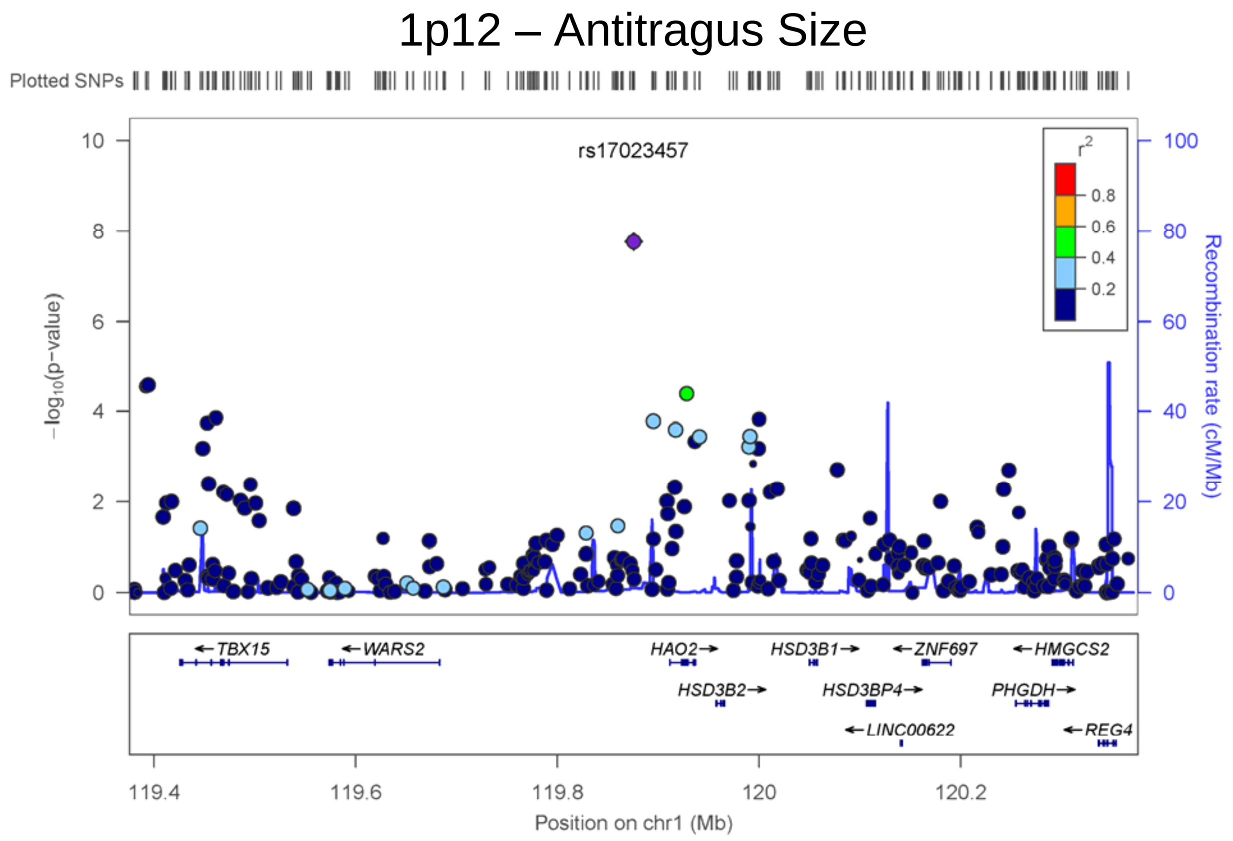

1p12 – Folding of Antihelix

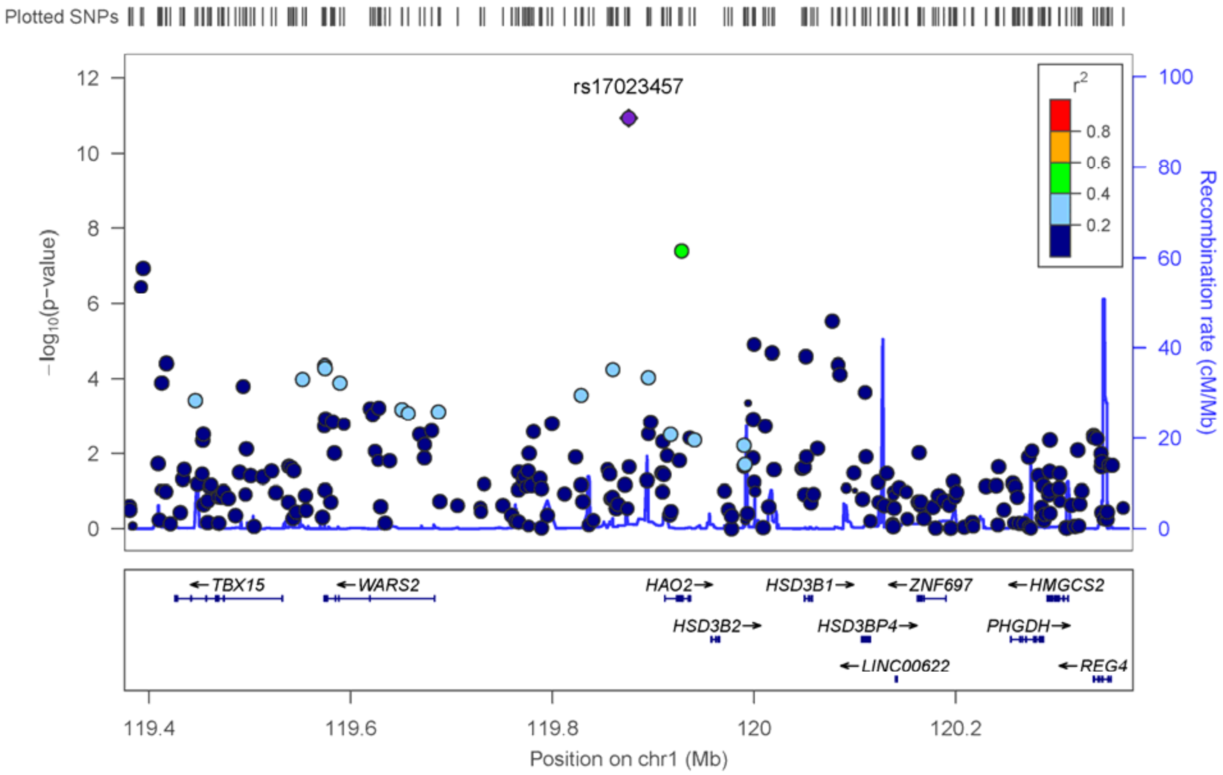

2q12.3 – Ear Protrusion

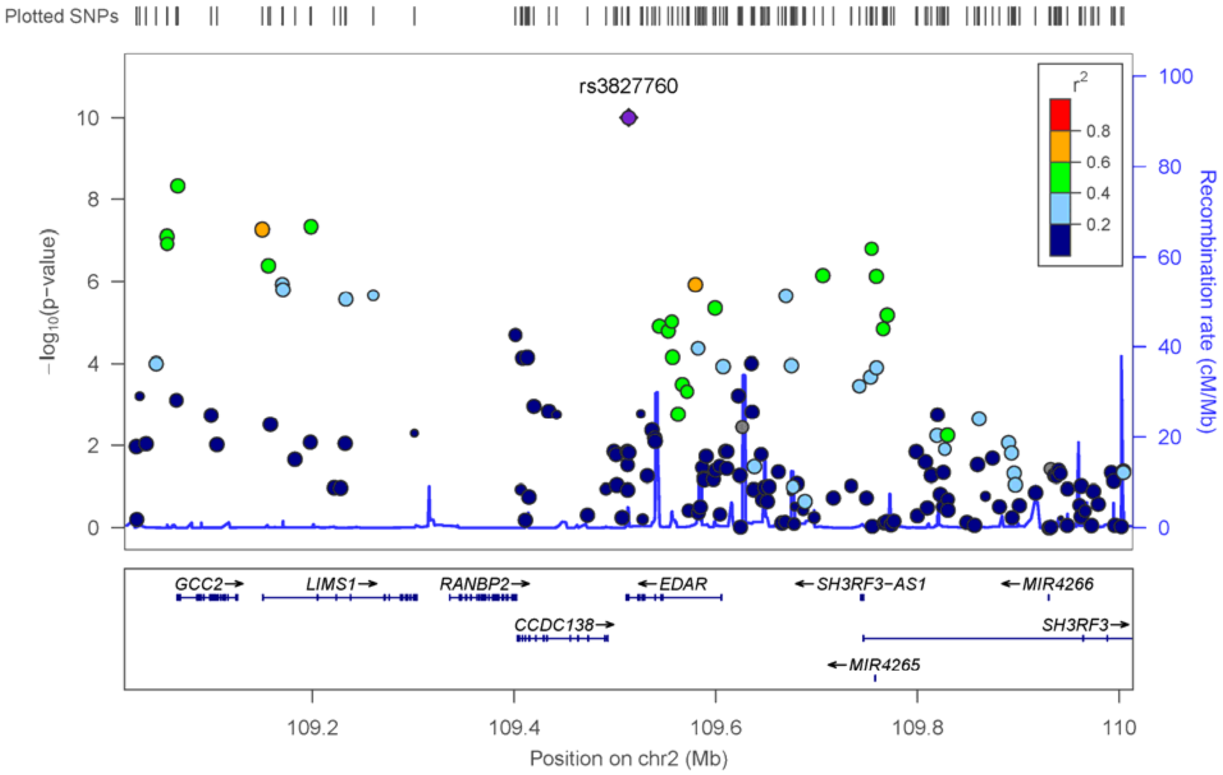

2q12.3 – Helix Rolling

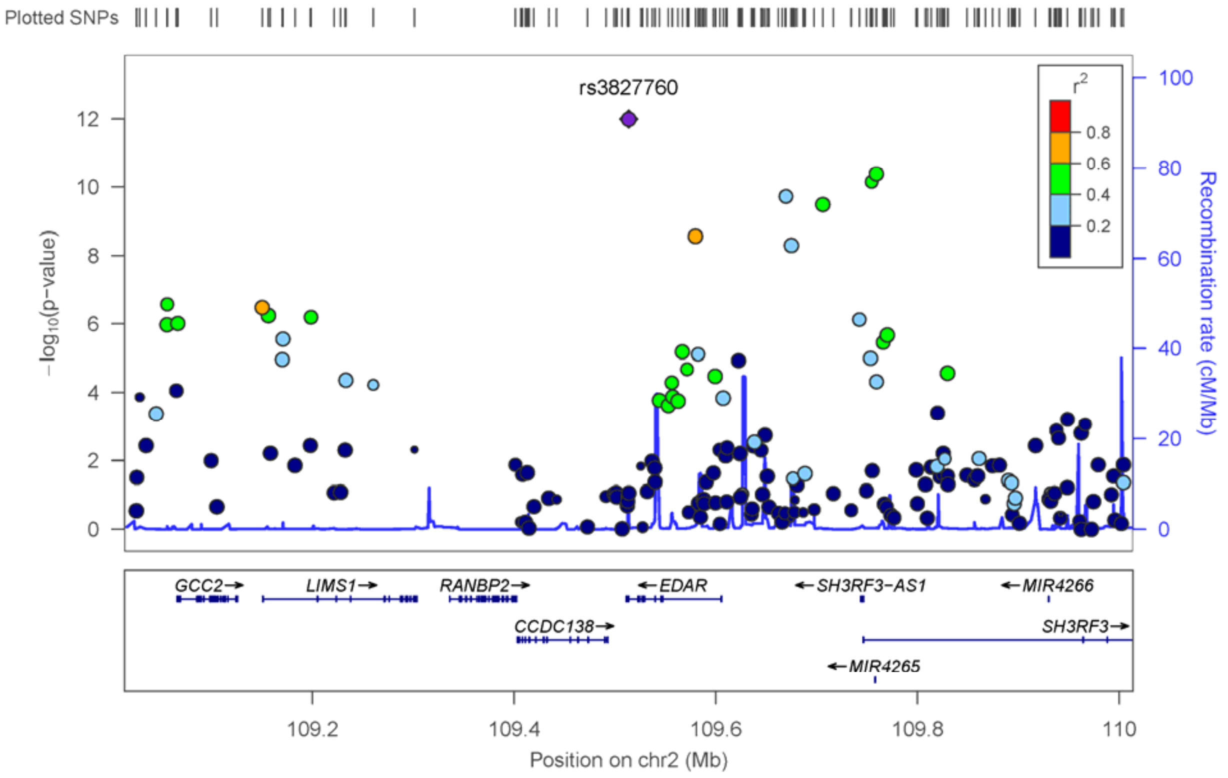

2q12.3 – Lobe Attachment

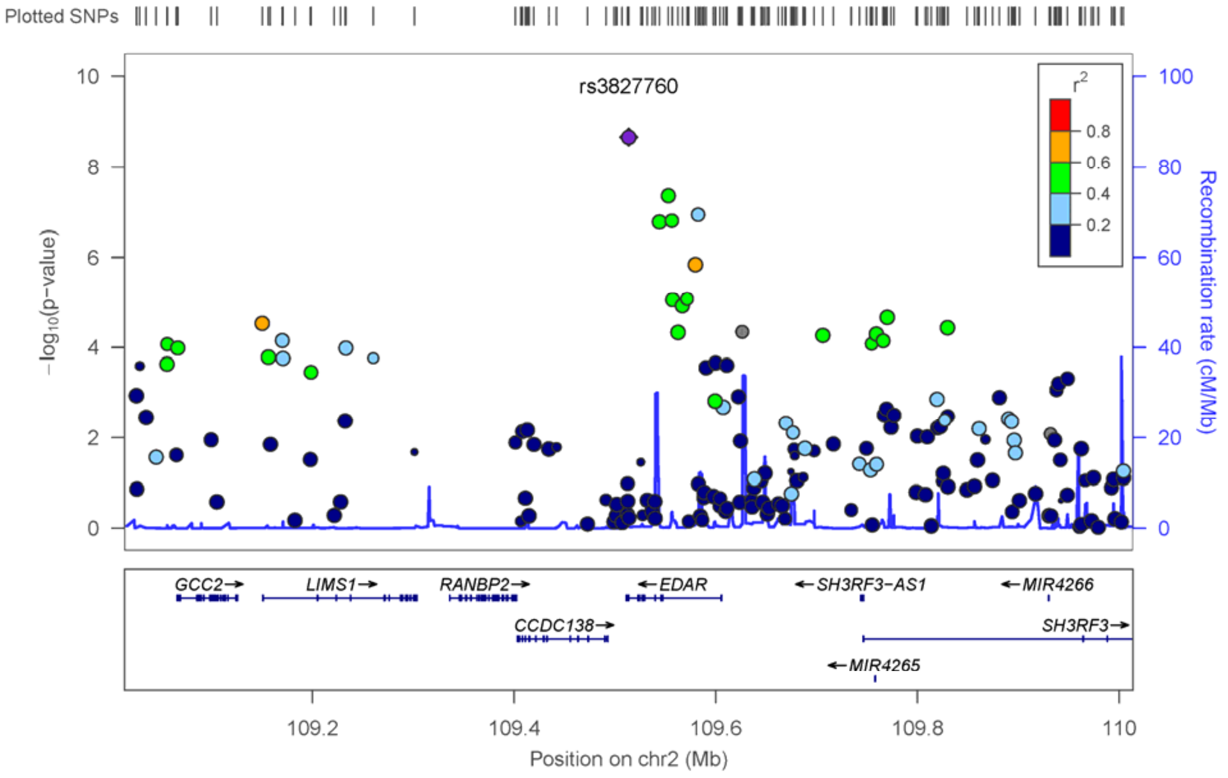

2q12.3 – Lobe Size

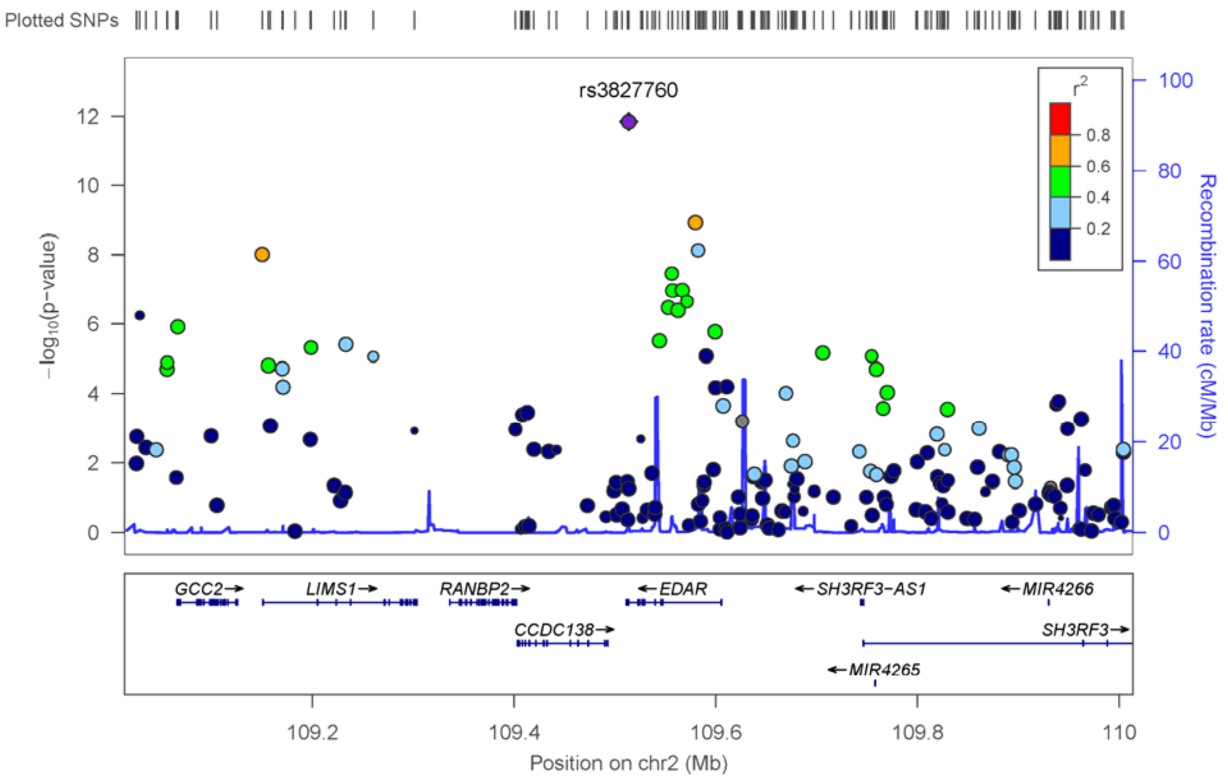

2q31.1 – Lobe Attachment

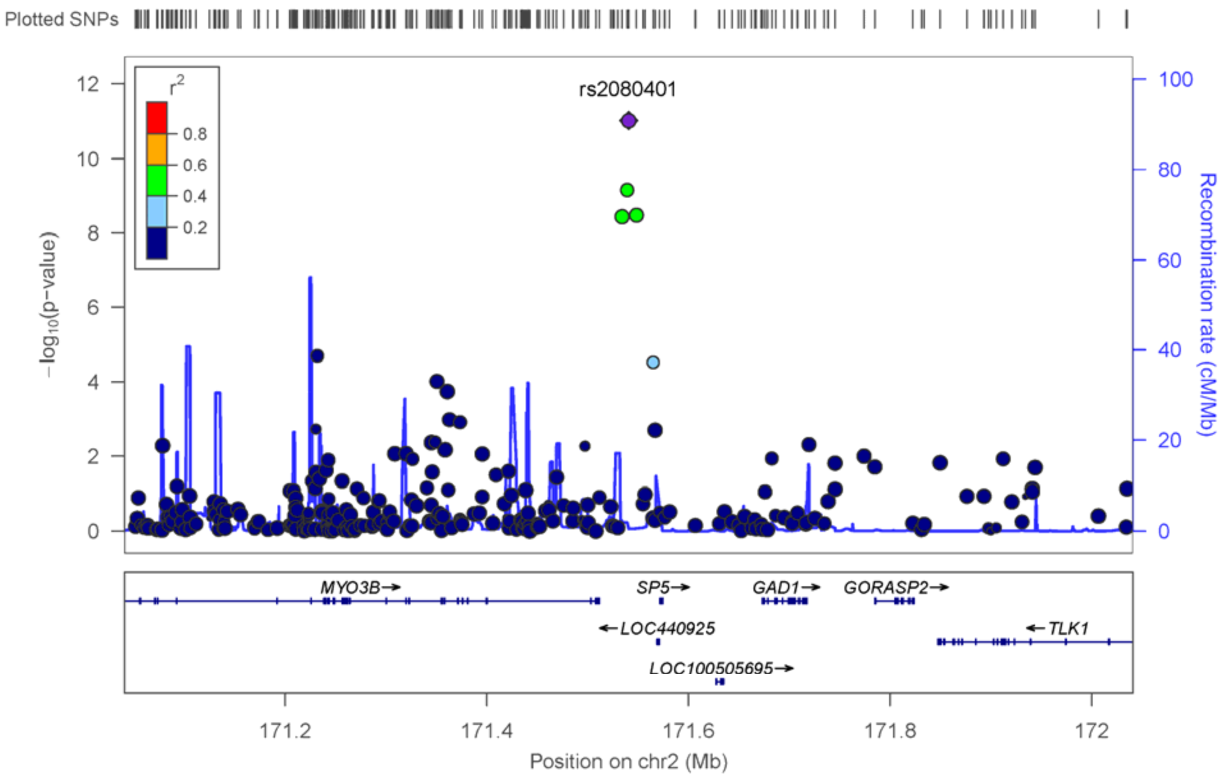

2q31.1 – Lobe Size

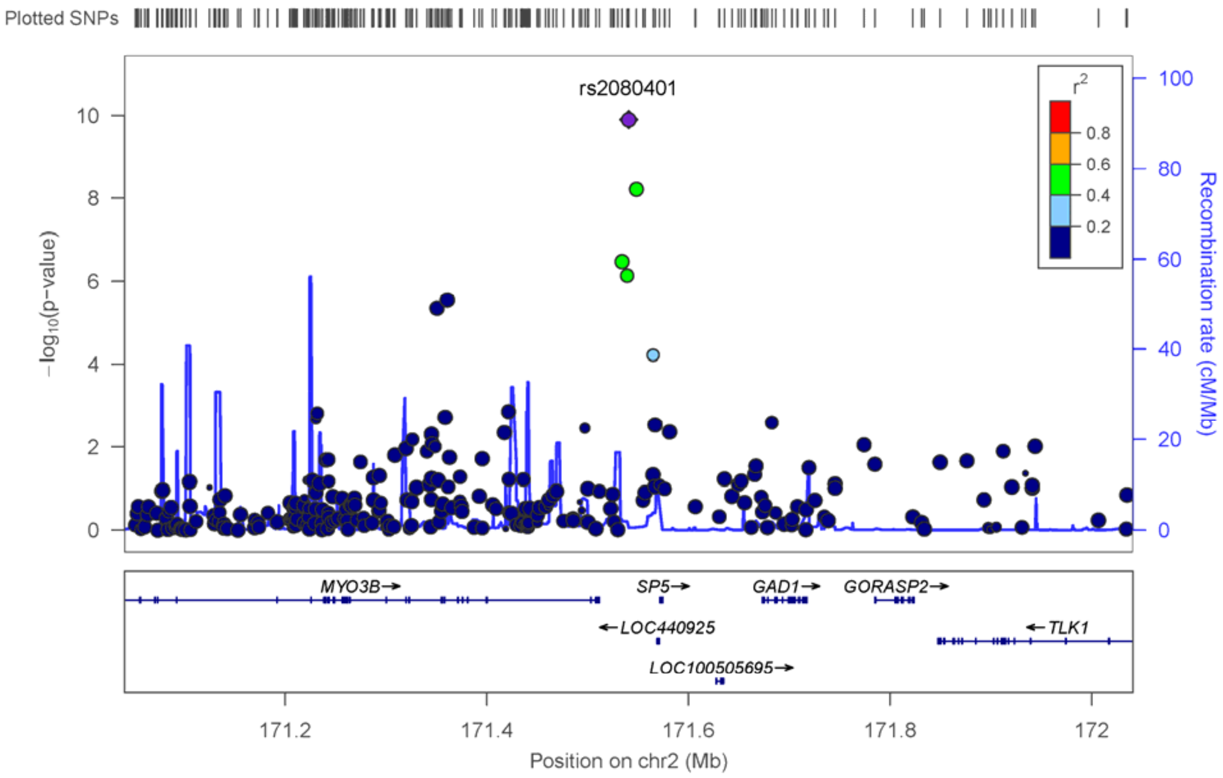

3q23 – Lobe Size

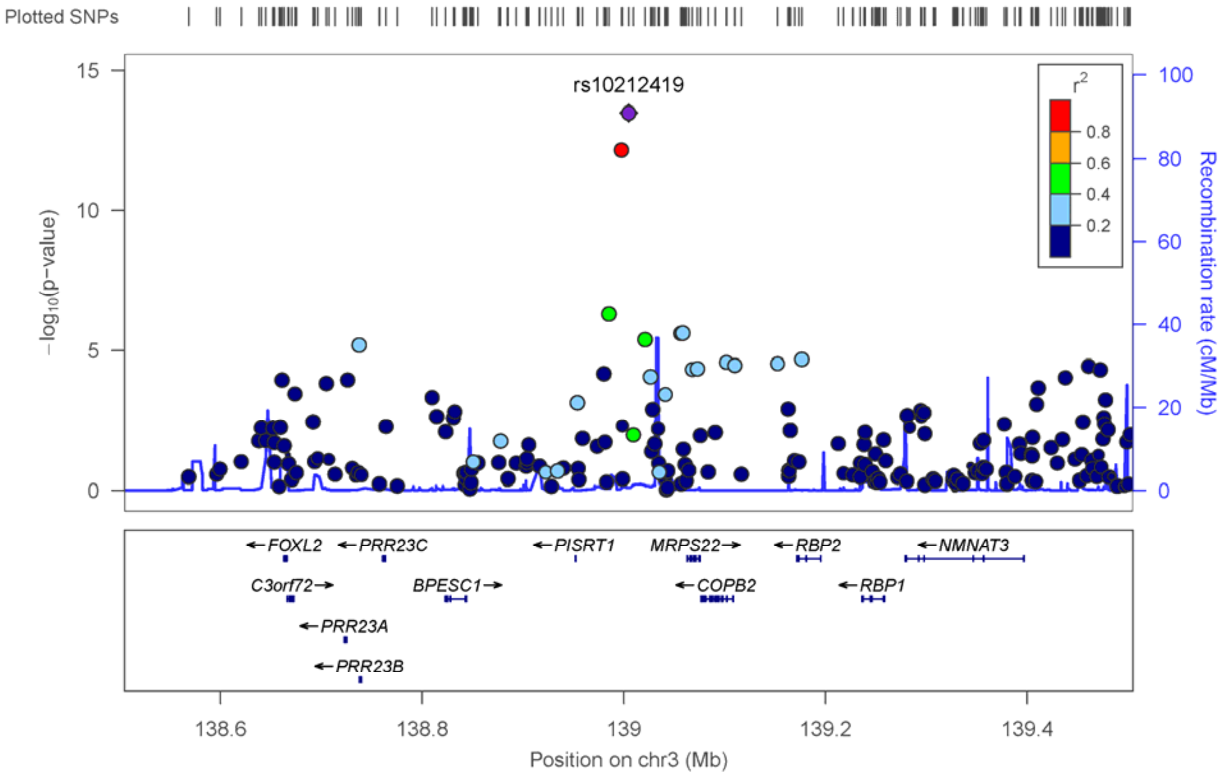

4q31.3 – Helix Rolling

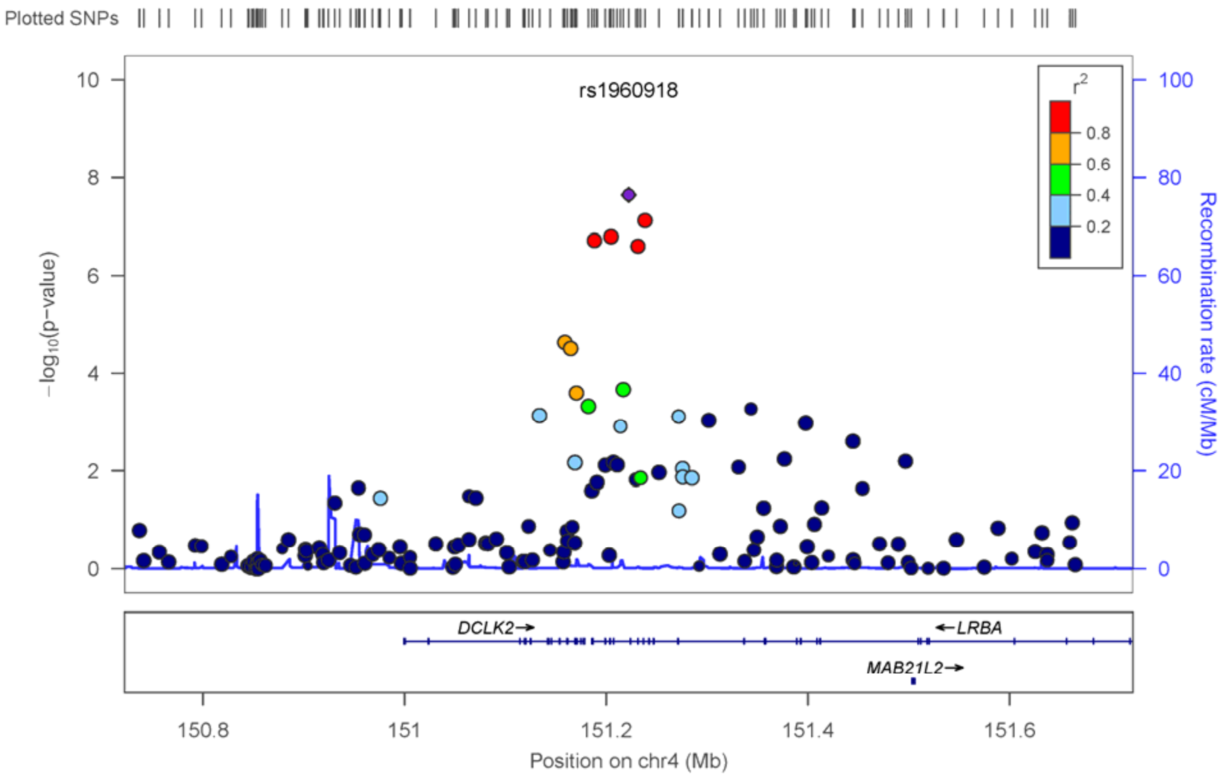

6q24.2 – Lobe Size

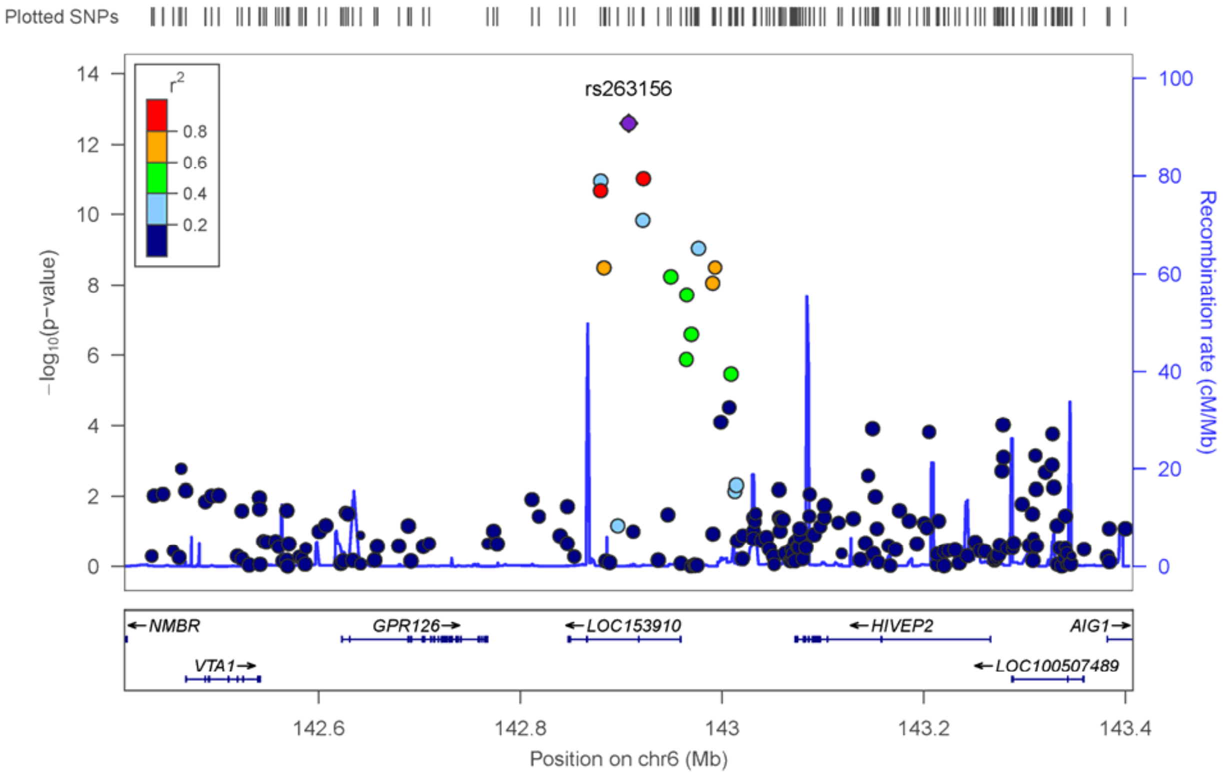

18q21.2 – Folding of Antihelix

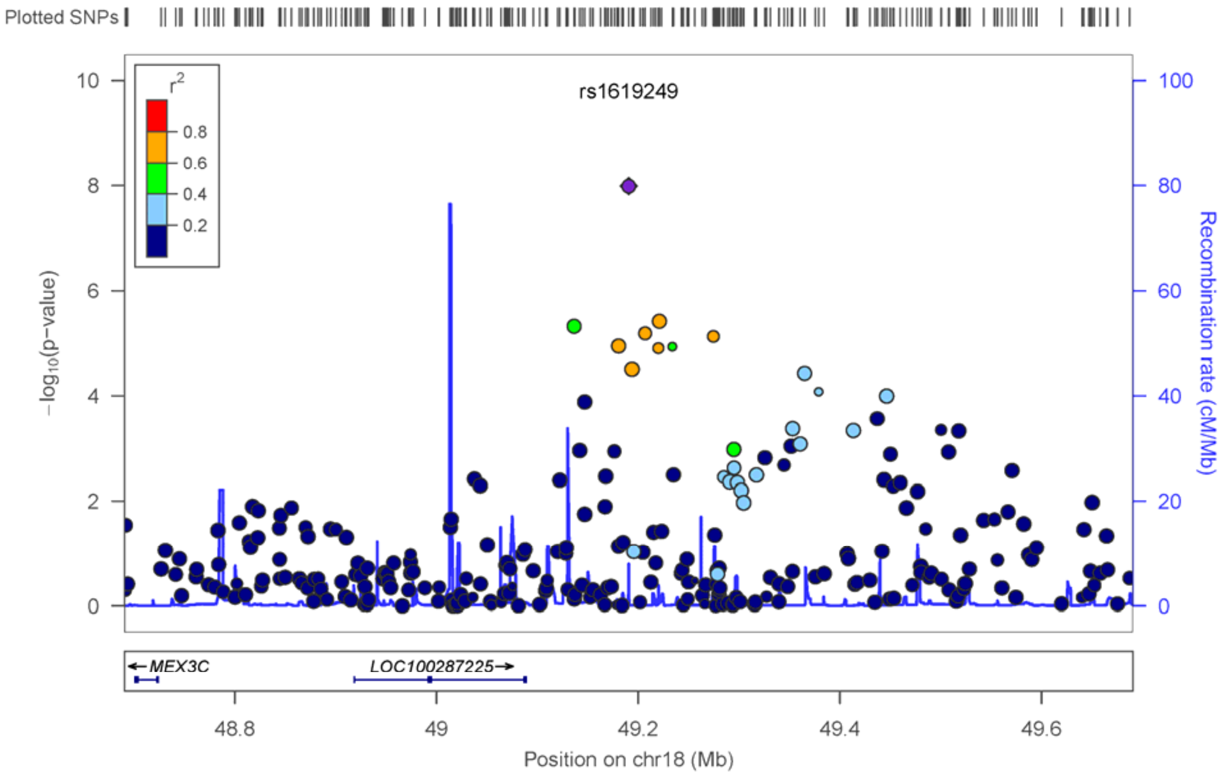

Supplementary Figure 8: Conditioned GWAS analysis

A) rs3827760 on Lobe Size

The two figures below compare GWAS Manhattan plots for Lobe Size (top) and Lobe Size conditioned on rs3827760 (bottom).

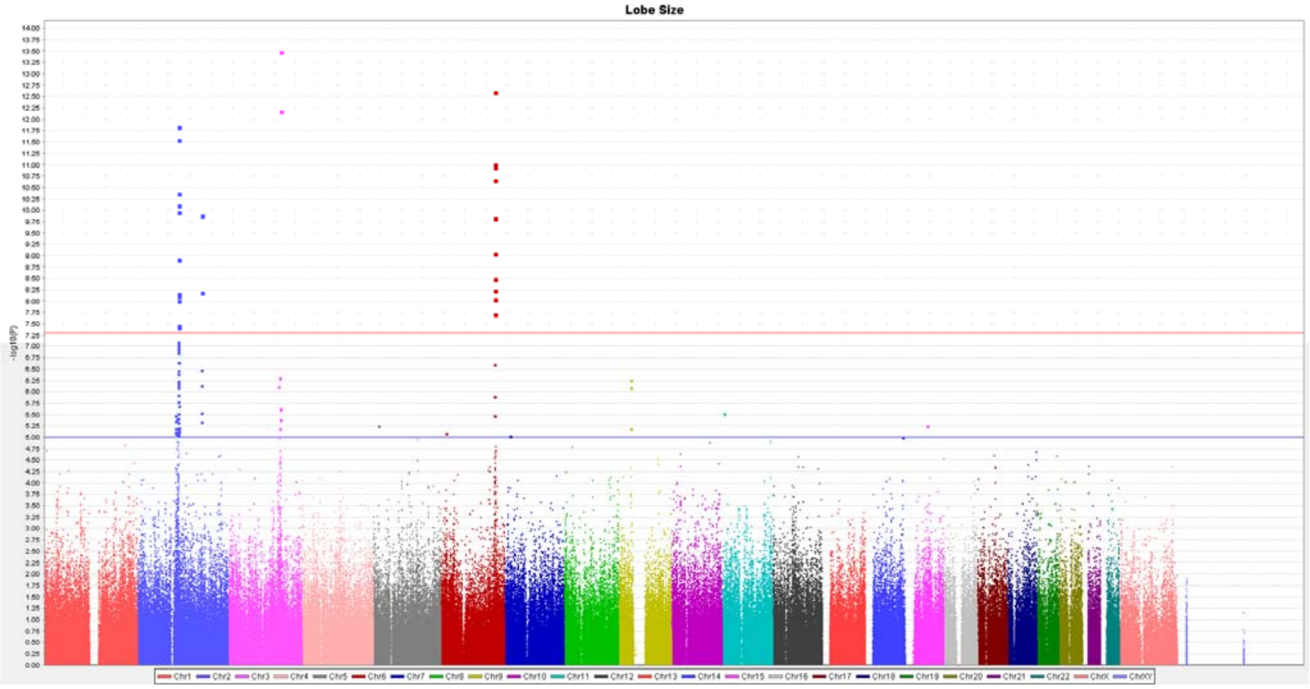

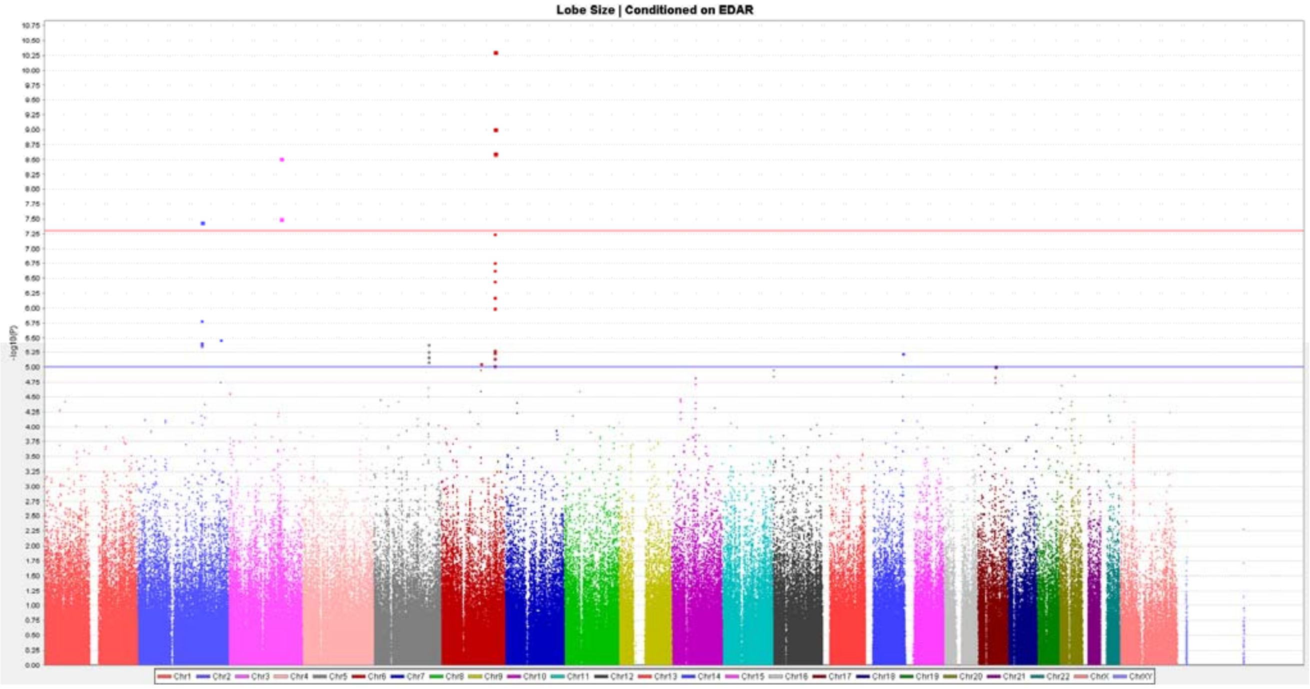

B) rs17023457 on Folding of Antihelix

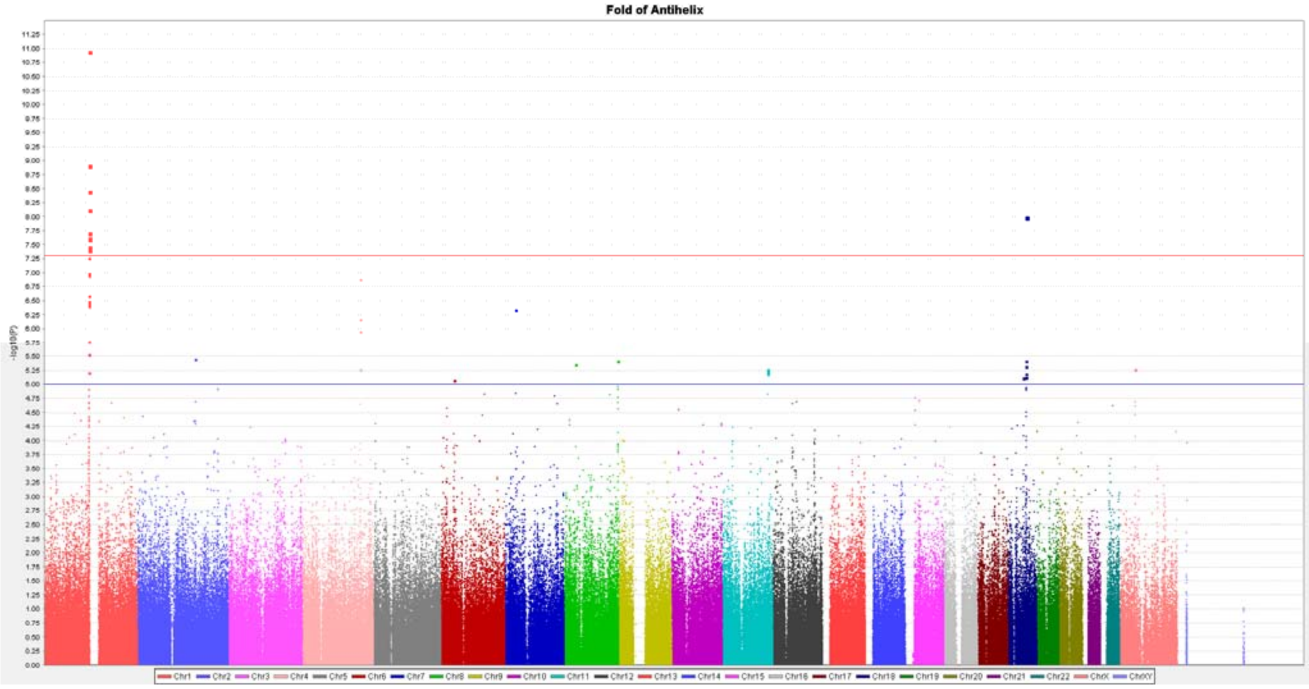

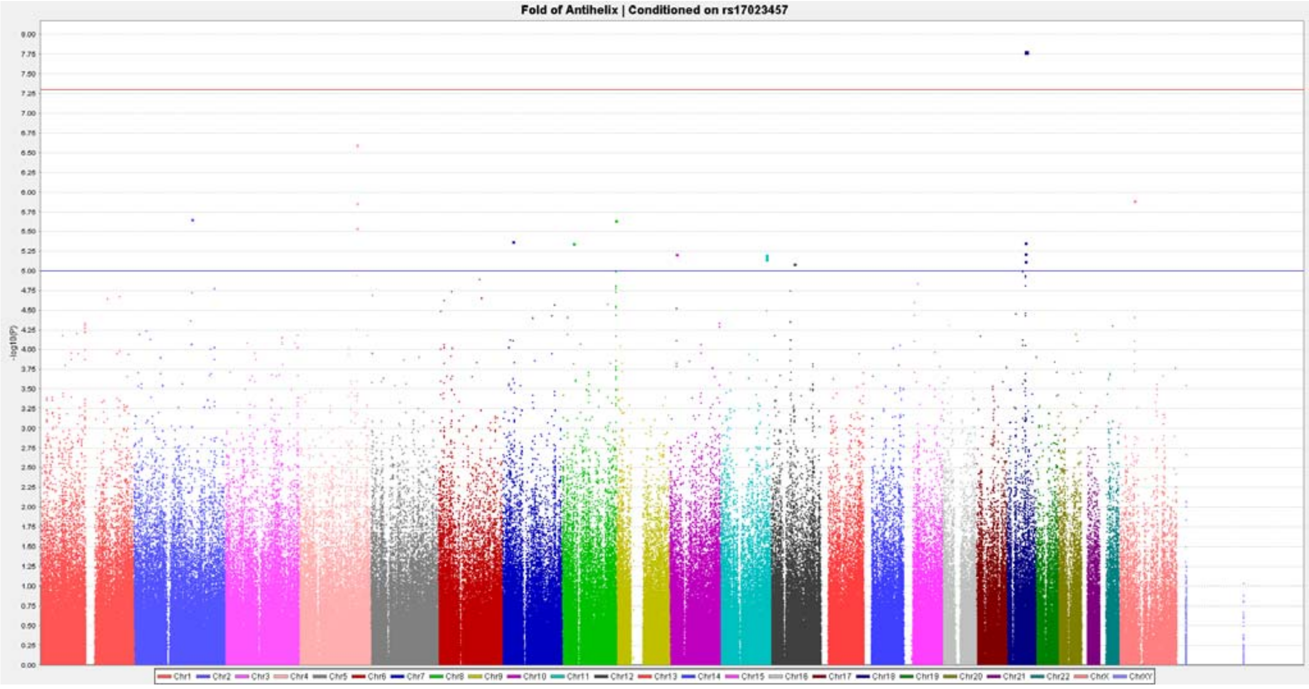

Supplementary Figure 9: Geometric-Morphometric evaluation of mouse pinnae

A) Top head view:

The placement of ten landmarks on top views of a mouse head is shown below. Eight points are on the mouse head and two on the scale for calibration of distance.

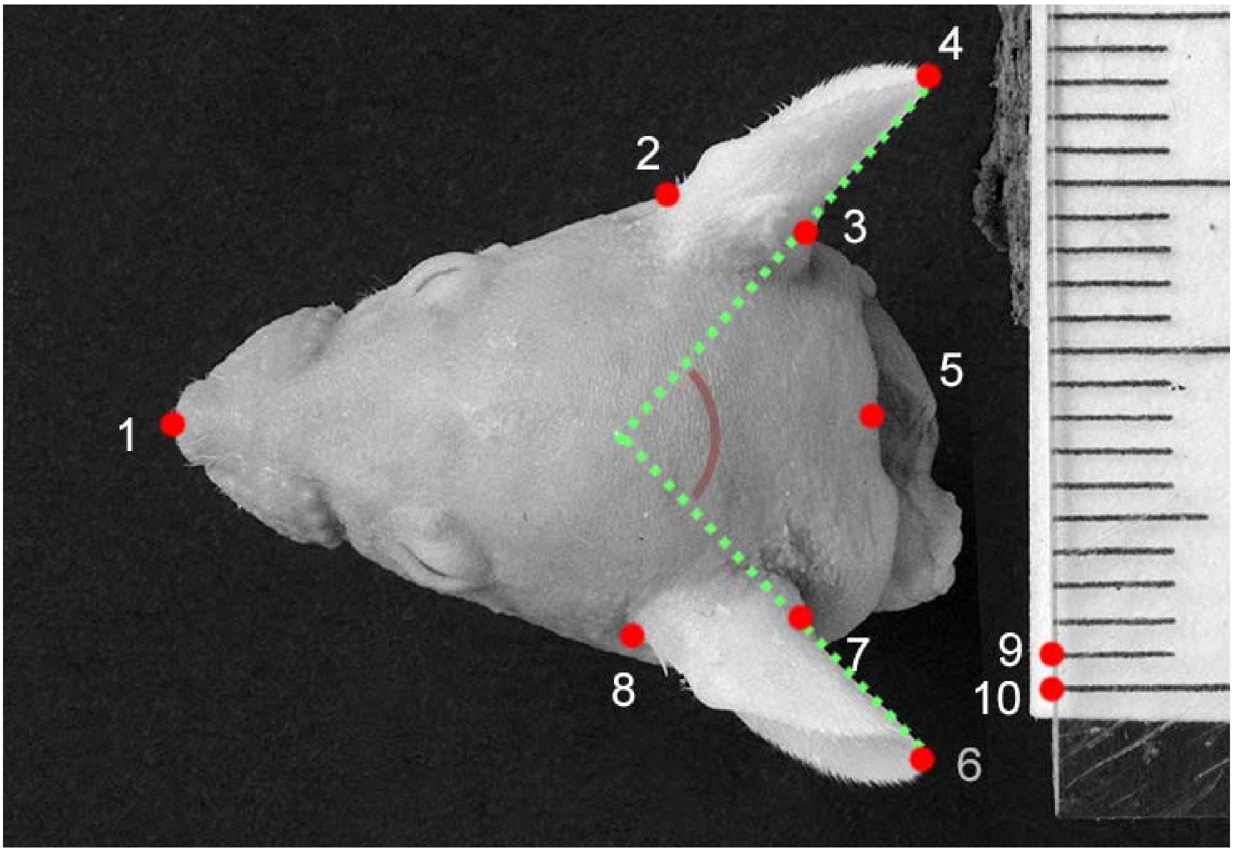

Landmark definitions:

Landmark	Name	Definition
1	Nose tip	The tip of the nose (most anterior point of the nose in the superior view).
2	Otobasion superiorous R	The right superior point at the attachment of the pinna and the head.
3	Otobasion posterior	The most posterior point where the right pinna and the head meet.
4	Superpinna	The most posterior-superior point of the right pinna in the superior view of the head.
5	Opisthocranion	The most posterior point of the head, at the sagittal plane.
6	Superpinna	The most posterior-superior point of the left pinna in the superior view

		of the head.
7	Otobasion posterior	The most posterior point where the left pinna and the head meet.
8	Otobasion superiorous L	The left superior point at the attachment of the pinna and the head.

Ear protrusion is measured in two ways:

1) The angle between two pinna, shown as the angle between two green lines (lines joining landmarks 3-4 and 6-7), in degree units.
2) The tip distance, measured as the distance between landmarks 4 & 6. This was calibrated by the distance between landmarks 9 & 10 being 1 mm. It was converted as a proportion via dividing by head width.

B) Side head view:

The placement of 27 landmarks on 2D photographs of the left side of mice heads is illustrated below. 24 landmarks are placed on the pinna (4 to 27) whereas 3 (1-3) are placed on the head. Centroid size and principal components were calculated on this set of pinna landmarks and semi-landmarks after sliding of semi-landmarks and Generalized Procrustes Analysis.

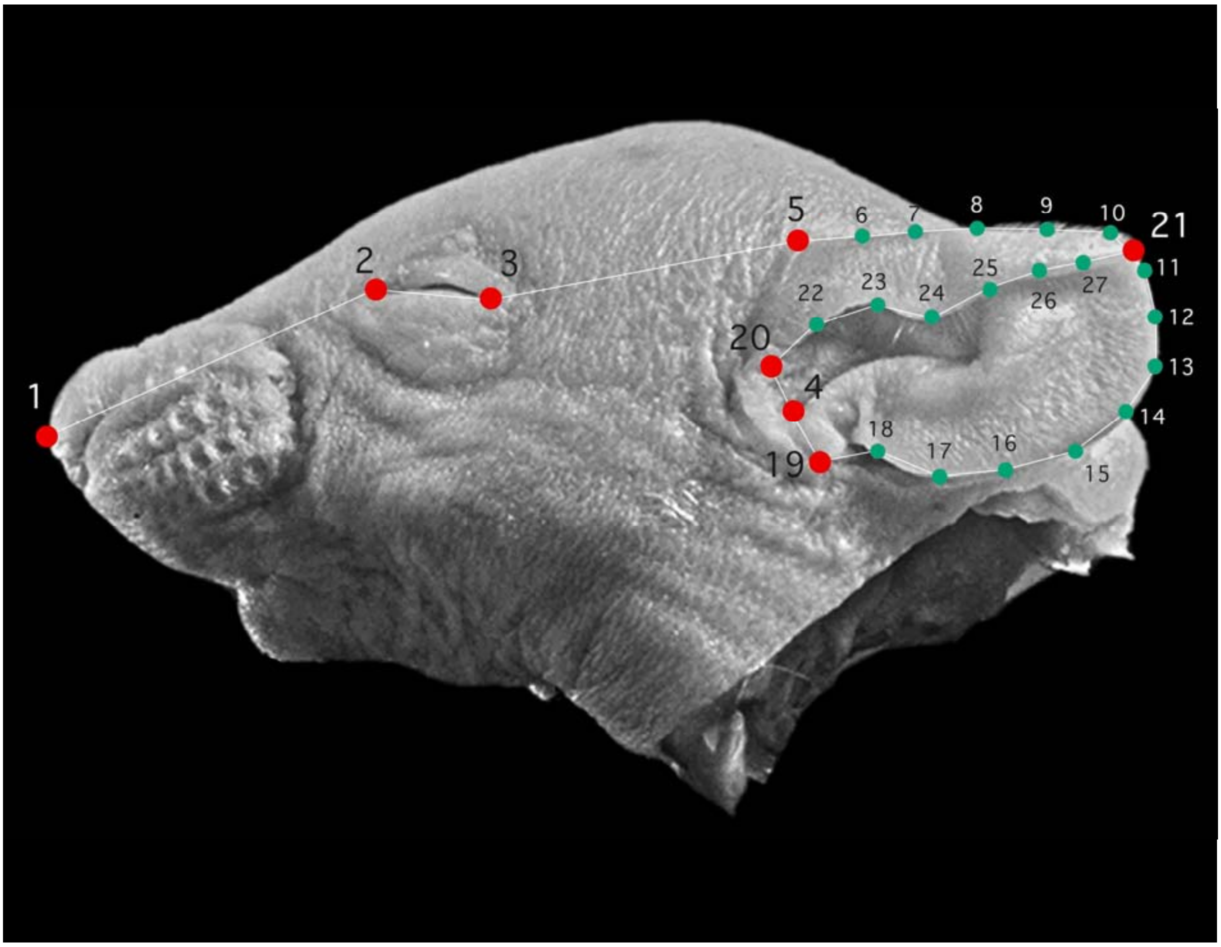

Landmark definitions:

Landmark	Name	Definition
1	Nose tip	The tip of the nose (most anterior point of the nose in the lateral view).
2	Endocanthion	The inner corner of the eye fissure where the eyelids meet.
3	Exocanthion	The outer corner of the eye fissure where the eyelids meet.
4	Intertragic incisures	The deepest point in the intertragal incisures.
5	Otobasion superiorous	The superior point on the union of the pinna and the head.
6 – 10	Semi-landmarks*	Points along the superior border of the helix, between landmarks 5 and 21.
11- 18	Semi-landmarks*	Points along the inferior border of the pinna (auricle), between landmarks 21 and 19.

19	Otobasion inferiorous	The basal point on the union of the pinna and the head.
20	Helix anterior	The most anterior point where the internal border/line of the helix and the head meet.
21	Helix posterior	The most posterior point where the internal border/line of the helix and the head meet.
22- 27	Semi-landmarks*	Points along the inferior border of the helix, between landmarks 20 and 21.

*Semi-landmarks are treated as homologous landmarks after sliding them along the contour minimizing the bending energy matrix of the configuration (see Methods).

Supplementary Figure 10: Effect of *Edar* genotype on mutant mouse pinna morphology

We compared pinna morphology between wild-type and mutant mice. The loss of function $Edar^{dlJ}$ line carries the downless Jackson allele (encoding Edar p. E379K). The gain of function $Edar^{Tg951}$ line carries approximately 16 copies of the Edar gene copies of the entire Edar locus on a 200 Kb yeast artificial chromosome (homozygous transgenic animals therefore carry about 34 copies of the gene in total). More details in Supplementary Note 1. The number of individuals examined for each genotype is provided in brackets.

A) Boxplot of ear protrusion angle vs. genotype

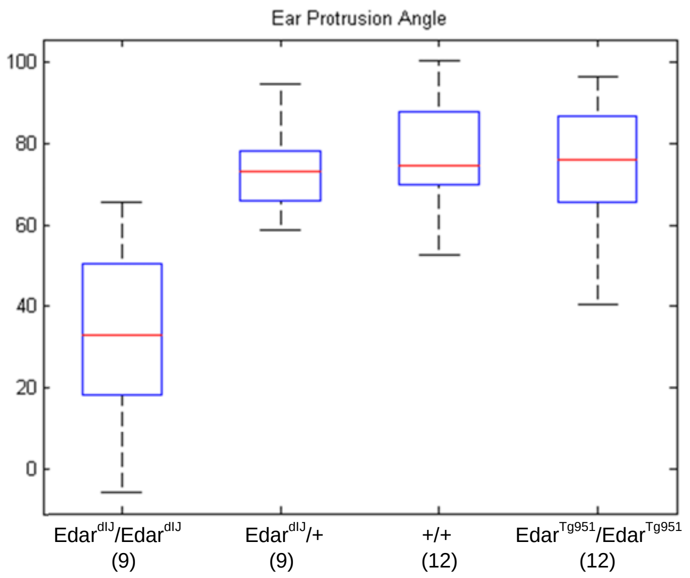

B) Boxplot of ear tip distance (as a proportion of head width) vs. genotype categories:

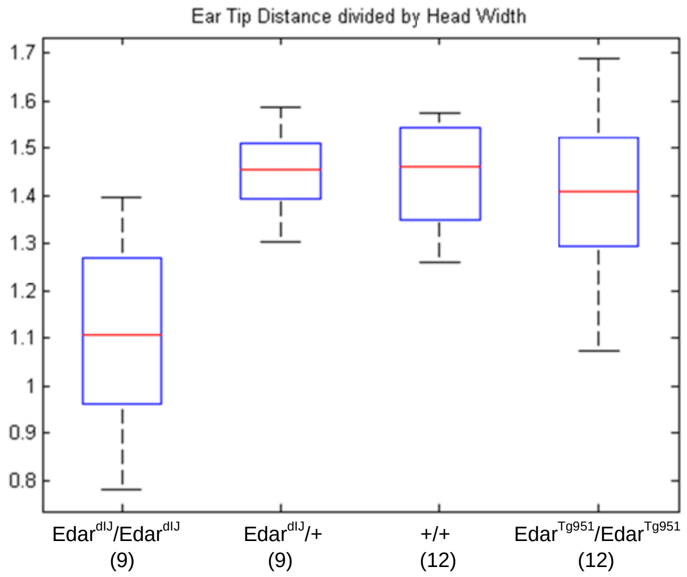

C) Boxplot of 2D pinna landmarks PC1 vs. genotype categories:

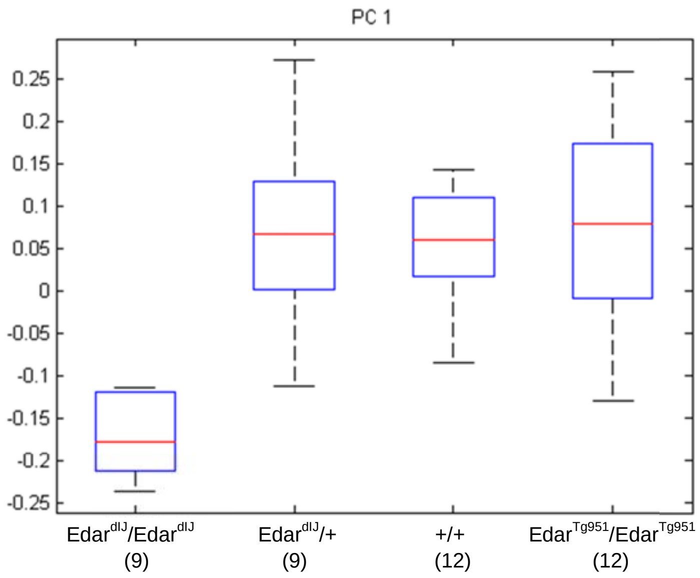

D) Interpretation of mouse pinna landmark PC1 shape change:

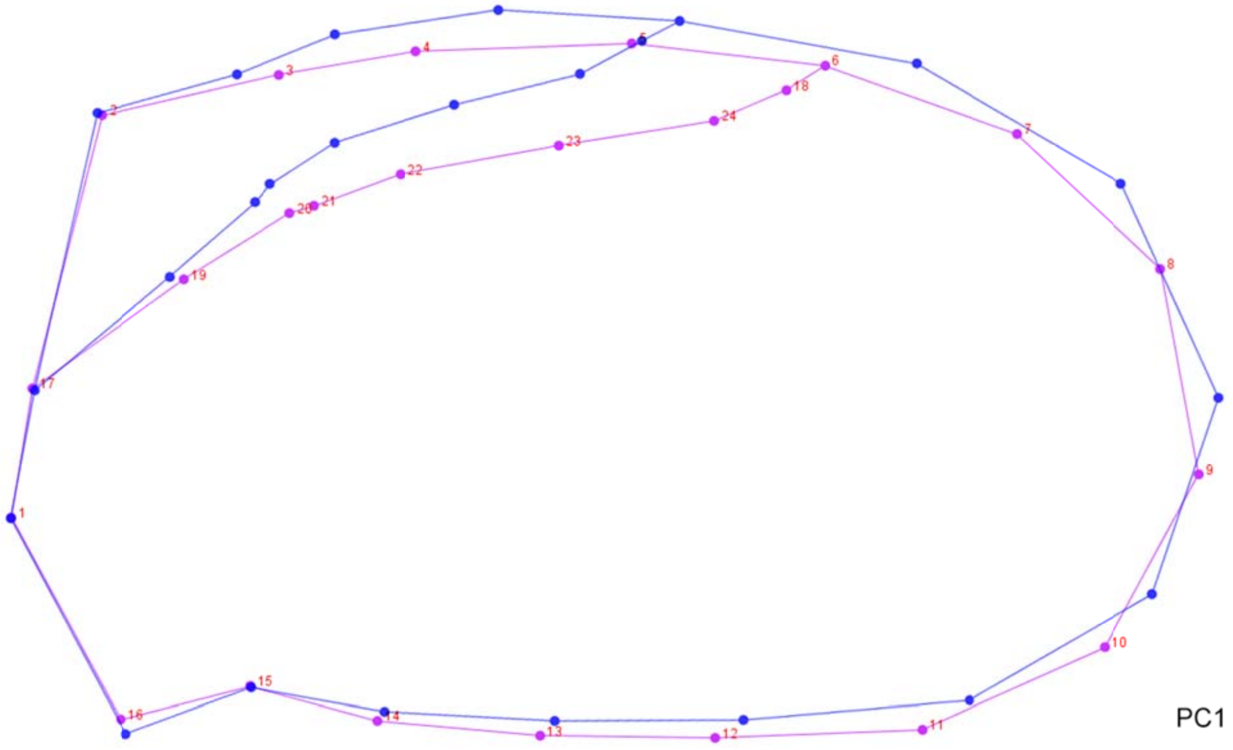

Wireframe of the average shape of mouse pinna landmark points is shown in purple, while the wireframe distorted towards PC1 by 10% is shown in blue. For simplicity, the three landmarks placed in the head are not shown, and the two wireframes are rotated to coincide at the base of the pinna (landmarks 4, 5, 19, 20 of the side view protocol) in order to visualize change in other landmarks (an analysis based only on pinna landmarks provided highly similar results). Landmarks corresponding to the border of helix (path joining landmarks 20 to 21 in protocol) show the largest deviation. While the mouse pinna does not show the rolling seen in humans, a correspondence can be envisioned by observing how further the helix is attached to the outer boundary of the pinna. PC1 is strongly associated with EDAR genotypes as seen in the previous subsection, which corresponds to the association of helix rolling to rs3827760 seen in the human pinna.

Supplementary Figure 11: *In-silico* and *in-vitro* analysis of the index SNP in the 1p12 associated region (rs17023457)

A) rs17023457 is located in an evolutionarily conserved binding site for transcription factor CART1 (Cartilage paired-class homeoprotein 1)

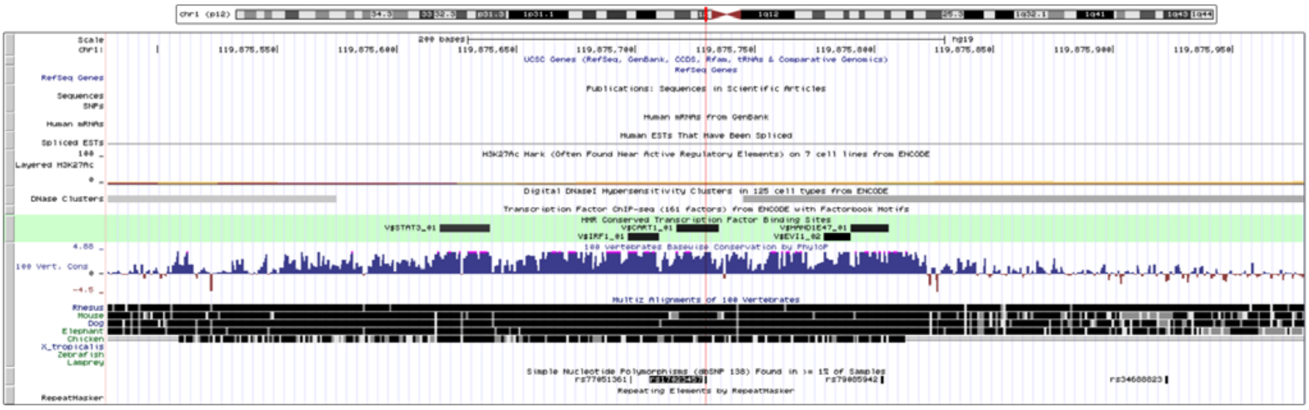

The green region highlights conserved transcription factor binding sites (TFBS) . The CART1 binding site overlaps with rs17023457 (its position denoted by the vertical red line). The blue barplot panel underneath the TFBS track indicates high evolutionary conservation of this non-coding region (100 Vert. Cons track). Output from UCSC Genome Browser.

B) In vitro analysis of rs17023457

a) Electrophoretic mobility shift assays demonstrating differing DNA-protein interactions between probes (oligonucleotides) with the T or C alleles of rs17023457 using nuclear extracts from a CART1-expressing cell line, Huh7 (see Methods). A specific band is observed for the probe with the T allele which is eliminated when pre-incubating with a CART1 consensus competitor DNA sequence, confirming the binding of CART1. A non-specific band binds to probes with both rs17023457 alleles.

b) Luciferase reporter assay (see Methods) showing significantly increased expression in cells transfected with constructs containing the (T) allele at rs17023457, compared to constructs with the (C) allele. The genomic sequence surrounding rs17023457 (see paragraph C below) was inserted in forward (TF and CF) and reverse (TR and CR) orientations, upstream of the SV40 promoter driving luciferase expression. P-values were obtained from an analysis of variance of the luciferase readings.

c) Uncropped image of the EMSA gel as shown in a) above. The extra lane at the end is a duplicate of lane 2, i.e. CART1 competitor and CART1 consensus labelled probe.

a)

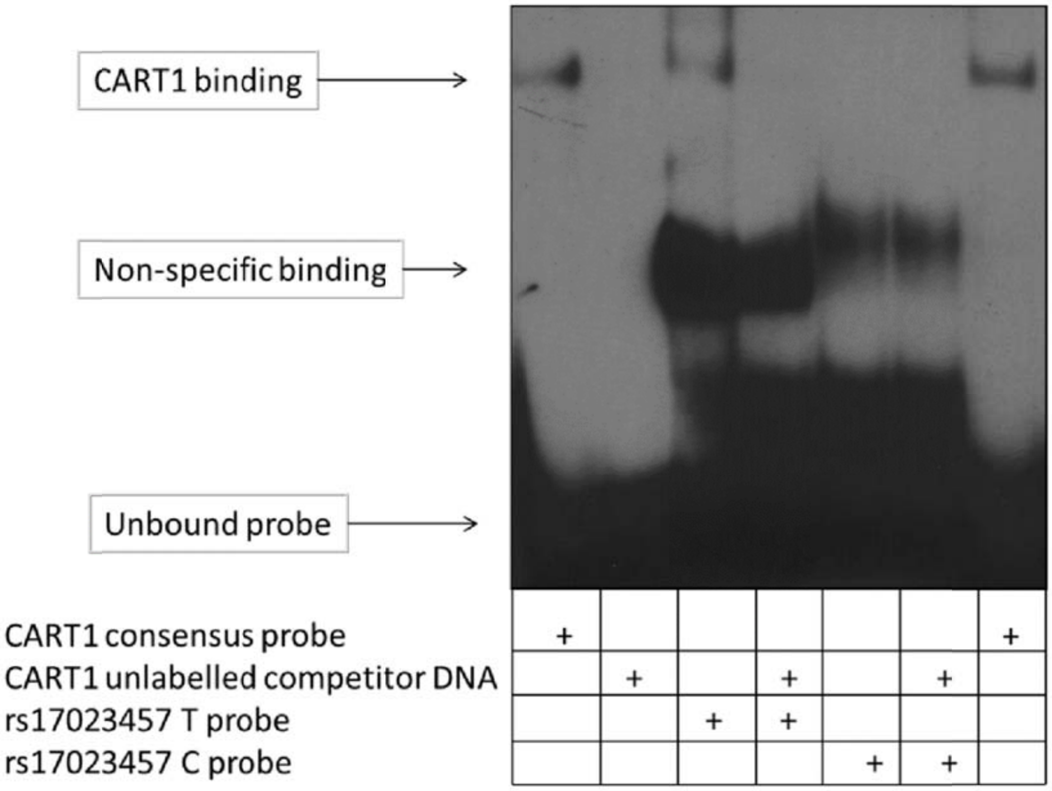
CART1 binding
Non-specific binding
Unbound probe
CART1 consensus probe
CART1 unlabelled competitor DNA
rs17023457 T probe
rs17023457 C probe
+

b)

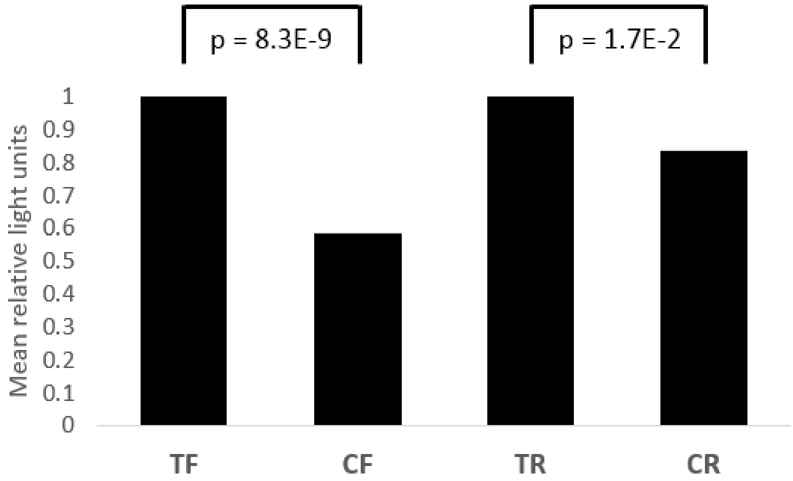
p = 8.3E-9
p = 1.7E-2
Mean relative light units
1
0.9
0.8
0.7
0.6
0.5
0.4
0.3
0.2
0.1
0
TF
CF
TR
CR

c)

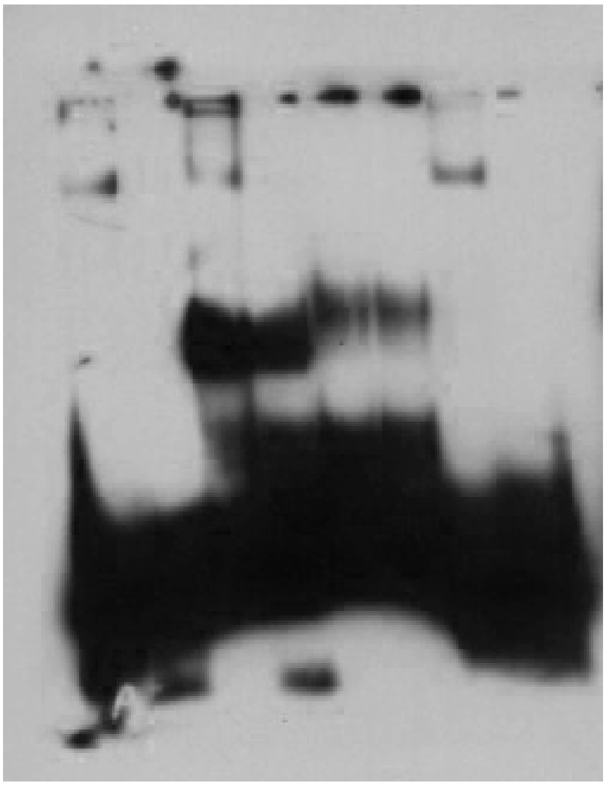

C) Sequence of genomic sequence insert (surrounding rs17023457) used in the luciferase reporter assay

Position of rs17023457 is highlighted in green:

```
GGTACCATCTACGGGTCTGGAGGAGGTTTGGGACGTTTCCTTTTCTTCTCTTCAGGGGCCCACTCCTGTTGTAAAG
AGCAGCAGCATTTATAATGAGGGCCCAGGAAATGCGGGGGAGAGGGGAGAGCCTGCTATCTCTGCAGTGGGTGACT
GTTGTTTCCCTGATCAACAATGTATCATATCCCAGCCTAAGCCTGCCTTTCTCCTTCCCCCTCCACATGGGGAGGG
GCCGTGGGCTCAGAGCTCACCTGGAATGCCTCTGGTTGCTGAAGGGCTGGATGGAGACTCTGAGACAACCTGATGG
ATCATCTCATAAACAGAGCCTGCAGGAAGGAAAAGAAAATTATGCCAGCATAATGCACTTACGAGAAATAGATACG
TGCGGTGCAGGGAGAAATGCTTTGATATTAAAATTTTTGTAACTTAAAAAGGCATGAAAAATGATACCGACCACTA
ACTAATCAACA[T/C]TCCTTTGCGAATACAGCTTCAGTTCCTAACCAAAAACACATCCCTGCTATTGTCTTGTCT
GGATCCAGATGCAAACATGATTAATTAAGTTTCTGGTGAGGAGTGGGACGAGGGCAGTGGGAACTGATGGCTTGCT
GAGGATCCTTTTCATATACAGGCCTGGGGAGAATCCCTTAGTCTGGGTGCCTATTTCCCTCCACATGTCAGGGCTT
ACCCCGTGCACTGACAGCCACGGGGGCAGTGAGTAGGCTGCAGCCAGACCCATGGAGTGAGGCAGTGAGAGGCTGA
AAGGAGAGGCTCTACAGACAGTACAGGGAGGCTCTTGGTATCTCCCATTGCCACTGCACAGCACAGCACGGCAGAG
TAGCAAACGCATATGCCATGTCTTGTTGGGAGACCTGGGCCCTGCTCCAGACTGCTACCAAGTAGTTTGGGACCTT
GGATGAGCTCTCGAG
```

SUPPLEMENTARY TABLES

Supplementary Table 1: Features of the study sample

	Total	Colombia	Brazil	Chile	Mexico	Peru
**Sample size**	5062	1293	697	1376	1211	485
**Percentage**	100	25.5	13.8	27.2	23.9	9.6
**% Female**	52.7	56.6	68.3	32.4	60.4	63.1
**Age (years)**						
Min	18	18	18	18	18	18
Mean	24.4	24.1	25.4	24.8	24.4	22.2
Max	43	40	43	43	40	40
S.D.	5.5	5.4	6.0	5.4	5.5	5.0
**Age, for Males**						
Min	18	18	18	18	18	18
Mean	24.9	24.9	25.6	24.9	25.0	22.5
Max	43	40	42	43	40	37
S.D.	5.5	5.7	5.9	5.1	5.5	5.0
**Age, for Females**						
Min	18	18	18	18	18	18
Mean	24.0	23.6	25.3	24.5	24.0	21.9
Max	43	40	43	40	40	40
S.D.	5.6	5.1	6.1	5.9	5.5	5.0
**Native Ancestry (%)**						
Min	0.001	1.374	0.001	0.821	0.001	0.240
Mean	42.266	28.999	9.151	48.628	58.749	67.281
Max	99.998	93.840	90.915	99.998	99.998	99.998
S.D.	23.307	9.686	10.744	15.464	19.376	18.362

**European Ancestry (%)**						
Min	0.001	4.161	8.334	0.001	0.001	0.001
Mean	52.951	62.392	84.210	49.285	38.053	29.465
Max	99.998	98.039	99.998	98.792	99.998	98.464
S.D.	22.242	11.990	15.000	15.195	18.737	17.209
**African Ancestry (%)**						
Min	0.001	0.001	0.001	0.001	0.001	0.001
Mean	4.783	8.609	6.639	2.088	3.198	3.254
Max	86.452	86.452	51.347	22.371	21.857	48.669
S.D.	5.835	7.418	7.955	2.057	2.328	4.284

Supplementary Table 2: Correlation of the pinna traits examined and covariates

A) Correlation between the pinna traits examined:

Correlation values are presented in the lower left triangle while corresponding permutation p-values are presented in the upper right triangle. Correlations with significant p-values (<0.001, Bonferroni-adjusted threshold) and their corresponding p-values are indicated in bold. Sample size is 5062.

**Trait**	EP	LA	LS	HR	FoA	CHE	SCoAE	DT	TS	AS
Ear Protrusion		**7.6E-07**	4.5E-01	**9.6E-27**	2.1E-02	1.1E-01	**7.1E-09**	**3.2E-06**	6.2E-01	**1.8E-06**
Lobe Attachment	**-0.074**		**1.9E-286**	**6.1E-09**	**5.8E-04**	9.5E-01	6.3E-01	9.3E-01	2.9E-01	6.1E-02
Lobe Size	0.011	**-0.486**		**6.5E-14**	**1.9E-06**	1.0E-02	1.1E-02	8.7E-02	9.9E-02	7.5E-01
Helix Rolling	**0.159**	**-0.083**	**0.108**		**8.9E-69**	2.7E-03	9.2E-01	**9.8E-14**	4.3E-02	**1.8E-08**
Folding of Antihelix	0.035	**-0.049**	**0.068**	**0.248**		2.2E-03	**2.8E-57**	4.1E-02	7.0E-01	3.2E-03
Crus Helix Expression	-0.024	-0.001	0.037	0.043	0.044		**6.9E-08**	9.1E-01	5.5E-02	**1.0E-08**
Superior Crus of Antihelix Expr.	**-0.087**	0.007	0.037	-0.002	**0.228**	**0.078**		4.8E-01	1.5E-01	**1.5E-08**
Darwin's Tubercle	**0.070**	-0.001	-0.025	**0.108**	0.030	0.002	-0.010		4.3E-01	8.7E-01
Tragus Size	0.007	-0.015	0.024	-0.029	0.006	0.028	0.021	0.011		**4.1E-15**
Antitragus Size	**-0.071**	0.027	-0.005	**-0.081**	0.042	**0.082**	**0.082**	0.002	**0.112**	

Trait abbreviations:

EP	Ear Protrusion
AS	Antitragus Size
FoA	Folding of Antihelix
HR	Helix Rolling
LA	Lobe Attachment
LS	Lobe Size
TS	Tragus Size
CHE	Crus Helix Expression
DT	Darwin's Tubercle
SCoAE	Superior Crus of Antihelix Expression

B) Sex-stratified correlation values:

Trait correlation values stratified by sex are presented in the table below, with values for males (sample size 2394) presented in lower left triangle and values for females presented in upper right triangle (sample size 2668). Significant correlations are highlighted in bold.

**Trait**	EP	LA	LS	HR	FoA	CHE	SCoAE	DT	TS	AS
Ear Protrusion		**-0.093**	-0.001	**0.152**	0.005	-0.016	**-0.101**	**0.141**	0.038	-0.063
Lobe Attachment	**-0.082**		**-0.461**	**-0.110**	-0.043	-0.007	-0.017	-0.006	-0.012	0.039
Lobe Size	0.051	**-0.510**		**0.139**	**0.045**	0.055	0.025	-0.043	0.004	-0.014
Helix Rolling	**0.122**	-0.067	**0.092**		**0.261**	0.016	0.017	**0.085**	-0.018	**-0.087**
Folding of Antihelix	0.027	-0.062	**0.103**	**0.220**		0.042	**0.213**	0.045	0.009	0.041
Crus Helix Expression	-0.011	0.008	0.015	**0.080**	0.052		0.064	0.009	0.005	0.061
Superior Crus of Antihelix Expr.	**-0.034**	0.039	0.039	0.001	**0.263**	**0.087**		-0.024	0.000	**0.075**
Darwin's Tubercle	**0.040**	0.011	-0.014	**0.156**	0.024	-0.012	-0.008		0.005	0.002
Tragus Size	0.027	-0.012	0.037	-0.018	0.018	0.047	0.029	0.004		**0.110**
Antitragus Size	**-0.080**	0.015	0.005	**-0.073**	0.046	**0.104**	**0.088**	0.002	**0.115**	

C) Correlation between pinna traits and covariates:

**Covariate**	EP	LA	LS	HR	FoA	CHE	SCoAE	DT	TS	AS
Sex	**-0.250**	-0.036	**0.055**	**-0.122**	**-0.079**	0.036	**0.089**	**0.072**	**0.091**	0.008
Age	0.012	-0.003	0.003	**-0.079**	0.005	**-0.113**	0.025	-0.035	0.017	**-0.062**
Height	**0.213**	0.044	**-0.089**	**0.089**	**0.066**	-0.043	**-0.076**	-0.028	**-0.078**	-0.027
BMI	**-0.105**	0.048	**0.067**	**-0.052**	-0.011	**-0.074**	-0.046	-0.044	-0.021	0.000
African anc.	**-0.084**	**-0.075**	0.043	**0.126**	**0.058**	**0.060**	0.029	-0.047	-0.002	-0.032
European anc.	0.016	**-0.043**	**-0.084**	**0.058**	**0.068**	**-0.069**	**0.059**	0.023	**-0.101**	**-0.122**
American anc.	0.007	**0.060**	**0.069**	**-0.087**	**-0.079**	**0.050**	**-0.063**	-0.010	**0.097**	**0.125**

anc.= Continental ancestry estimated from the genetic data.

Sex coded as female=1, male=0.

Correlations with significant p-values (<0.001, Bonferroni-adjusted threshold) , obtained by permutation, are highlighted in bold.

Supplementary Table 3: Heritability for the ten pinna traits examined, estimated from the population SNP and trait data obtained here.

Trait	Heritability (%)	S.E. (%)	P-value
Ear Protrusion	61.4	7.3	1.00E-17
Lobe Attachment	30.5	7.2	3.80E-14
Lobe Size	52.7	6.9	1.00E-17
Helix Rolling	57.8	6.7	1.00E-17
Folding of Antihelix	27.0	6.7	1.51E-12
Crus Helix Expression	45.5	8.2	3.49E-12
Superior Crus of Antihelix Expression	25.2	6.4	4.41E-14
Darwin's Tubercle	25.6	6.3	2.63E-12
Tragus Size	24.6	6.6	2.44E-14
Antitragus Size	29.2	7.6	7.36E-13

Supplementary Table 4: –log10(p-values) for index SNPs obtained with mixed linear regression models.

P-values were obtained using random effects mixed linear regression models (using FastLMM) for all pinna traits and the seven index SNPs. The SNPs showing genome-wide significant association are the same as in the primary GWAS analysis. For comparison with Table 1, the table below shows $-\log_{10}$ (p-values).

SNP	EP	AS	FoA	HR	LA	LS	CHE	DT	SCoAE	TS
rs17023457	2.4	7.2	9.7	1.1	0.8	0.1	1.5	0.4	1.1	0.4
rs3827760	8.0	0.1	1.6	10.9	7.3	11.1	0.5	0.4	0.3	3.8
rs2080401	0.2	0.5	0.3	0.6	10.0	9.4	0.7	0.2	0.1	0.8
rs10212419	0.1	0.4	0.1	1.1	1.3	12.4	0.1	2.1	0.0	1.8
rs1960918	0.4	2.6	1.1	7.1	0.8	1.6	0.5	0.6	0.3	2.8
rs263156	0.4	0.1	0.8	1.6	4.3	9.9	0.1	1.9	0.5	0.5
rs1619249	1.4	0.4	7.1	1.5	0.1	0.6	1.3	0.3	0.0	0.9

Supplementary Table 5: False discovery rate (FDR) test of association results

We performed a FDR (false discovery rate) test combining GWAS p-values from all 10 pinna traits examined. As FDR is less conservative than Bonferroni, its significance threshold here is 9.55×10^{-7} instead of 5×10^{-8}. A total of 128 SNPs were significant under FDR (listed below). These identify the same associated regions as in the primary GWAS analyses (the index SNPs of Table 1 are highlighted in bold below).

Chromosome	Position	SNP	Trait	FDR p-value
1	119029083	rs17038321	Fold of antihelix	5.37E-07
1	119030698	rs10923574	Fold of antihelix	4.10E-07
1	119117167	rs10802043	Fold of antihelix	3.28E-07
1	119153514	rs2884876	Fold of antihelix	3.88E-07
1	119188329	rs2360627	Fold of antihelix	2.69E-07
1	119193242	rs2145789	Fold of antihelix	2.24E-07
1	119226884	rs10158862	Fold of antihelix	4.48E-07
1	119253426	rs2755140	Fold of antihelix	7.39E-07
1	119259262	rs6692676	Fold of antihelix	4.40E-07
1	119289072	rs10923662	Fold of antihelix	4.18E-07
1	119291382	rs1570816	Fold of antihelix	4.92E-07
1	119300941	rs9787392	Fold of antihelix	6.86E-07
1	119316263	rs10923673	Fold of antihelix	7.09E-07
1	119318644	rs10923674	Fold of antihelix	7.54E-07
1	119392083	rs868157	Fold of antihelix	7.46E-07
1	119394195	rs12408957	Fold of antihelix	5.60E-07
**1**	**119875730**	**rs17023457**	**Fold of antihelix**	8.21E-08
**1**	**119875730**	**rs17023457**	**Antitragus size**	3.73E-07
1	119928193	rs3207643	Fold of antihelix	4.63E-07
2	85545490	rs7428	Ear Protrusion	7.76E-07
2	108883866	rs13388627	Lobe Size	5.30E-07
2	108884042	rs4638749	Lobe Size	5.89E-07
2	108885895	rs6542759	Lobe Size	2.09E-07
2	108905048	rs2305485	Lobe Size	5.22E-07
2	108912673	rs13410305	Lobe Size	1.34E-07
2	108916044	rs4149423	Lobe Size	1.04E-07
2	108926967	rs2198466	Lobe Size	1.19E-07
2	108932436	rs1989096	Lobe Size	9.40E-07
2	108938552	rs1879495	Helix Rolling	3.66E-07
2	108938735	rs12477830	Helix Rolling	1.94E-07
2	108938735	rs12477830	Lobe Size	5.07E-07
2	108940336	rs12476238	Helix Rolling	1.64E-07
2	108940336	rs12476238	Lobe Size	5.82E-07
2	108946178	rs6709159	Helix Rolling	3.95E-07
2	108946860	rs6709978	Helix Rolling	8.13E-07
2	108946860	rs6709978	Lobe Size	8.66E-07
2	108988625	rs10167564	Lobe Size	4.55E-07

2	108994808	rs1402467	Lobe Size	8.43E-07
2	108995325	rs4149432	Lobe Size	9.25E-07
2	108997262	rs4149433	Helix Rolling	3.73E-08
2	108997262	rs4149433	Lobe Size	5.22E-08
2	108997262	rs4149433	Ear Protrusion	1.79E-07
2	108997262	rs4149433	Lobe Attachment	3.36E-07
2	108999786	rs4149436	Lobe Size	6.04E-07
2	109002048	rs4149438	Lobe Size	9.48E-07
2	109004835	rs17269356	Lobe Size	8.73E-07
2	109006665	rs13021399	Lobe Size	3.13E-07
2	109006665	rs13021399	Ear Protrusion	4.25E-07
2	109028656	rs6755756	Lobe Size	8.28E-07
2	109055717	rs10203795	Ear Protrusion	5.15E-07
2	109055908	rs10169264	Ear Protrusion	5.75E-07
2	109055908	rs10169264	Helix Rolling	6.94E-07
2	109066424	rs2378113	Ear Protrusion	2.84E-07
2	109150164	rs11123706	Lobe Size	3.51E-07
2	109150164	rs11123706	Ear Protrusion	4.85E-07
2	109150164	rs11123706	Helix Rolling	7.16E-07
2	109156275	rs6754683	Ear Protrusion	7.69E-07
2	109156275	rs6754683	Helix Rolling	8.36E-07
2	109198530	rs13413437	Ear Protrusion	4.78E-07
2	109198530	rs13413437	Helix Rolling	8.51E-07
**2**	**109513601**	**rs3827760**	**Helix Rolling**	2.98E-08
**2**	**109513601**	**rs3827760**	**Lobe Size**	4.48E-08
**2**	**109513601**	**rs3827760**	**Ear Protrusion**	1.27E-07
**2**	**109513601**	**rs3827760**	**Lobe Attachment**	2.31E-07
2	109544052	rs13397666	Lobe Attachment	6.42E-07
2	109552878	rs13427222	Lobe Attachment	4.70E-07
2	109552878	rs13427222	Lobe Size	7.31E-07
2	109556365	rs6542787	Lobe Size	4.33E-07
2	109556365	rs6542787	Lobe Attachment	6.27E-07
2	109557099	rs260711	Lobe Size	5.52E-07
2	109562495	rs260714	Lobe Size	7.61E-07
2	109566759	rs260698	Lobe Size	5.45E-07
2	109571440	rs260705	Lobe Size	6.57E-07
2	109579738	rs260690	Lobe Size	2.16E-07
2	109579738	rs260690	Helix Rolling	2.39E-07
2	109582357	rs5021634	Lobe Size	3.21E-07
2	109582357	rs5021634	Lobe Attachment	5.67E-07
2	109599256	rs260674	Tragus size	7.01E-07
2	109669494	rs6737482	Helix Rolling	1.57E-07
2	109674911	rs383993	Helix Rolling	2.91E-07
2	109706076	rs4676237	Helix Rolling	1.72E-07
2	109706076	rs4676237	Ear Protrusion	8.88E-07
2	109742307	rs6542797	Helix Rolling	9.10E-07
2	109754468	rs7588387	Helix Rolling	1.12E-07
2	109754468	rs7588387	Ear Protrusion	6.19E-07

2	109759168	rs7567615	Helix Rolling	9.70E-08
2	109759168	rs7567615	Ear Protrusion	9.03E-07
2	110381572	rs10496434	Helix Rolling	9.18E-07
2	110399823	rs7580778	Lobe Size	6.64E-07
2	171534221	rs10192049	Lobe Attachment	2.76E-07
2	171534221	rs10192049	Lobe Size	7.24E-07
2	171539261	rs1035150	Lobe Attachment	1.87E-07
2	171539261	rs1035150	Lobe Size	8.95E-07
**2**	**171540823**	**rs2080401**	**Lobe Attachment**	5.97E-08
**2**	**171540823**	**rs2080401**	**Lobe Size**	1.42E-07
2	171548493	rs7574074	Lobe Attachment	2.61E-07
2	171548493	rs7574074	Lobe Size	3.06E-07
3	135281668	rs7612415	Lobe Size	9.33E-07
3	138985300	rs1602631	Lobe Size	7.98E-07
3	138997688	rs9866054	Lobe Size	2.24E-08
**3**	**139004920**	**rs10212419**	**Lobe Size**	7.46E-09
4	102276682	rs6845263	Superior Crus of antihelix expression	8.06E-07
4	151188215	rs1599167	Helix Rolling	6.49E-07
4	151204693	rs11944163	Helix Rolling	6.34E-07
**4**	**151222266**	**rs1960918**	**Helix Rolling**	4.03E-07
4	151231371	rs1813134	Helix Rolling	6.72E-07
4	151238436	rs962626	Helix Rolling	5.00E-07
4	155397517	rs4696584	Fold of antihelix	5.97E-07
4	155413362	rs1873367	Fold of antihelix	8.58E-07
6	142879589	rs605790	Lobe Size	8.95E-08
6	142879693	rs702293	Lobe Size	7.46E-08
6	142882955	rs263114	Lobe Size	2.54E-07
**6**	**142907515**	**rs263156**	**Lobe Size**	1.49E-08
6	142921498	rs13217677	Lobe Size	1.49E-07
6	142922061	rs2077836	Lobe Size	6.72E-08
6	142949260	rs9373355	Lobe Size	2.98E-07
6	142965039	rs6928084	Lobe Size	3.81E-07
6	142969570	rs9390016	Lobe Size	6.79E-07
6	142976691	rs7771119	Lobe Size	2.01E-07
6	142990718	rs1396898	Lobe Size	3.43E-07
6	142993151	rs7753052	Lobe Size	2.46E-07
7	28391047	rs11772815	Fold of antihelix	7.91E-07
9	31394025	rs7857709	Lobe Size	8.21E-07
9	31394275	rs7873690	Lobe Size	9.55E-07
10	42917476	rs2489715	Helix Rolling	7.83E-07
10	111635212	rs3818285	Superior Crus of antihelix expression	8.80E-07
**18**	**49190644**	**rs1619249**	**Fold of antihelix**	3.58E-07
18	55175143	rs10503015	Helix Rolling	6.12E-07

Supplementary Table 6: Meta-analysis p-values for index SNPs associated with the pinna traits examined

Country-stratified meta-analysis p-values for the SNPs in Table 1 are shown below. Genome-wide significant associations are all replicated (here highlighted in red). Cochran's Q statistic was computed for each trait to test for effect size heterogeneity across countries, and the p-value was not significant in any case. Forest plots for each SNP are shown in Supplementary Figure 6A, and Manhattan plots in Figure 6B.

Region	SNP	Ear Protrusion	Antitragus Size	Folding of Antihelix	Helix Rolling	Lobe Attachment	Lobe Size
1p12	rs17023457	1.7E-03	**3.1E-08**	**3.0E-11**	2.6E-02	2.7E-01	4.1E-01
2q12.3	rs3827760	**5.8E-09**	7.5E-01	1.7E-02	**2.8E-11**	**1.3E-07**	**3.6E-13**
2q31.1	rs2080401	6.3E-01	3.8E-01	2.8E-01	2.0E-01	**1.9E-11**	**4.8E-11**
3q23	rs10212419	5.7E-01	2.5E-01	8.2E-01	6.9E-02	6.5E-02	**5.6E-12**
4q31.3	rs1960918	6.1E-01	3.1E-03	9.8E-02	**2.1E-07**	1.4E-01	4.0E-02
6q24.2	rs263156	5.1E-01	8.6E-01	1.2E-01	9.5E-03	1.1E-05	**1.6E-12**
18q21.2	rs1619249	5.7E-02	2.7E-01	**1.8E-07**	4.9E-02	5.9E-01	5.6E-01

Sex- stratified Meta-analysis p-values for the same set of SNPs and traits. Only the previously identified associations are shown. Manhattan plots are shown in Supplementary Figure 6C.

Region	SNP	Ear Protrusion	Antitragus Size	Folding of Antihelix	Helix Rolling	Lobe Attachment	Lobe Size
1p12	rs17023457		1.0E-07	5.6E-11			
2q12.3	rs3827760	2.0E-09			1.3E-12	4.6E-08	4.6E-13
2q31.1	rs2080401					3.2E-11	7.6E-11
3q23	rs10212419						1.3E-12
4q31.3	rs1960918				7.9E-07		
6q24.2	rs263156						3.8E-11
18q21.2	rs1619249			2.8E-08			

Supplementary Table 7: Prediction of pinna traits from the genotypes at the seven index SNPs

We predicted phenotypes from the genotypes at the seven index SNPs in Table 1 (with an additive encoding of the minor allele: i.e. 0, 1 or 2 copies) using linear regression or neural network modelling and including the same covariates as for the GWAS (age, sex, height, BMI). The accuracy of this prediction was evaluated by examining the correlation between the observed and predicted trait scores. The neural network model being nonlinear provides slightly greater prediction accuracy.

**Trait**	**Correlation**	
	Linear model	Neural network
Ear Protrusion	41.4	43.6
Lobe Attachment	20.5	26.0
Lobe Size	30.5	33.5
Helix Rolling	35.0	37.0
Folding of Antihelix	23.0	28.2
Crus Helix Expression	20.0	23.9
Superior Crus of Antihelix Expression	14.1	17.4
Darwin's Tubercle	19.4	24.9
Tragus Size	20.2	26.1
Antitragus Size	17.5	18.6

Supplementary Table 8: Derived allele frequency (A) and effect size (B) for index SNPs showing genome-wide significant association with pinna shape.

A)

Supplementary Table 8A below identifies the ancestral and derived alleles, and presents the derived allele frequency for each SNP in several populations at each index SNP of Table 1 (main text). Intensity of orange color is graded for increasing derived allele frequency. Population acronyms: CEU (U.S. residents with northern and western European ancestry), YRI (Yoruba of Ibadan, Nigeria), CHB (Han Chinese in Beijing, China), JPT (Japanese in Tokyo, Japan) and MEX (Mexican ancestry in Los Angeles, California). Allele frequencies for these HAPMAP samples were obtained from dbSNP (http://www.ncbi.nlm.nih.gov/SNP/). NAM denotes a set of Native Americans genotyped here. Candela denotes the GWAS study sample characterized here.

Region	SNP	Ancestral allele	Derived allele	NAM	CEU	YRI	CHB	JPT	MEX	Candela
1p12	rs17023457	T	C	0.74	0.15	0.00	0.44	0.44	0.41	0.39
2q12.3	rs3827760	A	G	0.98	0.00	0.00	0.94	0.80	0.42	0.40
2q31.1	rs2080401	A	C	0.88	0.32	0.39	0.37	0.25	0.54	0.60
3q23	rs10212419	G	A	0.94	0.21	0.09	0.46	0.41	0.54	0.52
4q31.3	rs1960918	G	A	0.32	0.64	0.46	0.26	0.35	0.32	0.43
6q24.2	rs263156	A	C	0.88	0.56	0.02	0.73	0.79	0.66	0.67
18q21.2	rs1619249	G	A	0.65	0.85	0.51	0.52	0.42	0.81	0.81

B)

Supplementary Table 8B presents the phenotypic effect size (i.e. beta regression coefficient) for the derived allele at each significantly associated SNP from Table 1 of the main text. Increasing expression of each trait was scored numerically as 0, 1 or 2 (Supplementary Figure 1). To aid interpretation of the phenotypic effects of the associated alleles, below we refer qualitatively to the phenotypic value range for each trait.

Region	SNP	Derived allele	Ear Protrusion (flat – protruded)	Antitragus Size (small – large)	Folding of Antihelix (weak – prominent)	Helix Rolling (weak – prominent)	Lobe Attachment (attached – detached)	Lobe Size (small – large)
1p12	rs17023457	C		-0.060	-0.087			
2q12.3	rs3827760	G	-0.108			-0.103	-0.066	-0.117
2q31.1	rs2080401	C					-0.071	-0.096
3q23	rs10212419	A						0.107
4q31.3	rs1960918	A				-0.066		
6q24.2	rs263156	C						0.109
18q21.2	rs1619249	A			0.091			

Supplementary Table 9: Intraclass correlations coefficients (ICCs) of pinna trait scores.

We calculated the ICC for pinna trait scores following the definition of Shrout & Fleiss (1979). This approach uses a two-way mixed effects ANOVA model, with two scores for an individual (from double scoring by one rater or from scores by two raters) as a fixed effect, and variation across individuals as a random effect. Scores from a set of photographs for 100 individuals were used for calculating ICCs for each pinna trait phenotyped. The photographs were scored twice by two raters, independently, two weeks apart.

	**ICC**		
**Trait**	Intra-observer-1	Intra-observer-2	Inter-observer
Ear Protrusion	81%	53%	69%
Lobe attachment	68%	73%	54%
Lobe size	65%	66%	62%
Helix rolling	59%	70%	43%
Crus helix expression	55%	45%	40%
Superior Crus of antihelix expression	52%	66%	43%
Folding of antihelix	54%	57%	59%
Darwin's tubercle	61%	85%	73%
Tragus size	40%	51%	58%
Antitragus size	50%	71%	59%

Ref: Supplementary Reference 3.

Supplementary Table 10: Reliability analysis of mouse landmarking

The top head view photographs were landmarked separately by two raters, and for each landmark the average distance between two points placed by two different raters was calculated across all samples. The values for each landmark are shown below (in pixel units). Average head size was 479 pixels in length (±24), so the magnitude of fluctuation is 1% or lower.

Landmark	Variation (pixels)
1	0.22
2	0.47
3	4.44
4	2.50
5	2.29
6	1.30
7	1.47
8	0.41
9	0.17
10	0.25

The side view photographs were similarly landmarked by two raters, and average deviation between two raters are given below for each landmark (in pixel units). Average head size was 632 pixels (±31). The landmarks are shown in black while semi-landmarks are shown in gray. As expected, semi-landmarks have higher deviation because unlike landmarks they are not defined to be placed on a specific point, rather on any part of the contour of pinna.

Landmark	Variation (pixels)
1	7.70
2	5.42
3	4.84
4	8.59
5	7.59
6	7.90
7	9.97
8	11.01
9	12.40
10	12.53
11	15.24
12	11.57
13	10.38
14	11.30
15	11.40
16	11.57
17	14.06
18	13.39

19	8.64
20	7.90
21	7.28
22	15.53
23	16.92
24	22.04
25	18.53
26	16.19
27	17.16

SUPPLEMENTARY NOTES

Supplementary Note 1: Effect of *Edar* genotype on mutant mouse pinna morphology.

We compared pinna morphology between wild-type and mutant mice. The loss of function $Edar^{dlJ}$ line carries the downless Jackson allele (encoding Edar p. E379K). The gain of function $Edar^{Tg951}$ line carries approximately 16 copies of the Edar gene copies of the entire Edar locus on a 200 Kb yeast artificial chromosome (homozygous transgenic animals therefore carry about 34 copies of the gene in total). A multivariate linear regression analysis was performed in which the EDAR locus genotypes were considered a binary variable: $Edar^{dlJ}/Edar^{dlJ}$ homozygotes were considered as level 1 and the other 3 genotypes were grouped into level 0. This choice was supported by inspection of the boxplots in Supplementary Figure 11 , where only the homozygous EdardlJ category seems distinct. Output from the analyses performed in R is provided below. For confirmation, a regression analysis in which each genotype is considered a separate factor produced similar results (not shown).

Regression of ear protrusion angle vs. genotype

```
Call:
lm(formula = angle ~ age + sex + homo + body_length +
    weight + head_length + head_width, data = mice)

Coefficients:
            Estimate Std. Error t value Pr(>|t|)
(Intercept)   14.026    130.701   0.107 0.915170
age            7.697      7.351   1.047 0.302450
sexF           6.139      5.487   1.119 0.271028
homo         -33.785      7.833  -4.313 0.000131 ***
body_length   -3.025      2.156  -1.403 0.169714
weight         6.201      5.480   1.132 0.265735
head_length    5.947      4.882   1.218 0.231536
head_width     5.015      7.569   0.663 0.512117
---
Signif. codes:  0 ‘***’ 0.001 ‘**’ 0.01 ‘*’ 0.05 ‘.’ 0.1 ‘ ’ 1

Residual standard error: 16.45 on 34 degrees of freedom
Multiple R-squared:   0.56, Adjusted R-squared:  0.4694
F-statistic: 6.181 on 7 and 34 DF,  p-value: 0.0001042

Analysis of Variance Table

Response: angle
            Df Sum Sq Mean Sq F value    Pr(>F)
age          1  408.7   408.7  1.5094   0.22766
sexF         1  673.1   673.1  2.4861   0.12412
homo         1 8945.2  8945.2 33.0364 1.832e-06 ***
body_length  1   57.0    57.0  0.2104   0.64935
weight       1 1048.0  1048.0  3.8705   0.05734 .
```

```
head_length  1  464.1   464.1  1.7139   0.19926
head_width   1  118.8   118.8  0.4389   0.51212
Residuals   34 9206.1   270.8
---
Signif. codes:  0 ‘***’ 0.001 ‘**’ 0.01 ‘*’ 0.05 ‘.’ 0.1 ‘ ’ 1
```

Regression of ear tip distance (as a proportion of head width) vs. genotype categories:

R regression output:

```
lm(formula = tip_prop ~ age + sex + homo + body_length + weight +
    head_length + head_width, data = mice)

Residuals:
     Min       1Q   Median       3Q      Max
-0.37794 -0.07331  0.01781  0.11324  0.24070

Coefficients:
             Estimate Std. Error t value Pr(>|t|)
(Intercept)  0.761260   1.298694   0.586 0.561630
age          0.042402   0.073039   0.581 0.565383
sexF         0.049551   0.054521   0.909 0.369833
homo        -0.311210   0.077832  -3.998 0.000325 ***
body_length -0.005454   0.021425  -0.255 0.800603
weight       0.003212   0.054447   0.059 0.953307
head_length  0.065453   0.048511   1.349 0.186179
head_width  -0.019124   0.075209  -0.254 0.800811
---
Signif. codes:  0 ‘***’ 0.001 ‘**’ 0.01 ‘*’ 0.05 ‘.’ 0.1 ‘ ’ 1

Residual standard error: 0.1635 on 34 degrees of freedom
Multiple R-squared:  0.4713,     Adjusted R-squared:  0.3624
F-statistic: 4.329 on 7 and 34 DF,  p-value: 0.001609

Analysis of Variance Table

Response: tip_prop
            Df  Sum Sq Mean Sq F value    Pr(>F)
age          1 0.00053 0.00053  0.0198    0.8890
sexF         1 0.05664 0.05664  2.1188    0.1547
homo         1 0.69762 0.69762 26.0955 1.243e-05 ***
body_length  1 0.00157 0.00157  0.0587    0.8100
weight       1 0.00494 0.00494  0.1847    0.6701
head_length  1 0.04715 0.04715  1.7638    0.1930
head_width   1 0.00173 0.00173  0.0647    0.8008
Residuals   34 0.90893 0.02673
---
Signif. codes:  0 ‘***’ 0.001 ‘**’ 0.01 ‘*’ 0.05 ‘.’ 0.1 ‘ ’ 1
```

Regression of 2D pinna landmarks PC1 vs. genotype categories:

R regression output:

```
Call:
lm(formula = PC1 ~ age + sex + homo + body_length +
    weight + head_length + head_width, data = mice)

Coefficients:
             Estimate Std. Error t value Pr(>|t|)
(Intercept)  0.492906   0.798958   0.617 0.541387
age         -0.032081   0.044934  -0.714 0.480125
sexF        -0.019749   0.033541  -0.589 0.559895
homo        -0.181051   0.047882  -3.781 0.000603 ***
body_length -0.009849   0.013181  -0.747 0.460062
weight       0.051592   0.033496   1.540 0.132759
head_length  0.010397   0.029844   0.348 0.729710
head_width  -0.042692   0.046269  -0.923 0.362671
---
Signif. codes:  0 ‘***’ 0.001 ‘**’ 0.01 ‘*’ 0.05 ‘.’ 0.1 ‘ ’ 1

Residual standard error: 0.1006 on 34 degrees of freedom
Multiple R-squared:  0.5355,     Adjusted R-squared:  0.4398
F-statistic: 5.599 on 7 and 34 DF,  p-value: 0.0002365

Analysis of Variance Table

Response: PC1
            Df  Sum Sq  Mean Sq F value    Pr(>F)
age          1 0.04427 0.044273  4.3757    0.0440 *
sexF         1 0.00003 0.000030  0.0030    0.9567
homo         1 0.30350 0.303503 29.9970 4.123e-06 ***
body_length  1 0.01783 0.017831  1.7624    0.1932
weight       1 0.02173 0.021734  2.1481    0.1519
head_length  1 0.00057 0.000569  0.0563    0.8139
head_width   1 0.00861 0.008614  0.8514    0.3627
Residuals   34 0.34401 0.010118
---
Signif. codes:  0 ‘***’ 0.001 ‘**’ 0.01 ‘*’ 0.05 ‘.’ 0.1 ‘ ’ 1
```

SUPPLEMENTARY REFERENCES

1. Alexander DH, Novembre J, Lange K (2009) Fast model-based estimation of ancestry in unrelated individuals. Genome Res 19: 1655-1664.
2. Ruiz-Linares et al., Admixture In Latin America: Geographic Structure, Phenotypic Diversity And Self-Perception Of Ancestry Based On 7,342 Individuals. PLoS Genetics, 2014.
3. P. E. Shrout & Joseph L. Fleiss (1979). "Intraclass Correlations: Uses in Assessing Rater Reliability". Psychological Bulletin 86 (2): 420–428.
